# Substrate Fragmentation for the Design of *M. tuberculosis* CYP121 Inhibitors

**DOI:** 10.1002/cmdc.201600248

**Published:** 2016-07-19

**Authors:** Madeline E. Kavanagh, Janine L. Gray, Sophie H. Gilbert, Anthony G. Coyne, Kirsty J. McLean, Holly J. Davis, Andrew W. Munro, Chris Abell

**Affiliations:** ^1^ Department of ChemistryThe University of CambridgeLensfield RoadCambridgeCB2 1EWUK; ^2^ Centre for Synthetic Biology of Fine and Specialty Chemicals (SYNBIOCHEM)Manchester Institute of BiotechnologyFaculty of LifeSciencesThe University of ManchesterManchesterM1 7DNUK

**Keywords:** drug discovery, enzymes, fragment-based methods, substrate analogues, tuberculosis

## Abstract

The cyclo‐dipeptide substrates of the essential *M. tuberculosis* (*Mtb*) enzyme CYP121 were deconstructed into their component fragments and screened against the enzyme. A number of hits were identified, one of which exhibited an unexpected inhibitor‐like binding mode. The inhibitory pharmacophore was elucidated, and fragment binding affinity was rapidly improved by synthetic elaboration guided by the structures of CYP121 substrates. The resulting inhibitors have low micromolar affinity, good predicted physicochemical properties and selectivity for CYP121 over other *Mtb* P450s. Spectroscopic characterisation of the inhibitors′ binding mode provides insight into the effect of weak nitrogen‐donor ligands on the P450 heme, an improved understanding of factors governing CYP121–ligand recognition and speculation into the biological role of the enzyme for *Mtb*.

## Introduction

Enzymes have evolved to bind substrates specifically. However, the binding energetics of enzyme–substrate interactions are optimised for catalytic efficiency, and therefore implicitly, they should not bind too tightly. Consequently, the contribution to the free energy of binding of individual structural motifs will be unevenly distributed over the enzyme–substrate complex and the relative importance of these interactions are difficult to assess when combined in a large molecule.^1^, ^2^ In this way, large (>250 Da) substrates are analogous to large molecule drug leads or hits that are identified from a high throughput screen. These compounds typically bind in the low micromolar range by making multiple suboptimal interactions, and as such, may not represent the best starting points for elaboration.^3^, ^4^, ^5^, ^6^, ^7^

The deconstruction of large molecules into their representative fragments can provide an assessment of the binding contributions of individual structural motifs, identify the minimal pharmacophore required for biological activity and provide insight into the fundamental basis of enzyme–substrate recognition.^8^, ^9^, ^10^, ^11^, ^12^ This principle can be applied to deconvoluting the binding of a large substrate or a hit from a high throughput screen. Fragment‐based methods are now firmly established as valuable techniques for identifying ligand efficient small molecules that can act as leads for drug development.^13^, ^14^ The application of fragment screening as a tool to assess target druggability, identify binding hotspots, and interrogate biological systems is also being increasingly appreciated.^15^, ^16^, ^17^ The utility of fragments for these applications is a consequence of their small size and inherent ability to probe macromolecular surfaces more effectively than larger compounds.^4^, ^6^

The question as to whether fragments of a larger ligand maintain the same binding interactions when they are disconnected has been addressed in recent years.^8^, ^9^, ^10^, ^11^, ^12^, ^18^, ^19^, ^20^ The size and structures of the disconnected fragments, the choice of disconnection, properties of the target macromolecule, and requirements for cofactors or binding cooperativity, are all factors that have contributed to the variation in the results observed. What is consistently apparent though, is that fragments derived from large ligands bind preferentially to energetic hotspots and, regardless whether this binding mode recapitulates that of the larger ligand, characterisation of these interactions provides valuable insight for subsequent ligand design.^21^, ^22^ Furthermore, lead deconstruction or fragmentation has resulted in the identification of novel chemical scaffolds for use as drug leads and previously unknown binding sites, as well as contributing to understanding the mechanisms of biomolecular recognition or catalysis; outcomes which are ultimately, of more value.^8^, ^9^

**Figure 1:**
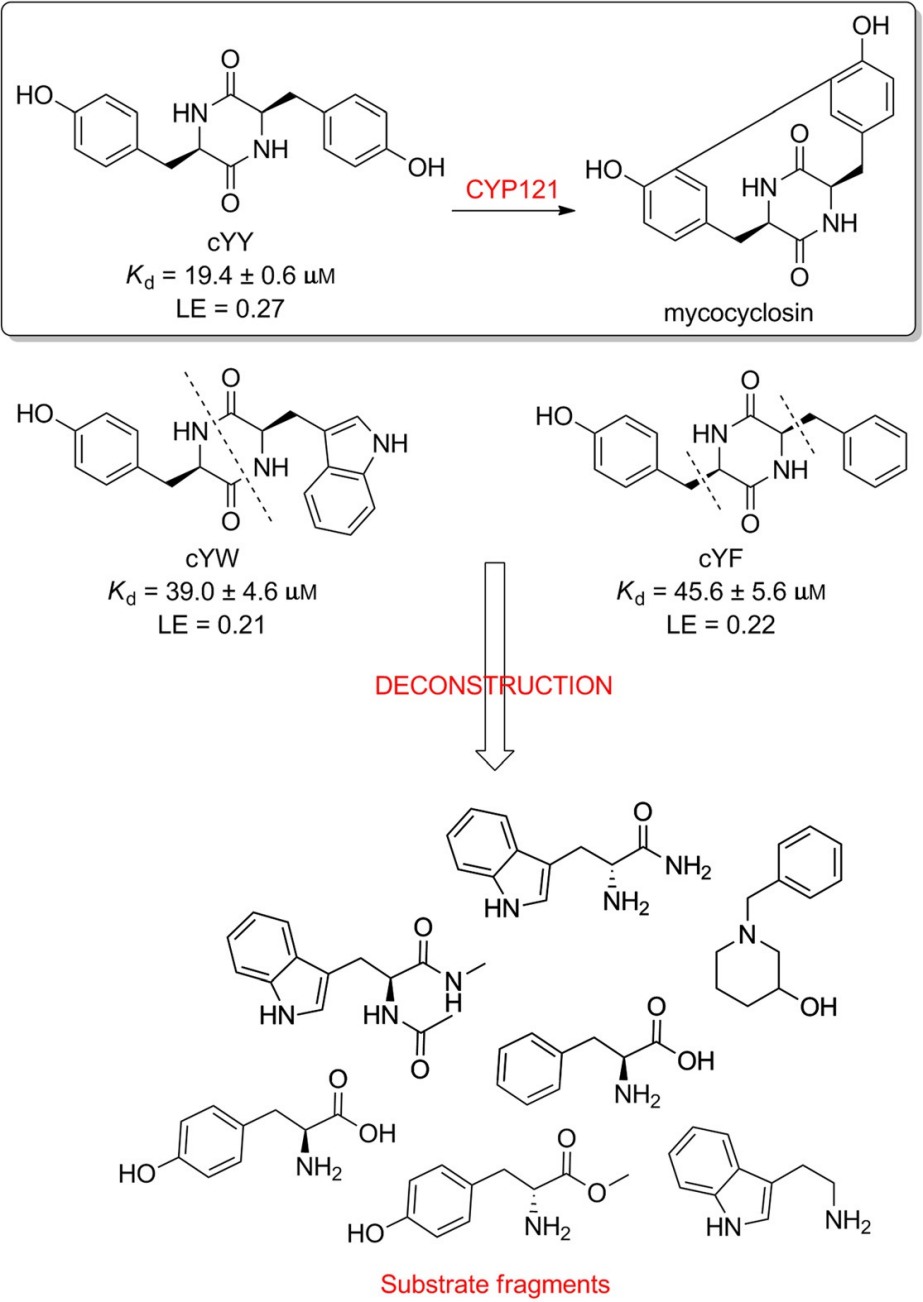
Cyclo‐dipeptide CYP121 substrates cyclo‐l‐Tyr‐l‐Tyr (cYY), cyclo‐l‐Tyr‐l‐Trp (cYW) and cyclo‐l‐Tyr‐l‐Trp (cYW) are deconstructed into a library of representative fragments (selected fragments shown). CYP121 catalyses the C−H oxidation of cYY to form mycocyclosin.^31^, ^33^ The dissociation constants (*K*_d_) and ligand efficiency (LE) values of substrates are noted.

Although the retrospective deconstruction of successful drug candidates, lead compounds and enzyme substrates has been detailed in the literature,^9^, ^10^, ^11^, ^12^, ^19^, ^23^, ^24^ there are few examples where the fragments derived in this way have been pursued for subsequent ligand development.^25^ Here, the dipeptide substrates of the cytochrome P450 enzyme CYP121 from *Mycobacterium tuberculosis* (*Mtb*) have been deconstructed as a route to identifying novel scaffolds for inhibitor development. The CYP121 gene is essential for *Mtb* survival in vitro and the enzyme is believed to be the molecular target responsible for the anti‐tubercular efficacy of azole antifungal compounds.^26^, ^27^, ^28^, ^29^, ^30^ CYP121 catalyses an unusual biosynthetic C‐H activation reaction that cyclises the dipeptide cyclo‐l‐Tyr‐l‐Tyr (cYY) to form mycocyclosin (Figure 1).^31^ The aromatic dipeptides cyclo‐l‐Tyr‐l‐Trp (cYW) and cyclo‐l‐Tyr‐l‐Phe (cYF), which are the minor products of the cyclo‐dipeptide synthetase Rv2275 that is encoded directly upstream of the CYP121 gene (*rv2276*) in *Mtb* H37Rv,^32^ were also recently reported to bind to CYP121 and catalytic turnover was detected for cYW.^33^

We have previously reported the development of potent ligands against CYP121, however, these compounds had suboptimal cellular activity, owing to either poor permeability or efflux.^25^ In the current study, it was reasoned that the deconstruction of the CYP121 substrates into their component fragments might provide access to novel scaffolds and insight into the interactions governing enzyme‐ligand recognition. Substrate fragments were preferentially identified to bind to CYP121 when they were screened as part of a library of chemically similar compounds by thermal shift and UV/vis spectroscopy assays. A substrate fragment that exhibited an unexpected inhibitor‐like mode of binding was identified, and synthetically combining the inhibitory pharmacophore of this novel fragment scaffold with inspiration from the structures of substrates allowed for fragment binding affinity to be improved. Detailed spectroscopic characterisation of the ligand binding mode and analysis of the SAR of these compounds provides insight into the requirements of CYP121–ligand binding interactions, the possible biological role of CYP121 for *Mtb* and contributes to understanding the effect of weak nitrogen donor ligands on the P450 heme.

## Results and Discussion

### Substrate deconstruction and fragment screening

The CYP121 substrates cYY, cYW and cYF were conceptually deconstructed and a collection of commercially available fragments that represented their different structural motifs was compiled (Figure 1). These substrate fragments were combined into a library of fragments that had broadly similar chemical properties, specifically the combination of one aromatic ring with a polar aliphatic or heterocyclic motif, in order to assess whether the substrate fragments were preferentially identified as hits. This library of 65 fragments was screened against *Mtb* CYP121 using a thermal shift assay. Seven hits were identified that increased the denaturation temperature (*T*_m_) of CYP121 by more than 1 °C (Table 1). Five of the hits **1 a**–**e** were derivatives of tyrosine, tryptophan or phenylalanine, while the remaining two fragments **1 f** and **1 g** contained morpholine rings bound to phenyl‐ or benzyl‐amine groups. Three weakly binding (Δ*T*_m_=1 °C) fragments were also identified, that had more diverse scaffolds, including a cyclic sulfone, methylamine‐tetrahydropyran and phenylcyclopropane carboxylic acid (Table S1, Supporting Information). None of the l‐amino acids tyrosine, tryptophan or phenylalanine were identified as hits, nor were a range of amide derivatives of the amino acids, which had been included in the fragment library to mimic cleavage of the diketopiperazine ring of the CYP121 substrates. However, the methyl esters of d‐tryptophan and d‐tyrosine increased in the *T*_m_ of CYP121 by +4.5 and +3 °C, respectively. The methyl ester of l‐phenylalanine was also identified as a hit, but produced a smaller Δ*T*_m_ of +1.5 °C.

**Table 1:** Fragments identified by thermal shift and UV/vis spectroscopy to bind to *Mtb* CYP121.

Fragment		Δ*T*_m_ [°C]^[a]^	Δ*λ*_max_ [nm]^[b]^
**1 a**	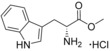	+4.5	+4.5
**1 b**	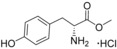	+3	0
**1 c**	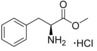	+1.5	0
**1 d**	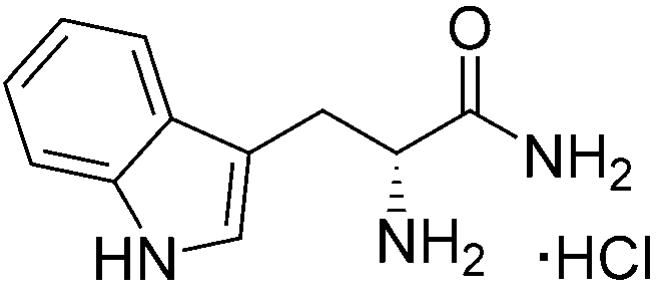	+2.5	0
**1 e**	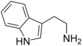	+1.5	0
**1 f**	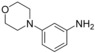	+2.0	+4
**1 g**	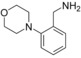	+2.0	0

[a] Change in the denaturation temperature (*T*_m_) of CYP121 relative to a DMSO (5 % *v*/*v*) control (*T*_m_=49.0 °C); fragments were screened at 5 mm against 5 μm CYP121. [b] Magnitude of change in the maximum wavelength of the Soret band of the CYP121 (5 μm) optical spectrum, calculated relative to a DMSO (1 % *v*/*v*) control (Δ*λ*_max_=416.5 nm); fragments were screened at 2 mm.

The seven fragment hits were subsequently analysed by UV/vis spectroscopy to detect binding interactions with the heme prosthetic group of CYP121. Ligands that bind to the P450 heme to form a more strongly coordinated complex that the resting state enzyme, either by direct coordination of the ferric iron, or indirect coordination via the axial water ligand, cause a red shift (type II) in the maximum wavelength (*λ*_max_) of the Soret band of the P450 optical spectrum. In contrast, ligands which bind in the vicinity of the heme and displace the axial water ligand, but do not ligate the ferric iron, cause a blue shift (type I) in the Soret *λ*_max_.^34^ Only two of the seven hit fragments identified in the thermal shift assay produced observable changes in the CYP121 optical spectrum. One of these fragments, compound **1 f** contained an aniline functional group, which we have previously reported as a preferred CYP121 heme binding motif.^25^, ^35^ The second, was the d‐tryptophan methyl ester **1 a**, which produced a red shift in the Soret *λ*_max_ of +4.5 nm. The remaining fragments, including the methyl ester of d‐tyrosine **1 b**, did not perturb the optical spectrum of CYP121. This suggested that the binding interactions of these fragments must occur distal to the heme, or be too weak to perturb the heme coordination sphere of the resting state enzyme. The preference of CYP121 for d‐tryptophan methyl ester **1 a** over its respective d‐tyrosine methyl ester analogue, which was demonstrated in both thermal shift and UV/vis assays, was surprising because cYY is considered to be the preferred substrate of CYP121. The cyclo‐dipeptides cYW and cYF are produced in only small quantities when the cyclo‐dipeptide synthetase Rv2275 is expressed in *E. coli*.^32^ However, their binding affinities (*K*_d_ values) for CYP121 are in the same order of magnitude (*K*_d_=39–46 μm) as the *K*_d_ value of cYY (*K*_d_=19 μm) and thus, the preference observed here for tryptophan could indicate a broader substrate profile for CYP121 than previously recognised. The type II shift in the CYP121 optical spectrum that was generated by tryptophan methyl ester **1 a** were contrary to expectations for a substrate‐like compound. Type II optical spectra are characteristic of ligands that have “inhibitor‐like” interactions with the heme prosthetic group and are considered to indicate stabilisation of the inactive, low‐spin state of P450s.^34^ In contrast, the cyclo‐dipeptides cYY, cYW and cYF all produce type I, or “substrate‐like” shifts in the optical spectrum of CYP121, which are consistent with a change in spin state of the heme.^33^, ^34^ It was proposed that combining the inhibitor‐like binding mode of fragment **1 a** with structural features that contribute to the binding affinity of the cyclo‐dipeptides substrates might result in a novel class of CYP121 inhibitors. In addition, fragment **1 a** was found to bind to CYP121 on an unusually slow time scale, causing a progressively greater Δ*λ*_max_ of the Soret of +2 nm to +4.5 nm over a 3 min period (see Figure 4 c below). Slow binding interactions have not been observed for any other class of CYP121 ligand (aniline, imidazole and triazole structures) that have been previously studied. These unusual optical properties and the structural similarity of fragment **1 a** to the CYP121 substrates prompted further investigation of the SAR contributing to fragment **1 a‐**CYP121 binding interactions.

### Binding mode and minimal pharmacophore of tryptophan methyl ester

**Figure 2:**
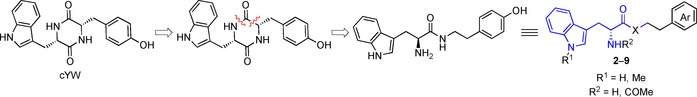
Substrate‐inspired elaboration of tryptophan methyl ester **1 a**. Cleavage of the diketopiperazine ring of cyclo‐l‐Tyr‐l‐Trp (cYW) yields linear compounds **2**–**9** that incorporate the scaffold of fragment **1 a** (blue). Modification of the amide motif (X), α‐amino group (R^2^) and indole nitrogen atom (R^1^) provides insight into key heme binding motif and SAR.

A selection of analogues structurally related to fragment **1 a** were screened by UV/vis spectroscopy in order to identify the heme interacting functional group and to establish the initial SAR contributing to CYP121–**1 a** binding interactions (Table S1, Supporting Information). P450 inhibitors that exhibit type II optical spectra typically interact with the heme cofactor using the lone pair electrons of a nitrogen atom, with which they either directly coordinate to the heme iron or indirectly bind the heme iron by stabilising the axial heme water ligand. As such, fragment **1 a** was predicted to interact with the heme iron using either the α‐amino group or indole nitrogen.^36^, ^37^, ^38^ Screening results from this initial set of fragment analogues indicated that the methyl ester of fragment **1 a** was a key feature of the binding pharmacophore. Replacement of the ester with a range of functional groups, including carboxylic acids, primary (thio)‐amide, alcohol, amine or nitrile substituents disrupted heme binding interactions (Δ*λ*_max_=0 nm). As such, the synthesis of specific analogues that retained an ester substituent was required in order to clarify whether the α‐amine or the indole nitrogen atom of fragment **1 a** was essential for heme binding. The design of these analogues was guided by the size and structure of the substrate cYW, which when linearised by cleavage of the diketopiperazine ring would yield elaborated analogues of fragment **1 a** (Figure 2).

**Scheme 1:**
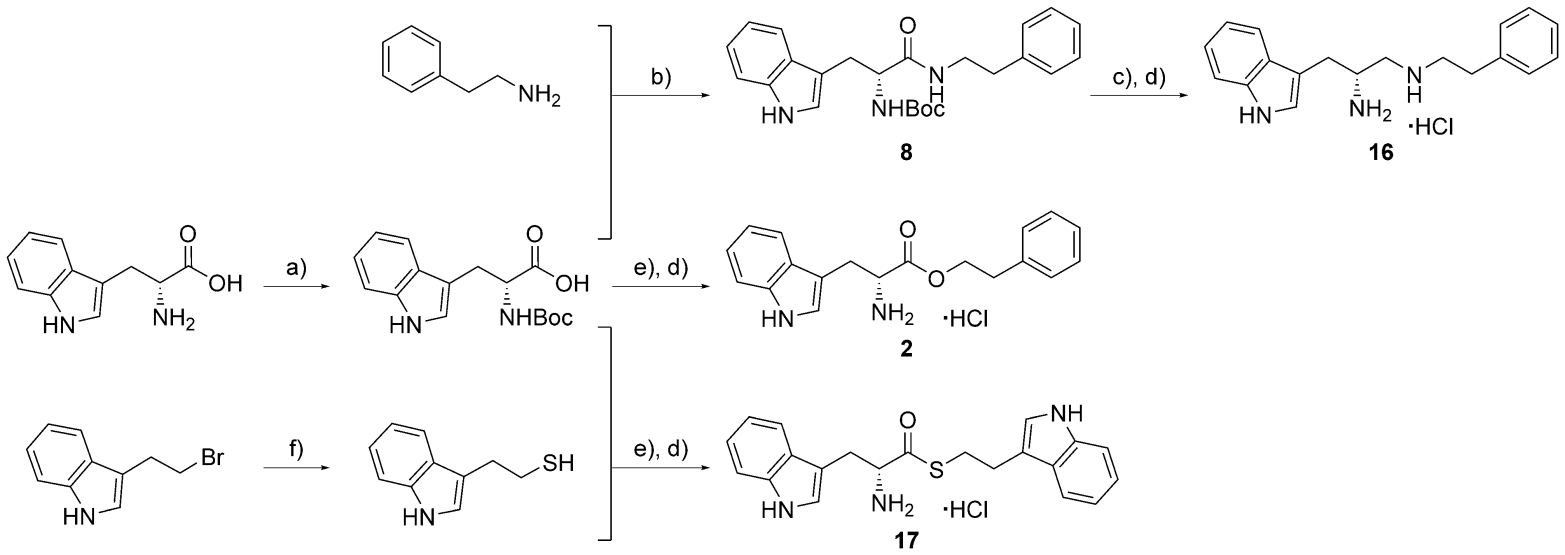
Example synthesis of ester, thioester and amide and amine analogues of fragment 1 a. *Reagents and conditions*: a) BOC‐ON®, Et_3_N, 1,4‐dioxane/water (1:1), RT, 5 h; b) EDC⋅HCl, HOBt, Et_3_N, CH_2_Cl_2_, RT, 24–36 h; c) Red‐Al®, THF/toluene (2:3 *v*/*v*), 0–20 °C, 5 min, then 40 °C, 15 h, then NaOH, 5 °C, 1 h; d) 4 m HCl in 1,4‐dioxane, RT, 1 h; e) DCC, DMAP, CH_2_Cl_2_, 0 °C→RT, overnight; f) thiourea, EtOH, reflux, 24 h, then 2 m NaOH, EtOH, reflux, 4 h.

The phenethyl ester **2**, as well as its l‐enantiomer **3**,^39^ were synthesised as initial compounds, because they represented the simplest structural elaboration of fragment **1 a** (Table 2). In addition, the *N* ^α^‐acetyl protected **4** and 1‐*N*‐methyl protected **5** analogues of **3** were synthesised in order to probe the importance of the nitrogen atoms for heme binding. The general syntheses of these compounds is outlined in Scheme 1, using compound **2** as an example. All other (thio)‐ester and amide compounds in this study were prepared by a similar route (Detailed synthetic schemes and characterisation all compounds can be found in Supporting Information). The free α‐amine of the appropriate amino acid precursor was protected as the *tert*‐butyl carbamate (Boc) derivative and then coupled to the appropriate alcohol, thiol or amine using carbodiimide chemistry. Deprotection of the α‐amino group under anhydrous acidic conditions yielded the desired ligands **2**, **3**, and **5** as their hydrochloride salts, while compound **4** was retained as the *N* ^α^‐acetyl protected ester.

**Table 2:** Structures and binding affinity data for linearised CYP121 substrate analogues and ligands designed to establish the heme binding mode of fragment **1 a**.

Compound			Δ*λ*_max_ [nm]^[a]^			*K*_d_ [μm]^[b]^	LE^[c]^
			1 mm	100 μm			
**1 a**	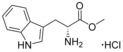		+4.0	–		840±88	0.26
**2**	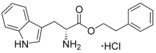		+4.5	+1.0		200±17	0.22
**3** ^[d]^	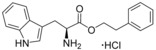		+4.0	+1.0		200±19	0.22
**4**	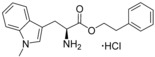		+4.5	+0.5		140±22	0.22
**5**	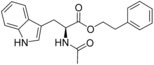		+0.5*	0		ND	ND
**6**	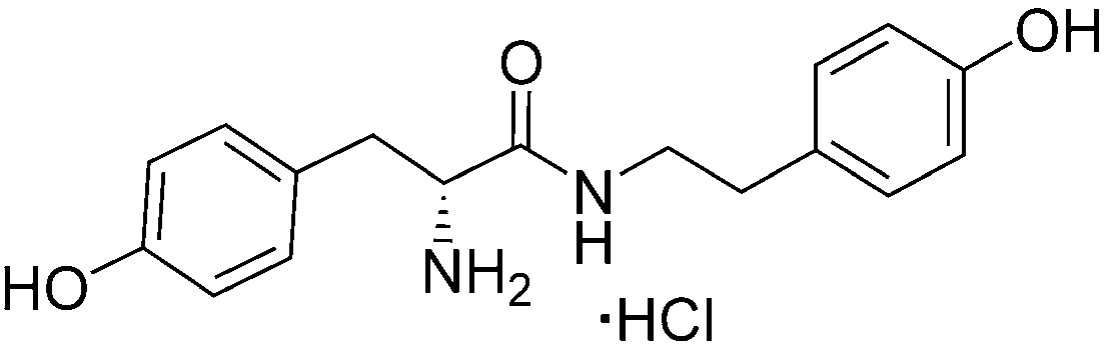		−0.5	−0.5		ND	ND
**7**	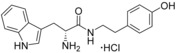		+4.5	+1		260±14	0.20
**8**	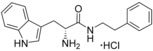		+4.5	+0.5		270±22	0.21
**9**	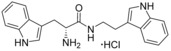		+7	+1		110±6.0	0.21

[a] Magnitude of change in the maximum wavelength of the Soret band of the CYP121 optical spectrum calculated relative to a DMSO (1 % *v*/*v*) control (*λ*_max_=416.5 nm); compounds were screened at 1 mm/100 μm (*except **5**, which was screened at 500 μm/100 μm, owing to limited solubility). [b] Spectral dissociation constant from the optical titration of CYP121 (5 μm) with compounds; data were fitted using a standard hyperbolic (Michaelis–Menten) function as described in the Supporting Information. [c] Ligand efficiency (kcal mol^−1^ per heavy atom). [d] Synthesised as described in ref. 28. ND: not determined.

Both compounds **2** and **3**, and the 1‐*N*‐methylated analogue **5**, produced type II shifts of +4.0–4.5 nm in the Soret *λ*_max_ of the CYP121 optical spectrum. In contrast, the *N* ^α^‐acetylated analogue **3** did not produce a significant change in the Soret (Δ*λ*_max_<1 nm), indicating that the heme binding interactions of fragment **1 a** and analogues were mediated by the α‐amino group. Aliphatic amines are relatively uncommon among reversible P450 inhibitors compared to heteroaromatic nitrogen ligands. The inherently stronger heme binding affinity of heteroaromatic ligands arises from their greater lipophilicity and pi‐acceptor properties. However, alkyl amines are well characterised to form quasi‐irreversible mechanism‐based P450 inhibitors.^34^ These compounds typically exhibit greater isoform selectivity than reversible P450 inhibitors and have previously been incorporated in substrate analogues designed to selectively inhibit steroid biosynthesis.^40^, ^41^, ^42^ Heme co‐ordination by a primary aliphatic amine has not been previously identified for CYP121 ligands and the use of this heme binding group might allow the development of a series of CYP121 inhibitors with good selectivity over other P450 enzymes. To investigate this prospect, fragment **1 a** was screened against a panel of six other *Mtb* P450s and found to produce type II binding interactions with only one other isoform, the cholesterol/cholestenone oxidase CYP142 (see Table 6 below).

The binding affinities of fragment **1 a** and phenethyl ester analogue **2** were determined by optical titration to be 840±88 μm and 200±17 μm, respectively, demonstrating that elaboration from the ester motif of **1 a**, guided by the size and structures of CYP121 substrates produced a significant improvement in binding affinity. Furthermore, elaboration of the ester to include a second aromatic motif overcame the enantioselectivity of CYP121 for fragment **1 a** over its l‐tryptophan methyl ester isomer, which had not been identified as a hit in thermal shift screening and did not perturb the Soret *λ*_max_ of CYP121 in UV/vis assays (Table S1, Supporting Information). In contrast, the l‐phenethyl ester **3** had an equivalent *K*_d_ of 200±19 μm to that of compound **2**.

Encouraged by the 4‐fold improvement in the binding affinity of **2** over **1 a**, a series of linearised substrate analogues were designed, which contained an amide functional group in place of the ester of **2** to more closely mimic the CYP121 substrates. Analogues resembling acyclic versions of cYY‐**6**, cYW‐**7**, and two hypothetical cyclo‐dipeptides “cWF”‐**8** and “cWW”‐**9** were synthesised according to the route outlined in Scheme 1. Compound **6** did not show binding interactions with the heme cofactor (Δ*λ*_max_∼0 nm) in direct UV/vis experiments or competitive binding interactions when tested in combination with a known type II CYP121 inhibitor clotrimazole (Δ*λ*_max_=+8 nm, 50 μm; Table 2 and Figure S1, Supporting Information). These experiments indicate that **6** is unlikely to bind within the CYP121 active site, and are consistent with the preference observed for CYP121 to bind tryptophan/indole fragments over phenol or phenyl structures. Compounds **7**–**9** all produced type II shifts in the Soret *λ*_max_ and were calculated to have binding affinities of **7**: 260±14 μm, **8**: 270±22 μm and **9**: 110±6.0 μm.

### Ester bioisostere replacements

**Scheme 2:**
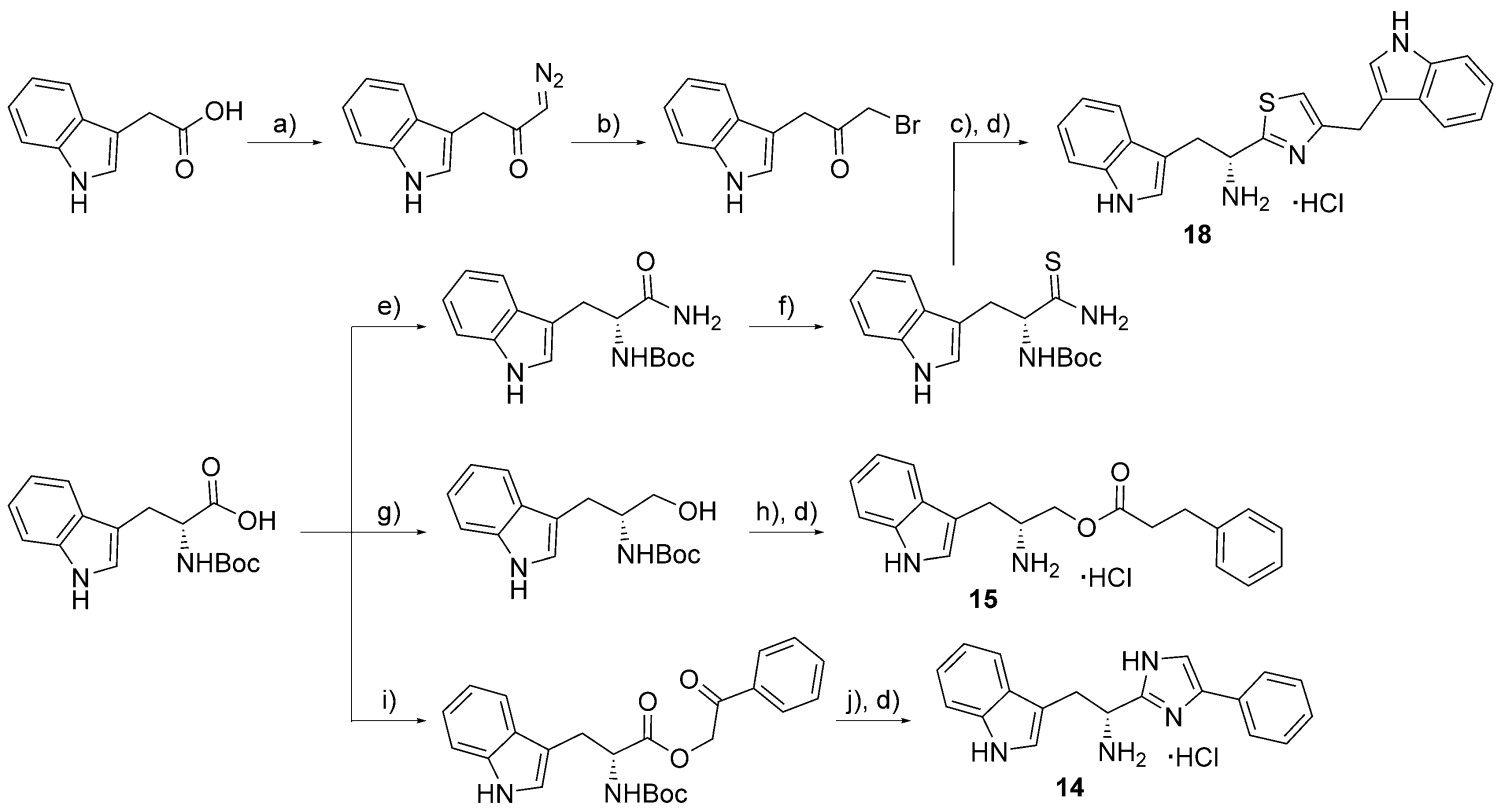
Example syntheses of thiazole, imidazole and homologated ester compounds. *Reagents and conditions*: a) SOCl_2_, DMF, THF, 0 °C, 4 h, then TMS‐diazomethane, THF/MeCN, 0 °C, 4 h; b) HBr_(aq)_ (48 %), AcOH, 0 °C, 40 min; c) EtOH, RT, overnight; d) 4 m HCl in 1,4‐dioxane, RT, 1 h; e) *i*BuCO_2_Cl, NMO, DME, NH_3(aq)_ (35 %), 0 °C→RT, 2 h; f) NaHCO_3_, P_2_S_5_, DME, RT, overnight, or Lawesson′s reagent, THF, RT, overnight; g) 1. *i*BuOCOCl, NMM, DME, −15 °C, 2 min; 2. NaBH_4_, H_2_O, −15 °C, 5 min; h) 3‐phenylpropionic acid, DCC, DMAP, CH_2_Cl_2_, 0 °C→RT, overnight; i) 1. Cs_2_CO_3_, EtOH, RT, 30 min, 2. PhCOCH_2_Br, DMF, RT, 4 h; j) NH_4_OAc, *m*‐xylene, reflux, 1 h.

The difference in the binding affinity of ester **2** and amide **8** indicated that the ester group was an important contributor to CYP121–ligand interactions. Furthermore, it was noted that only substrate fragments that contained an ester functional group were identified as type II ligands when screened by UV/vis spectroscopy (Table 1 and Table S1, Supporting Information). To investigate the SAR of the ester group, a series of compounds containing various bioisosteres were synthesised (Table 3). All of these compounds contained phenyl or phenethyl substituents to allow access to a wider variety of synthetic precursors and to enable the binding contribution of the bioisosteric group to be assessed independently of other structure changes. Ester, thioester and amide containing compounds were synthesised as described earlier for compounds **2** and **8** in Scheme 1. Thiol precursors that were not commercially available, such as those required for the synthesis of compounds **17**, **23**, **23** and **25** (Table 4), were prepared from the respective alkyl bromides, using thiourea followed by hydrolysis with sodium hydroxide (Scheme 1). Thiazoles were prepared from the condensation of Boc‐protected primary thioamide intermediates with α‐bromoketones, as described for the synthesis of compound **18** (Scheme 2). Primary thioamides were synthesised from their respective Boc‐protected amino acid precursors via their primary amides, by substitution of the activated anhydride intermediates with aqueous ammonia, followed by treatment with Lawesson′s reagent or P_2_S_5_ (Scheme 2). α‐Bromoketones that were not commercially available, were obtained by the homologation of the appropriate carboxylic acid precursor with TMS‐diazomethane and then substitution of the diazo group using hydrobromic acid. Imidazole **14** was synthesised from the condensation of Boc‐d‐tryptophan with a 2‐bromoacetophenone, followed by cyclisation of the resulting keto ester intermediate using ammonium acetate in xylene at reflux. “Reverse” ester **15** was obtained from the reduction of Boc‐d‐tryptophan using NaBH_4_, followed by Steglich esterification with 3‐phenylpropionic acid. Amine **16** was synthesised by the reduction of amide **8** using Red‐Al® (sodium bis(2‐methoxyethoxy)aluminium hydride; Scheme 1). All final compounds, and a selection of intermediates, were deprotected under anhydrous acidic conditions to yield the desired hydrochloride salts.

**Table 3:** Structures and binding affinity data of selected analogues that contain bioisosteric replacements for the ester group of compound **2**.

Compound			Δ*λ*_max_ [nm]^[a]^			*K*_d_ [μm]^[b]^	LE^[c]^
			1 mm	100 μm			
**10**	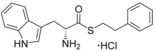		–	+2.5		66±9.7	0.25
**11**	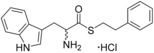		–	+3.5		61±7.6	0.25
**12**	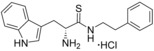		–	+1.5		150±18	0.23
**13**	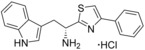		–	+2		44±3.6	0.26
**14**	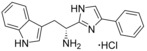		–	+0.5		530±100	0.19
**15**	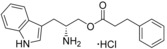		+1.5	+0.5		ND	ND
**16**	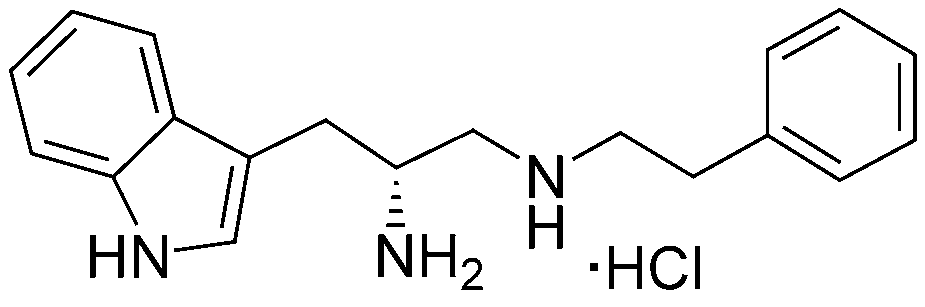		0*	–		ND	ND

[a] Magnitude of change in the maximum wavelength of the Soret band of the CYP121 optical spectrum calculated relative to a DMSO (1 % *v*/*v*) control (*λ*_max_=416.5 nm); compounds were screened at 1 mm/100 μm (*except **16**, which was screened at 500 μm, owing to limited solubility). [b] Spectral dissociation constant from the optical titration of CYP121 (5 μm) with compounds; data were fitted using a standard hyperbolic (Michaelis–Menten) function as described in the Supporting Information. [c] Ligand efficiency (kcal mol^−1^ per heavy atom). ND: not determined.

**Table 4:** Structures and binding affinity data of tryptophan thioester and thiazole compounds substituted with a range of aromatic groups.

Compound		Δ*λ*_max_ [nm]^[a]^	*K*_d_ [μm]^[b]^	LE^[c]^
**17**	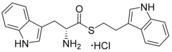	+1.5	45±4.4	0.23
**18**	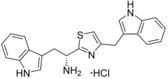	+1.0	52±3.1	0.22
**19**	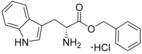	0	ND	ND
**20**	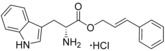	+1.5	120±21	0.22
**21**	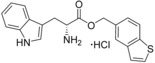	+2.0	91±31	0.22
**22**	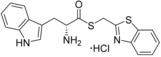	+0.5	ND	ND
**23**	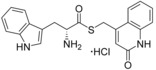	+0.5	ND	ND
**24**	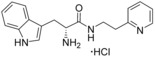	+0.5	ND	ND
**25**	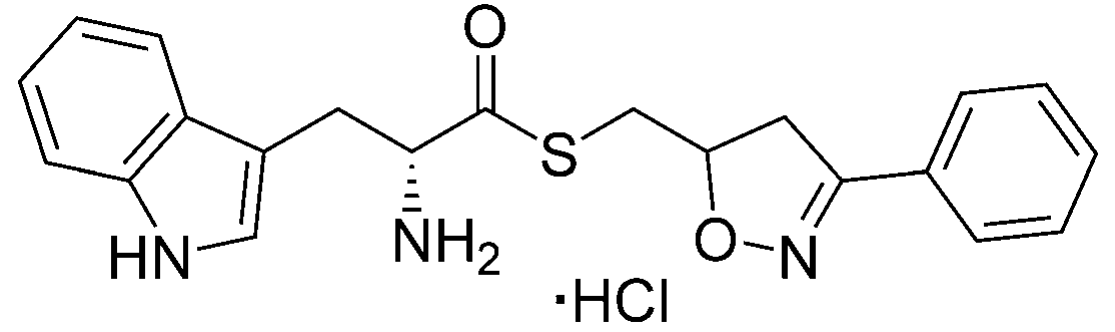	+3.0	35±4.2	0.22

[a] Magnitude of change in the maximum wavelength of the Soret band of the CYP121 optical spectrum calculated relative to a DMSO (1 % *v*/*v*) control (*λ*_max_=416.5 nm); compounds were screened at 100 μm. [b] Spectral dissociation constant from the optical titration of CYP121 (5 μm) with compounds; data were fitted using a standard hyperbolic (Michaelis–Menten) function as described in the Supporting Information. [c] Ligand efficiency (kcal mol^−1^ per heavy atom). ND: not determined.

The compounds were analysed for binding to CYP121 by UV/vis spectroscopy and dissociation constants (*K*_d_ values) were determined by optical titration for select compounds (Table 3). The rigidity, polarity and position of the ester (or equivalent functional group) relative to the α‐amine, were each shown to be important for CYP121 binding affinity. Homologation and reversal of the ester motif **15**, or reduction of the replacement of the ester with an amine **16**, was detrimental, with neither compound **15** nor **16** producing significant perturbation in the Soret *λ*_max_. Conversion of amide **8** to thioamide **12** resulted in a twofold improvement in binding affinity, which we hypothesised might be a conformational effect resulting from the thioamide favouring an imine resonance structure.^43^ The further threefold improvement in binding affinity that resulted from constraining the thio‐imine into a thiazole ring, as in compound **13**, provided support for this conclusion. Conversion of ester **2** to thioester **10** also resulted in a threefold improvement in binding affinity. Ligand docking simulations predicted that the ester motif of **2** likely formed interactions with an arginine residue (Arg386) that is positioned in close proximity to the CYP121 heme cofactor (see Figure 5 below). Arg386 has previously been identified to form a water‐bridged hydrogen bonding network with either the phenol −OH or diketopiperazine carbonyl group of the CYP121 substrates cYY and cYW, respectively. These interactions have been proposed to both stabilise the binding of the substrates and to be essential for the CYP121 catalytic mechanism.^31^, ^33^ The introduction of sulfur‐containing (thioester, thiazole, and thioamide) bioisosteres produced a consistent improvement in the binding affinity of all compounds that were subsequently synthesised, relative to their respective ester or amide counterparts. These SAR are likely the result of both the different conformational and electronic properties imparted by the sulfur substitution. A significant loss in binding affinity was observed when the thiazole ring of **13** was replaced with imidazole **14**. This might be a consequence of mixed binding modes, as both the imidazole and α‐amino groups of **14** could form favourable interactions with the heme cofactor.

The racemic analogue **11** of thioester **10** was also synthesised and found to have a similar *K*_d_ value of 61±7.6 μm, confirming the results obtained for the d‐ and l‐phenethyl ester analogues **2** and **3**, respectively. Ligand docking simulations were used to justify these results, demonstrating that both the d‐ and l‐enantiomers of compounds that contained two aromatic motifs could satisfy the CYP121 binding pharmacophore (Figure 5 b,c). For example, both enantiomers should be able to form interactions with the heme cofactor and Arg386, as well as aromatic stacking interactions with Phe168 and Trp182 residues. These aromatic interactions have been previously identified as a binding hotspot in the CYP121 active site and are conserved in the X‐ray crystal structures of CYP121 substrates.^25^, ^33^ Confident that stereochemistry did not affect the binding affinity of the ligands, a range of racemic amino acid derivatives were employed during subsequent iterations of fragment optimisation in order to explore the binding contribution of the tryptophan indole.

### Aromatic group optimisation

The SAR of the phenylethyl substituent and indole ring of the ligands were subsequently investigated (Tables 4 and 5). Thioester **10**/**11** and thiazole **13** were selected as structural templates for this investigation, as they had provided the greatest improvement in CYP121 binding affinity relative to the ester group of fragment **1 a** and compound **2**. However, a selection of ester and amide compounds were also synthesised because of availability of a wider variety of synthetic precursors.

**Table 5:** Structures and binding affinity data of phenethyl‐thioester and phenyl‐thiazole compounds substituted with a range of aromatic amino acids.

Compound		Δ*λ*_max_ [nm]^[a]^	*K*_d_ [μm]^[b]^	LE^[c]^
**26**	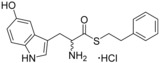	+1.5	130±9.3	0.22
**27**	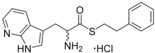	+1.5	110±9.1	0.23
**28**	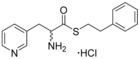	0	ND	ND
**29**	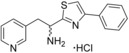	+0.5	ND	ND
**30**	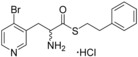	+3.5	30±3.8	0.29
**31**	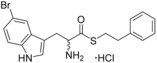	+5.5	16±2.4	0.27
**32**	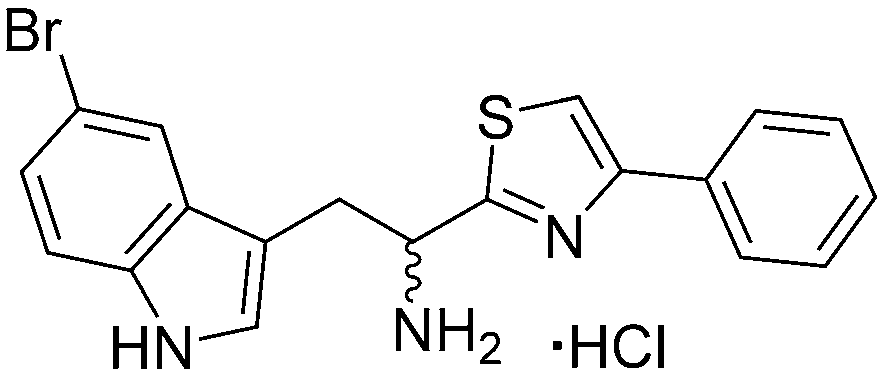	+3	32±2.5	0.26

[a] Magnitude of change in the maximum wavelength of the Soret band of the CYP121 optical spectrum calculated relative to a DMSO (1 % *v*/*v*) control (*λ*_max_=416.5 nm); compounds screened at 100 μm. [b] Spectral dissociation constant from the optical titration of CYP121 (5 μm) with compounds; data were fitted using a standard hyperbolic (Michaelis–Menten) function as described in the Supporting Information. [c] Ligand efficiency (kcal mol^−1^ per heavy atom). ND: not determined.

It was hypothesised that substitution of the phenylethyl group with an indole or other heteroaromatic group might produce a similar improvement in the binding affinity of scaffolds **10** and **13** as had been observed between the “cWY”‐**7**/“cWF”‐**8** analogues and the acyclic “cWW” compound **9**. However, whereas indole‐substituted thioester **17** and thiazole **18** maintained binding affinities similar to those of **10** and **13**, they provided no further improvement. Decreasing the chain length of the phenyl ester substituent **19** was detrimental to binding affinity, while increasing the length or rigidity of a hydrophobic substituent by the introduction of a *trans*‐alkene **20** or fused aromatic ring **21** produced a twofold improvement in affinity relative to ester **2**. The introduction of a range of aromatic substituents that contained polar heteroatoms, such as in compounds **22**, **23**, and **24**, was not tolerated and only the phenyl‐isoxazole‐substituted compound **25** produced an improvement in binding affinity (*K*_d_=35 μm) from this series of compounds. Polar heteroatoms in close proximity to the (thio)‐ester group were postulated to interfere with Arg386 interactions, while introducing shorter (thio)‐ester substituents might prevent compounds from accessing binding interactions with active site residues. The relatively high ligand selectivity observed for CYP121 is characteristic of biosynthetic P450s and demonstrates the important contribution of active site interactions to the binding affinity of this series of ligands.^34^

**Figure 3:**
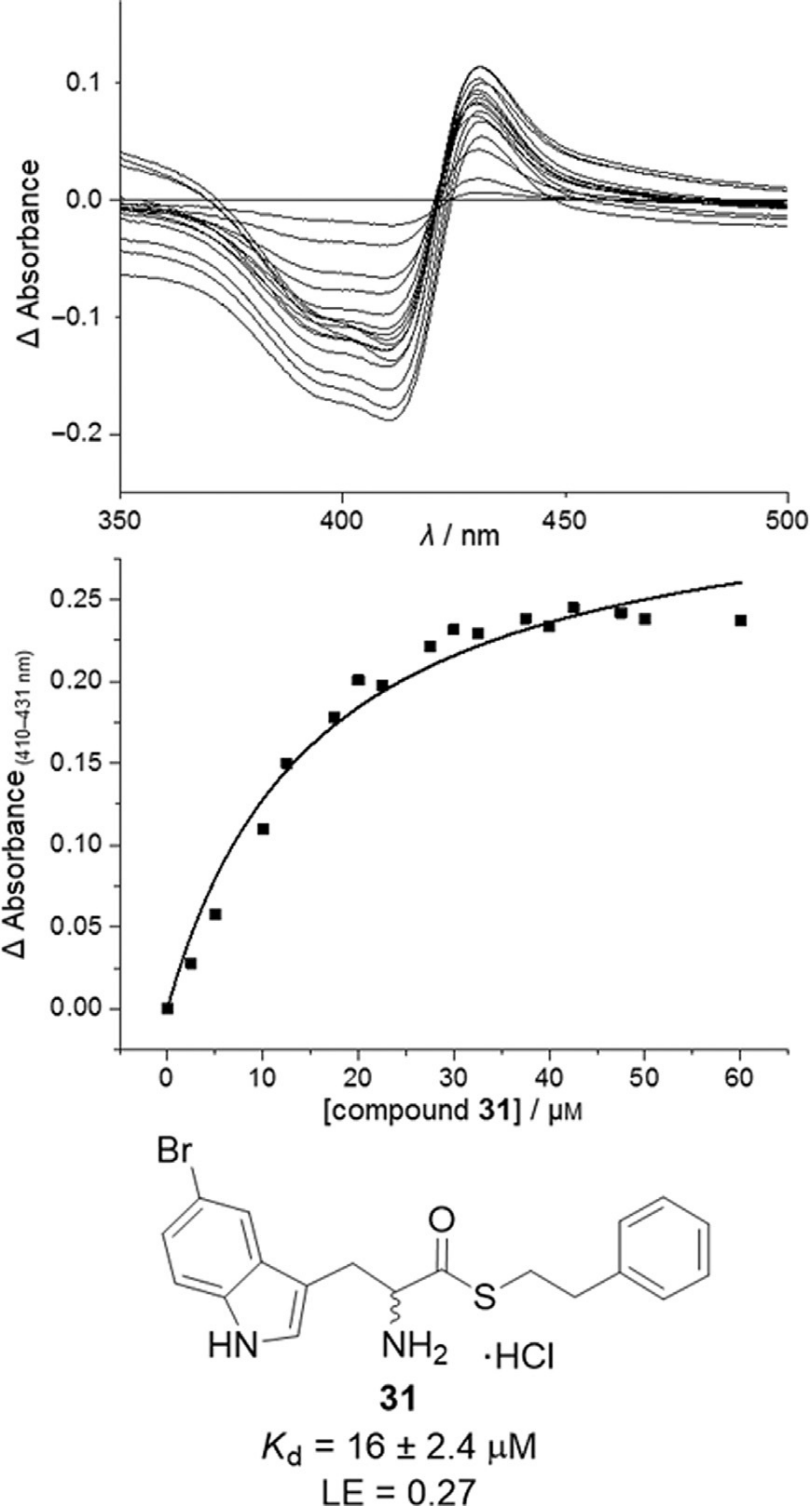
Optical difference spectra (top) and ligand‐induced change in Soret absorbance (bottom) for the titration of CYP121 with compound **31**. Data were fitted using a standard hyperbolic (Michaelis–Menten) function to calculate a *K*_d_ value of 16±2.4 μm and ligand efficiency (LE) of 0.27.

A range of substituted tryptophan, phenylalanine, pyridine or other aromatic amino acid analogues were selected to replace the indole ring of thioester **10**/**11** or thiazole **13** (Table 5). While substitution at C5 of the indole ring was tolerated, the introduction of a polar hydroxy group resulted in a twofold increase in the *K*_d_ value of **26** (*K*_d_=130 μm) relative to **11**. Similarly, the introduction of a second aromatic N atom into the indole ring to give the aza‐tryptophan analogue **27**, produced a similar loss of binding affinity (*K*_d_ (**27**)=110 μm). Replacement of the tryptophan indole with phenol or pyridine heterocycle was detrimental for ligand binding and neither the pyridine thioester **28** nor thiazole **29** compounds produced a change in the Soret *λ*_max_. This was consistent with observations previously noted for compound **6**, the acyclic analogue of cYY (Table 2). In contrast, analogues substituted with carboaromatic rings or halogens, such as 2‐bromophenylanine derivative **30**, improved binding affinity (*K*_d_ (**30**)=30 μm) relative to compound **11**. The addition of bromine at C5 of the tryptophan indole ring similarly improved the binding affinity of both the phenethyl‐thioester **31** and phenyl‐thiazole **32** series of compounds. This modification resulted in the most potent compound **31** to be developed from fragment **1 a**, which was calculated to have a *K*_d_ value of 16 μm and an improved LE over the original fragment (Figure 3).

### Spectroscopic characterisation of inhibitor binding mode and selectivity

The red shift in the Soret *λ*_max_ of the CYP121 optical spectrum that was observed for the enzyme bound to fragment **1 a**, or its elaborated derivatives, is indicative of ligands that interact with the ferric heme iron of a P450 and form a more strongly coordinated complex than the water‐bound resting state.^34^ The magnitude of the Δ*λ*_max_ of the Soret band, which was centred at 420–422 nm for the majority of compounds, is small relative to that expected for heteroaromatic N−Fe^3+^ heme binding groups (*λ*_max_≈424 nm)^36^ and could indicate an indirect interaction between the tryptophan compounds and heme via a bridging axial water molecule, as has been observed in the X‐ray crystal structure of CYP121 in complex with fluconazole (PDB ID: 2IJ7).^44^ However, a recently characterised series of ligands that directly coordinate to the heme iron of CYP121 using a primary aniline as the nitrogen ligand (PDB ID: 5IBE) also produce relatively small shifts in the Soret (*λ*_max_=422 nm at saturating ligand concentrations), despite their having low nanomolar affinity.^25^

**Figure 4:**
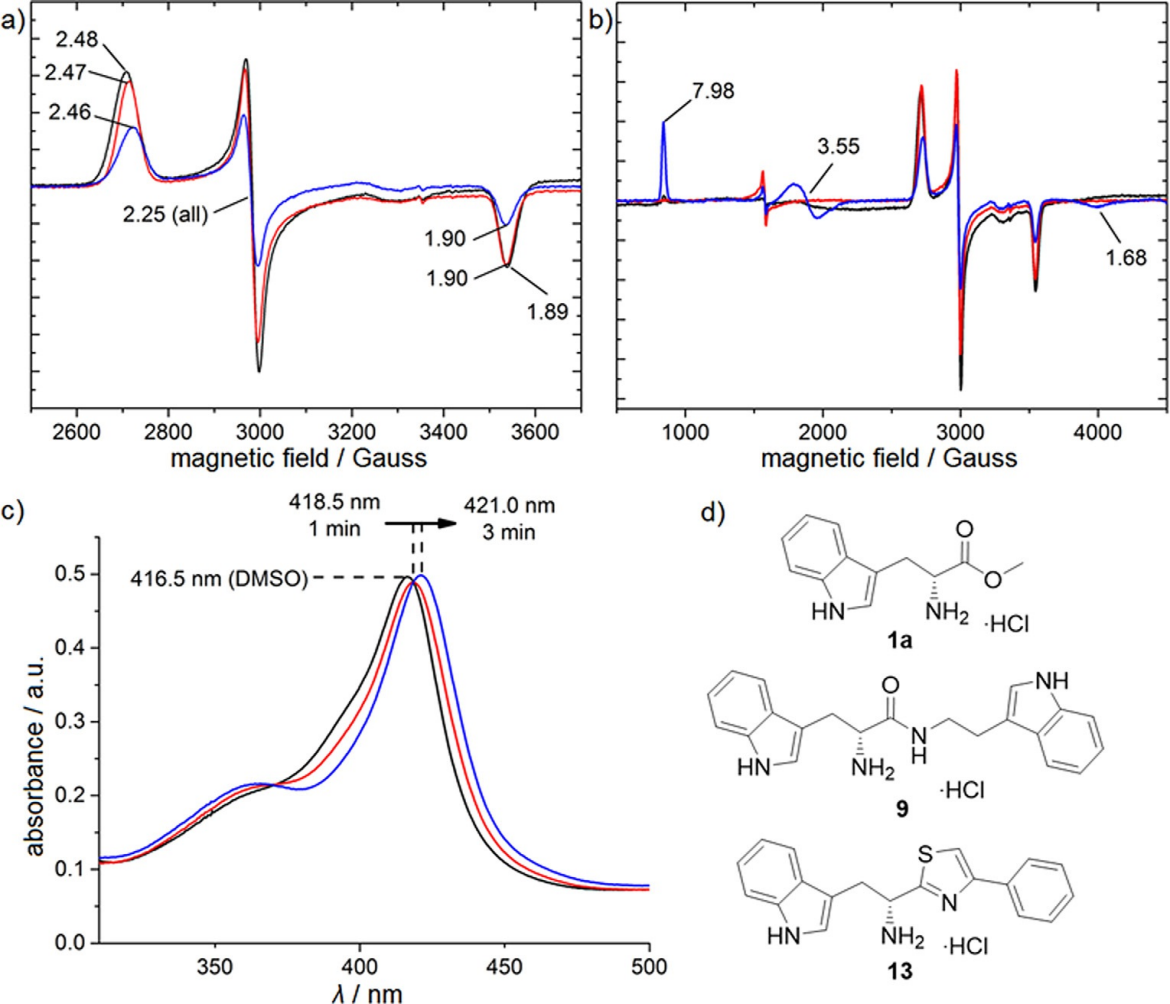
Spectroscopic characterisation of CYP121–ligand binding interactions. a) X‐band EPR spectra collected over a narrow scan (2000–4000 G) and b) a wide scan (500–4500 G), for ligand‐free CYP121 (100 μm, black) and CYP121 (100 μm) bound to compound **13** (2 mm, red) or compound **9** (2 mm, blue). The low‐spin *g* values generated are annotated for all samples in panel (a). In panel (b), the high‐spin *g* values generated by compound **9** are annotated. c) The optical spectrum of ligand‐free CYP121 (5 μm, black) and CYP121 bound to fragment **1 a** (2 mm) collected after 1 min (red) or 3 min (blue) incubation. The Soret *λ*_max_ value is labelled for each spectrum. d) Structures of compounds **1 a**, **9** and **13**.

Electron paramagnetic resonance (EPR) spectra of CYP121 in complex with fragment **1 a** and fourteen of the elaborated compounds, were collected to gain further insight into their binding interactions (Table 3, Supporting Information). All compounds generated a rhombic triplet of *g* values that are consistent with a low spin, cysteinate‐coordinated ferric P450 (Figure 4 a).^36^ The change in the *g* values of the ligand‐free CYP121 protein (*g*_z_=2.48, *g*_y_=2.25, *g*_x_=1.89) that were observed for inhibitor‐bound CYP121 complexes (e.g., compound **31**, *g*_z_=2.46, *g*_y_=2.25, *g*_x_=1.90) were similar to those previously reported for both CYP121–fluconazole (*g*_z_=2.45, *g*_y_=2.26, *g*_x_=1.90)^44^ and CYP121–cYY (*g*_z_=2.46, *g*_y_=2.25, *g*_x_=1.89/1.90)^31^ complexes, both of which retain an axial heme water ligand in X‐ray co‐crystal structures. However, the previously noted series of CYP121 aniline ligands that displace the axial water ligand, also generate similar *g* values of 2.44/2.24/1.90 (Table 4 and Figure S2, Supporting Information). The direct coordination of heteroaromatic nitrogen ligands to ferric P450 heme iron is well characterised in the literature and is expected to cause a widening of the low‐spin *g* values (e.g., *g*_z_∼2.50–2.65), as is observed for CYP121 bound to econazole (*g*_z_=2.48, *g*_y_=2.24, *g*_x_=1.90).^36^ The opposite trend in the *g* values of EPR spectra collected for CYP121 in complex with the current series of tryptophan (aliphatic amine) ligands, and the previous series of aniline ligands, in addition to their characteristic type II optical spectra and crystallographic evidence for direct aniline‐Fe^3+^ binding interactions,^25^ provides novel insight into the spectroscopic effect of weak donor ligands on the P450 heme environment.

The majority of the tryptophan compounds stabilised the low‐spin state of CYP121, decreasing the small amount (0.9–3.1 %) of high‐spin enzyme that is present in X‐band EPR spectra collected for the water‐ligated, resting state enzyme. One exception was the acyclic “cWW” analogue **9**, which increased the proportion of high‐spin CYP121 to ∼34 % (Figure 4 b, and Table S3, Supporting Information), significantly more than that generated by cYY in complex with CYP121 (∼2.2 % high‐spin; Figures S2 and S4, Supporting Information). The small proportion of high‐spin enzyme observed for CYP121 in complex with cYY is likely a consequence of the low temperatures (∼10 K) required to collect P450 X‐band EPR spectra. The greater retention of high‐spin CYP121 generated by compound **9** suggests that the ligand forms more intimate contacts with the distal face of heme group, possibly through indole‐heme stacking interactions, as observed in the X‐ray crystal structure of cYW (PDB ID: 4IQ9).^33^ These stronger interactions are reflective of the preferential binding of CYP121 to fragment **1 a**/indole derivatives over the respective tyrosine/phenol analogues that was revealed fragment screening by thermal shift and UV/vis spectroscopy. The generation of high‐spin CYP121 by compound **9**, in addition to the low micromolar *K*_d_ values determined for all three of the di‐indole compounds **9**, **17**, and **18**, which are on the same order of magnitude as the *K*_d_ value of CYP121 for cYY,^33^ suggests a preference of CYP121 for tryptophan dipeptides. While the *Mtb* cyclic dipeptide synthetase Rv2275 was not found to synthesise di‐Trp (cWW) peptides when expressed in *E. coli*,^32^ cW‐X natural products are prevalent in other microbial species, particularly fungi, and have potent biological activities.^45^ Although catalytic turnover of compound **9** was not observed when CYP121 activity was reconstituted with NADPH and ferredoxin/ferredoxin reductase redox partners,^31^ the preference of CYP121 for the di‐indole structural motif could indicate a broader substrate profile to that previously identified or a possible role for CYP121 in microbial defence mechanisms.

Spectroscopic evidence that the tryptophan compounds form relatively weak heme binding interactions suggests that they instead obtain a significant proportion of their binding affinity from interactions made with enzyme active site residues. This might engender the compounds with an improved selectivity profile over inhibitors which have metal‐coordination driven modes of binding. In support of this conclusion, lead compound **31** and cWW analogue **9** were both found to have good selectivity for CYP121 over a panel of six other *Mtb* P450s, when they were analysed by UV/vis spectroscopy. Type II binding interactions were not identified for either **31** or **9** with any of the P450s other than CYP121. However, a weak blue shift was observed in the spectrum of the cholesterol and fatty acid oxidase CYP124 (Table 6).

**Table 6:** Heme binding selectivity of fragment **1 a**, di‐indole “cWW” analogue **9** and optimised compound **31**.^[a]^

Compd	CYP121	CYP124	CYP125	CYP126	CYP142	CYP143	CYP144
**1 a**	+4.5	0	−0.5	+0.5	*+2*	0	+0.5
**9**	+2.0	−2	0	+0.5	0	−0.5	+0.5
**31**	+5.5	−2	0	0	0	0	+0.5

[a] The change in the maximum wavelength of the Soret band (Δ*λ*_max_, nm) of the optical spectrum of each enzyme in the presence of compounds (**1**: 2 mm, **9**: 100 μm or **31**: 10 μm) relative to DMSO (1 % *v*/*v*) alone is shown. Ligand concentrations were selected based on compound solubility. Type II and type I binding interactions are indicated by a decrease or increase in the Soret *λ*_max_, respectively. Changes in the (*λ*_max_<1 nm) were considered within experimental error.

**Figure 5:**
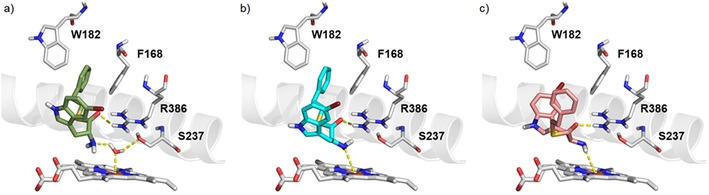
Proposed binding mode of lead compound **31** to CYP121. The d‐ and l‐enantiomers of compound **31** were docked into the prepared X‐ray crystal structure of CYP121 in complex with cYW (PDB ID: 4IQ9).^33^ a) Proposed water‐bridged heme binding mode of d‐**31** (green); b) direct heme binding mode of d‐**31** (cyan); c) direct heme binding mode of l‐**31** (salmon). Proposed hydrogen bonding interactions with active site residues (grey) and metal coordination to the heme cofactor (grey) are shown as yellow dashes. The I helix of the CYP121 protein backbone is shown as a grey helix for orientation. Docking was performed using Glide ver. 6.5 (Schrödinger LLC, NY, 2014‐4), and the figures were prepared using the PyMOL Molecular Graphics System ver. 1.3.

Spectroscopic characterisation of CYP121–ligand binding interactions and the SAR established during fragment elaboration allowed the binding mode of analogues to be modelled in ligand docking simulations (Figure 5). Compounds were predicted to either coordinate directly to the heme iron via the α‐amino group (Figure 5 b), or indirectly co‐ordinate via hydrogen bonding interactions to the axial water ligand (Figure 5 a). Possible progression between these two binding modes, that is, the formation of a hydrogen bridge complex initially, followed by displacement of the axial water to form direct N−Fe^3+^ coordination, might explain the slow onset of spectral perturbations observed for these ligands (Figure 4 c).

## Conclusions

The deconstruction of CYP121 substrates into their component fragments has been used to identify structural features of the larger compounds that can be used to drive binding affinity. Substrate fragments were preferential identified to increase the denaturation temperature of the enzyme when screened as part of a library of chemically similar fragments. Validation of the fragment hits using UV/vis spectroscopy resulted in the identification of a substrate fragment that exhibited an unexpected inhibitor‐like mode of binding using a previously unreported aliphatic amino group. Combining this inhibitory pharmacophore with the structures of CYP121 substrates allowed rational synthetic elaboration to improve fragment binding affinity. The SAR established during synthetic optimisation support the importance of specific binding hotspots and the role of certain active site residues for ligand recognition. These CYP121 inhibitors represent a chemically distinct class of compounds to those previously reported and interact via a different mechanism. The compounds have low micromolar affinity, apparent selectivity for CYP121 over other *Mtb* P450s and are predicted to have good physicochemical properties.^46^ Spectroscopic characterisation of the binding interactions of CYP121 with these compounds revealed that weak nitrogen donor ligands can have unusual effects on the heme microenvironment and greater analysis of these interactions is warranted. A preference of CYP121 for di‐indole dipeptide mimetics was revealed, provoking speculation on the biological role of the enzyme. This study demonstrates the use of fragments to deconvolute the binding interactions of large molecules, identify novel binding modes and chemical scaffolds, and to understand factors contributing to enzyme‐ligand recognition.

### Additional data

Additional data relating to this publication are available at the University of Cambridge data repository: https://www.repository.cam.ac.uk/handle/1810/256410.

## Supporting information

As a service to our authors and readers, this journal provides supporting information supplied by the authors. Such materials are peer reviewed and may be re‐organized for online delivery, but are not copy‐edited or typeset. Technical support issues arising from supporting information (other than missing files) should be addressed to the authors.

Supplementary
