# Supplementary material for: Substrate Fragmentation for the Design of M. tuberculosis CYP121 Inhibitors
Source: ChemMedChem. 2016 Jul 19;11(17):1924–35. doi: 10.1002/cmdc.201600248 (PMC5026067; doi:10.1002/cmdc.201600248)
Supplement: Supplementary file 1 — Supplementary [file CMDC-11-1924-s001.pdf]

CHEMMEDCHEM
Chemistry & Drug Discovery

Supporting Information

Substrate Fragmentation for the Design of *M. tuberculosis* CYP121 Inhibitors

Madeline E. Kavanagh,[a] Janine L. Gray,[a] Sophie H. Gilbert,[a] Anthony G. Coyne,[a] Kirsty J. McLean,[b] Holly J. Davis,[a] Andrew W. Munro,[b] and Chris Abell*[a]

cmdc_201600248_sm_miscellaneous_information.pdf

Supporting Information

Page S2	**Table S1.** Structures and screening data for select substrate fragments and structurally related analogues.
Page S5	**Figure S1.** UV-vis absorbance spectra of CYP121 and compound **6** in competition with the type II ligand clotrimazole.
Page S6	**Table S3.** The g-values and proportion of high spin enzyme generated by ligands binding to CYP121 in X-band EPR spectroscopy experiments.
Page S7	**Figure S2**. EPR spectra of ligand-free CYP121, and CYP121 bound to cYY (blue) or aniline ligand **25a**.
Page S8	**Table S4.** The g-values and proportion of high spin enzyme generated by CYP121 binding to cYY, fluconazole, econazole and compound **25a.**
Page S9	Supplementary synthetic schemes – **Schemes S1-S6**
Page S12	Synthesis
Page S13	General synthetic procedures A-G
Page S14	Compound characterisation
Page S48	Molecular biology, biophysical and biochemical methods

Table S1. Structures and screening data select fragments that were deconstructed from CYP121 substrates, and structurally related fragments that were screened to establish the binding mode and minimal pharmacophore of fragment hit **1a**.

**Compound**	**ΔT_m (°C)[a]**	**$\Delta\lambda_{max}$ (nm)[b]**
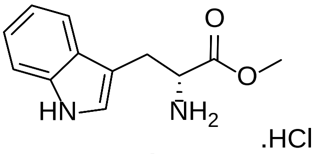 **1a**	+4.5[c]	+1, (+4.5, 2 mM)
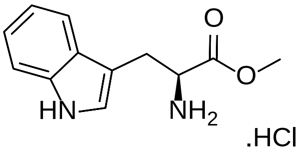	-1.5	0
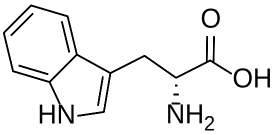	ND	0
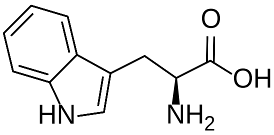	-1.5	ND
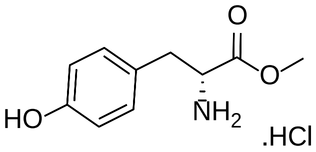	+3[c]	0
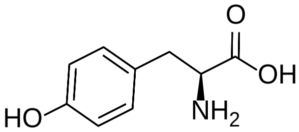	-0.5	ND
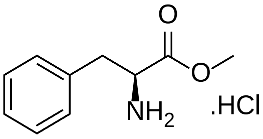	+1.5[c]	ND
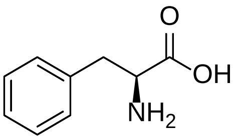	0	ND
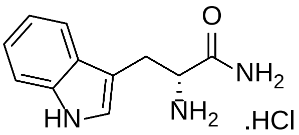	+2.5[c]	0*

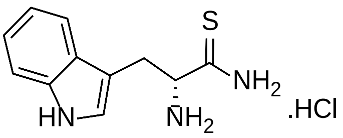	ND	0
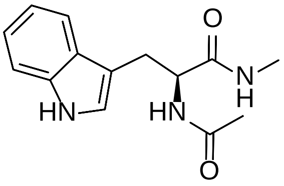	-1	0
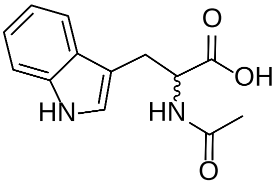	0	ND
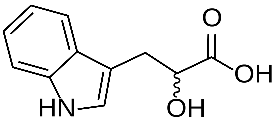	0	0
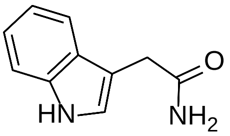	ND	0
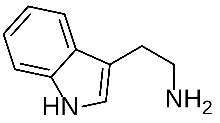	+1.5[c]	0
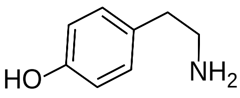	0	ND
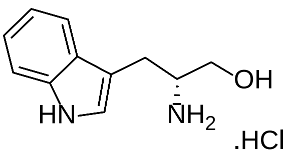	ND	0*
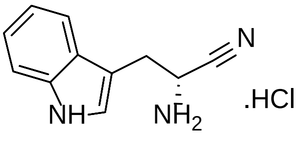	ND	0*
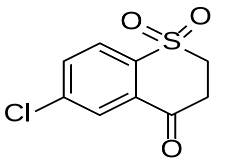	+1.0	0
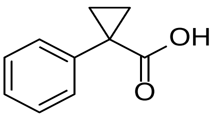	+1.0	0

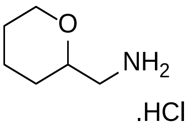	+1.0	0
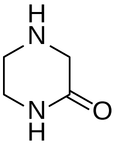	0	ND
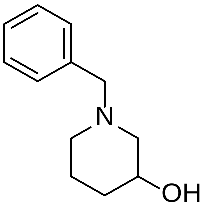	-0.5	ND

[a] Change in the denaturation temperature (T_m) of CYP121 relative to a DMSO (5% v/v) control (T_m = 49.0 °C). Fragments were screened at 5 mM against 5 µM CYP121. [b] Magnitude of change in the maximum wavelength of the Soret band of the CYP121 (5 µM) optical spectrum, calculated relative to a DMSO (1% v/v) control ($\Delta\lambda_{max}$ = 416.5 nm). The fragments were screened at 2 mM. [c] Fragments that are also reported in Table 1 of the main paper.

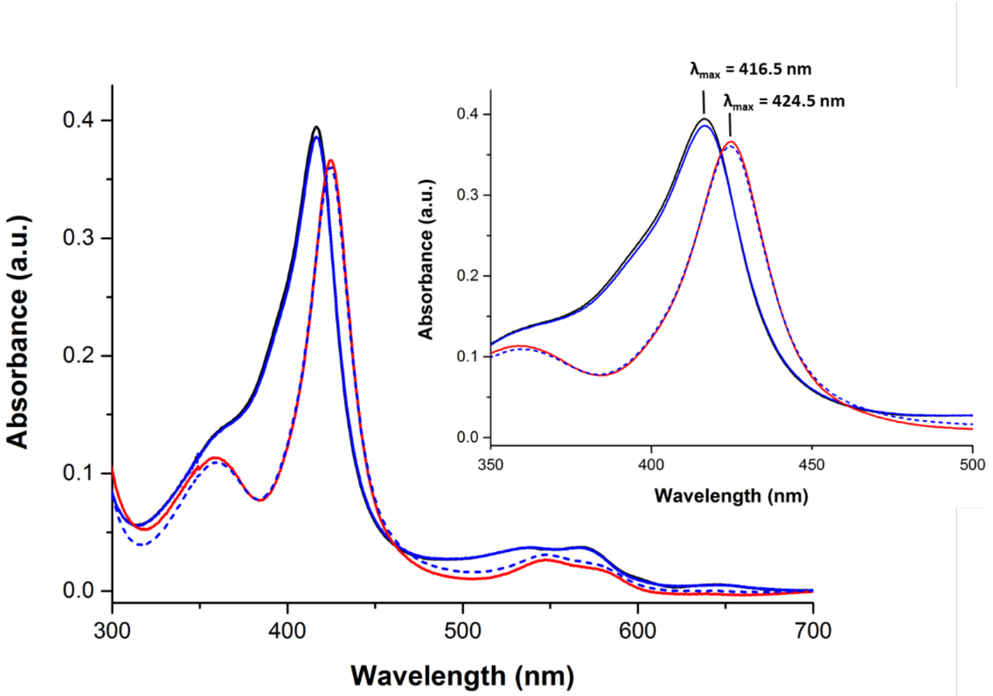

Figure S1. UV-vis absorbance spectrum of CYP121 and CYP121 in the presence of type II ligand clotrimazole or non-binding compound **6**. Ligand-free CYP121 (5 µM) in the presence of 1% v/v d_6-DMSO (black line) has a Soret λ_{max} at 416.5 nm. In the presence of clotrimazole (50 µM) (red line) or compound **6** (500 µM) (blue line) the λ_{max} of CYP121 (5 µM) was at 424.5 nm and 416.5 nm, respectively. The λ_{max} remains at 424.5 nm when CYP121 (5 µM) was analysed in the presence of both clotrimazole (50 µM) and compound **6** (500 µM) (blue dashed line) indicating that the compounds do not compete for binding to the CYP121 active site.

Table S3. The g-values and proportion of high spin enzyme generated by ligands binding to CYP121 in X-band EPR spectroscopy experiments.

Compound[a]	g-values of low-spin CYP121			g-values of high-spin CYP121			HS (%)[b]
	g_z	g_y	g_x	g_z	g_y	g_x	
DMSO[c]	2.48	2.25	1.89	7.98	3.56	-	0.9
**1**	2.47	2.25	1.89	-	-	-	1.8
**2**	2.47	2.25	1.90	-	-	-	1.0
**8**	2.47	2.25	1.90	-	-	-	0.31
**9**	2.46	2.25	1.90	7.98	3.55	1.68	34
**10**	2.46	2.25	1.90	7.98	3.55	-	6.9
**12**	2.46	2.25	1.90	-	-	-	0.46
**13**	2.47	2.25	1.90	-	-	-	0.08
**20**	2.47	2.25	1.90	-	-	-	0.19
DMSO[c]	2.47	2.25	1.90	7.98	3.56	-	3.1
**17**	2.47	2.25	1.90	-	-	-	0.16
**18**	2.46	2.25	1.90	7.92	-	-	3.6
**25**	2.47	2.25	1.90	-	-	-	0.44
**27**	2.46	2.25	1.90	-	-	-	0.21
**30**	2.47	2.25	1.90	-	-	-	1.2
**31**	2.46	2.25	1.90	-	-	-	0.89
**32**	2.46	2.25	1.90	-	-	-	0.50

[a] Samples contained 100 μM CYP121 and 2 mM compounds, or 4% v/v DMSO. [b] The proportion of high-spin enzyme was calculated from the relative peak area of HS to LS g_z signals in each spectrum. [c] The g-values from 2 different spectra of ligand-free CYP121 (DMSO, 4% v/v) have been provided to account for any possible variation between different batches of the purified protein that was used to collect ligand bound spectra.

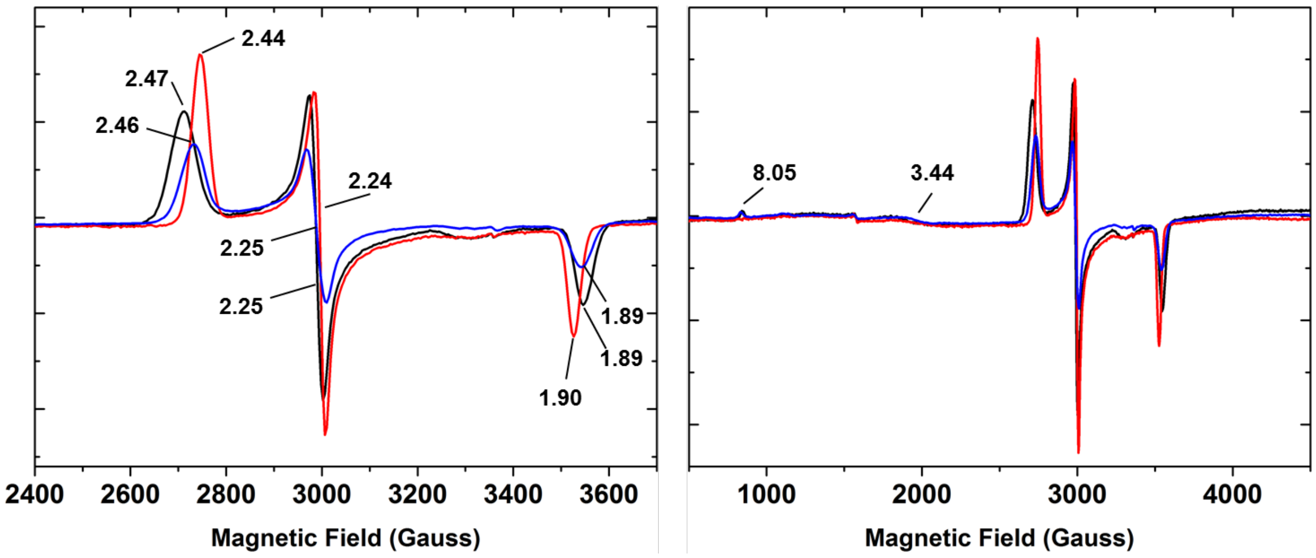

Figure S2. EPR spectra of ligand-free CYP121 (black) bound to cYY (blue) and aniline ligand **25a** (red).[1] (a) The g-values for low-spin CYP121 have been annotated for each species. (b) The g-values of high-spin CYP121 that is generated on binding to cYY have been annotated. The g_x-value could not be determined.

Table S4. The g-values and proportion of high spin enzyme generated by CYP121 binding to previously reported substrates and inhibitors (cYY, fluconazole,[2] econazole and compound **25a**[1] in X-band EPR spectroscopy experiments.

Compound[a]	G-values of low-spin CYP121			G-values of high-spin CYP121			HS (%)[b]
	g_z	g_y	g_x	g_z	g_y	g_x	
cYY	2.46	2.25	1.89	8.05	3.44	-	2.2
fluconazole[2]	2.45	2.26	1.90	-	-	-	ND
econazole	2.48	2.25	1.90	-	-	-	0
**25a**[1]	2.44	2.24	1.90	-	-	-	0

[a] Samples contained 100 µM CYP121 and 2 mM compounds, or 4% v/v DMSO. [b] The proportion of high-spin (HS) enzyme was calculated from the relative peak area of HS to LS g_z signals in each spectrum. The proportion of HS enzyme was not reported (ND) for fluconazole-bound CYP121.

Supplementary Synthetic Schemes

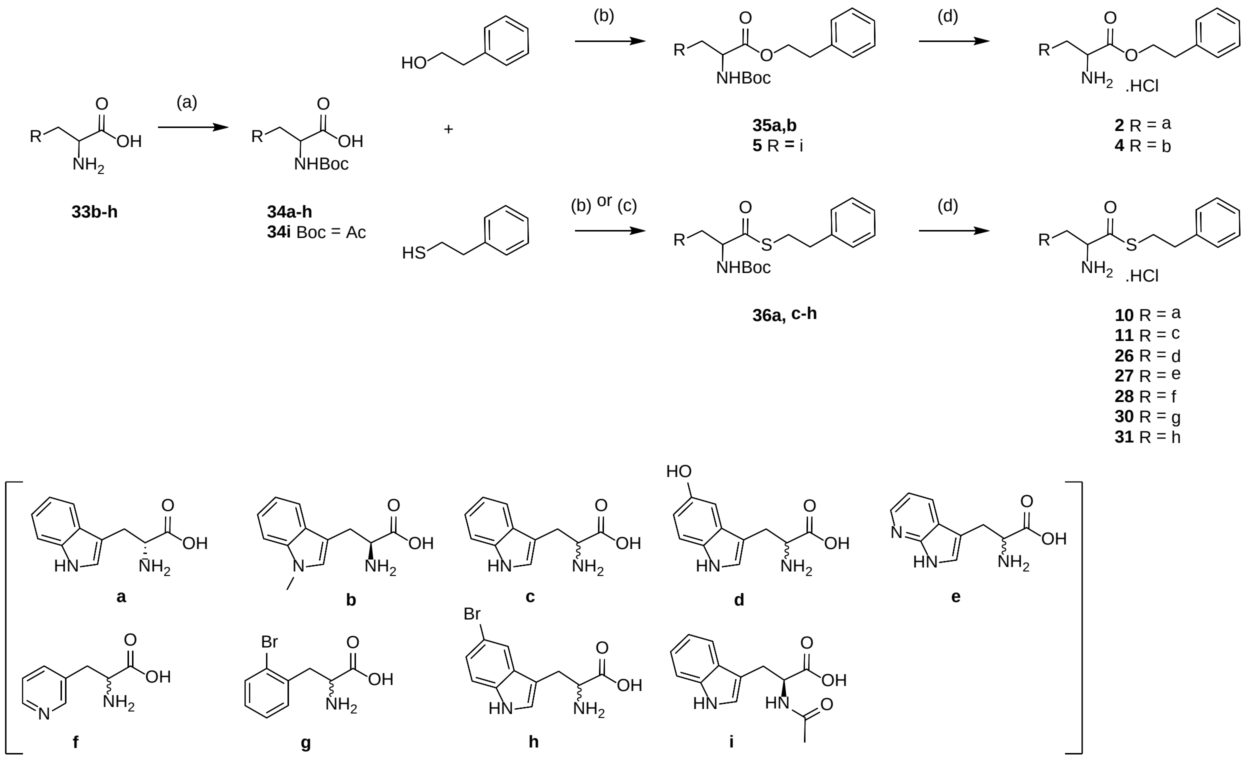

Scheme S1. Synthesis of phenethyl-ester and phenethyl-thioester amino acid analogues. *Reagents and conditions:* (a) BOC-ON®, Et_3N, 1,4-dioxane:water (1:1), rt, 5 h; (b) DCC, DMAP, DCM, 0 °C-rt, o.n.; (c) For **36d**: EDC.HCl, HOBt, DMAP, Et_3N, DCM, rt, 48h; (d) 4 M HCl in 1,4-dioxane, rt, 1 h.

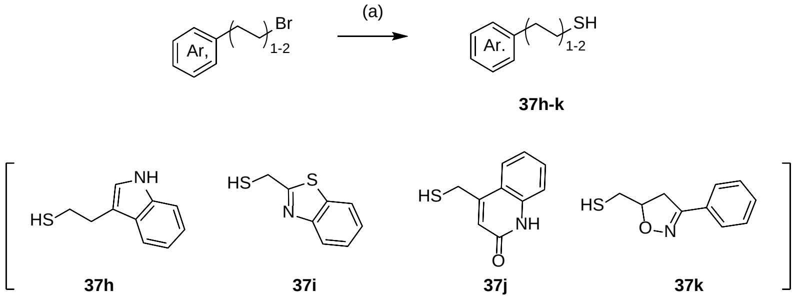

Scheme S2. Synthesis of thiols. *Reagents and conditions:* (a) thiourea, EtOH, reflux, 24 h, then 2 M NaOH, EtOH, reflux, 4 h.

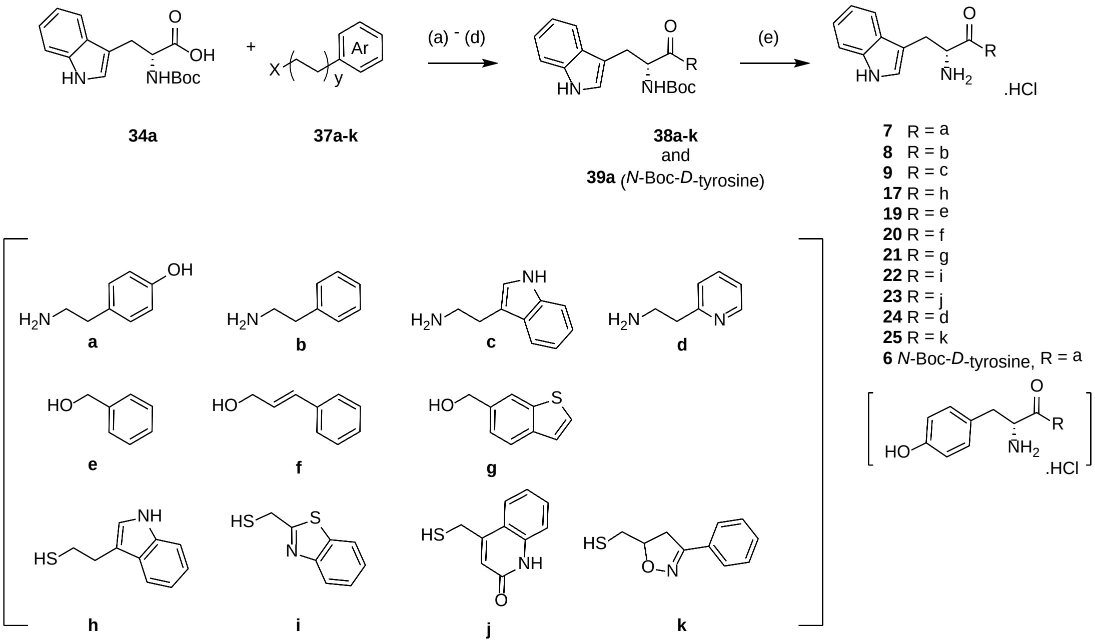

Scheme S3. Synthesis of amide, ester and thioester derivatives of D-tryptophan. *Reagents and conditions:* (a) Compounds **38a-d, 39a**: EDC.HCl, HOBt, Et_3N, DCM, rt, 24-36 h; (b) compound **38e**: EDC.HCl, Et_3N, DMAP, DCM, 0 °C-rt, o.n.; (c) DCC, DMAP, DCM, 0 °C-rt, o.n.; (d) compound **38i**: PyBOP, DIPEA, DMF, 0 °C, 40 min, rt, 3 h; (e) 4 M HCl in 1,4-dioxane, rt, 1 h, or, for **9**: 1.25 M HCl in EtOH, rt, 5 h.

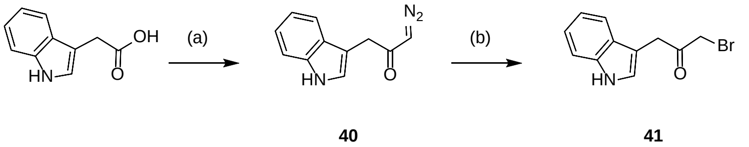

Scheme S4. Synthesis of α-bromoketone **41**. *Reagents and conditions:* (a) $SOCl_2$, DMF, THF, 0 °C, 4 h, then TMS-diazomethane, THF:MeCN, 0 °C, 4 h; (b) aq. HBr (48%), AcOH, 0 °C, 40 min.

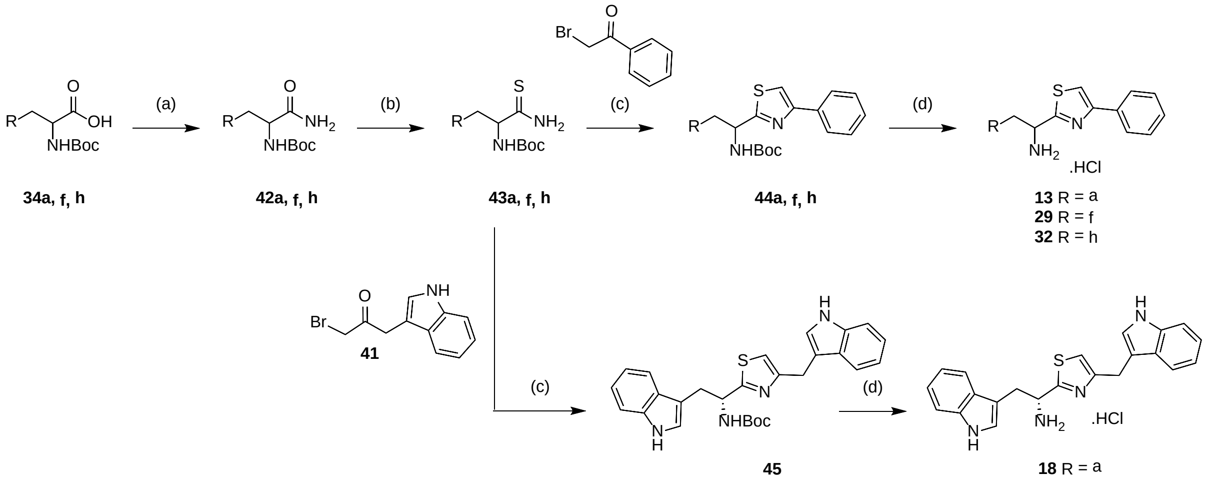

Scheme S5. Synthesis of thiazoles. *Reagents and conditions:* (a) *i*-$BuCO_2Cl$, NMO, DME, aq. NH_3 (35%), 0 °C-rt, 2 h; (b) $NaHCO_3$, P_2S_5, DME, rt, o.n., or Lawesson's Reagent, THF, rt, o.n.; (c) EtOH, rt, o.n.; (d) 4 M HCl in 1,4-dioxane, rt, 1 h.

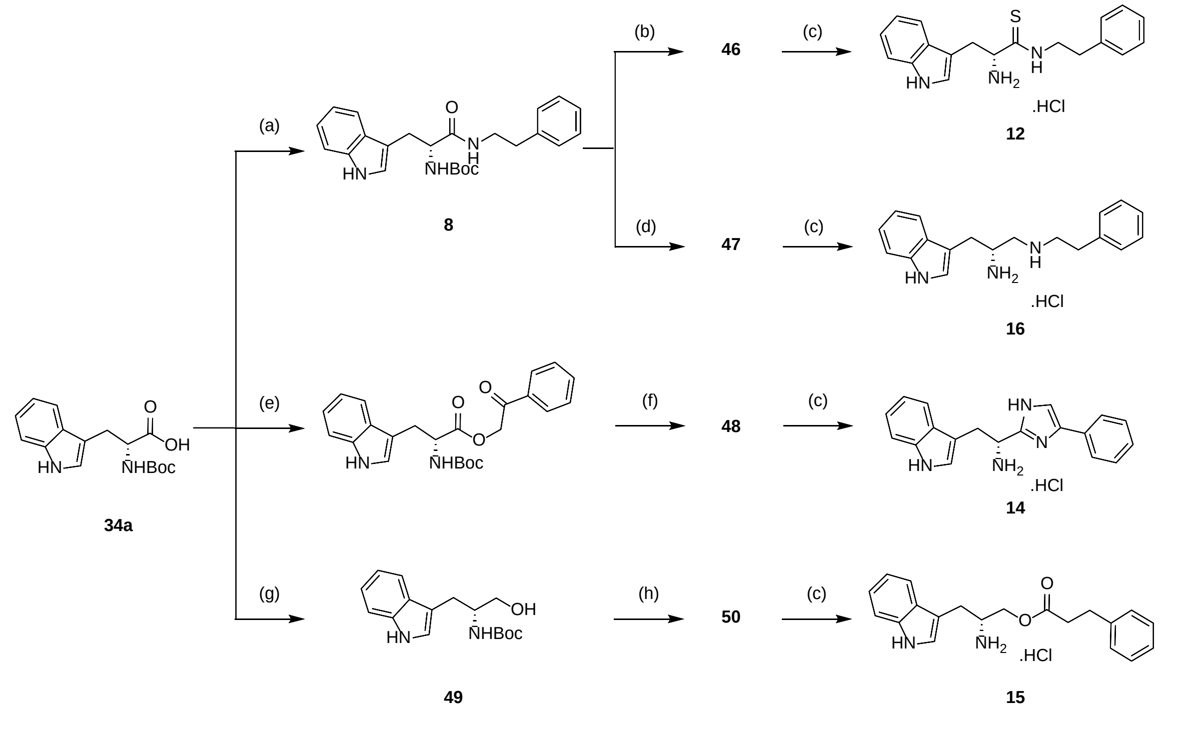

Scheme S6. Synthesis of compounds containing ester bioisosteric substitutions. *Reagents and conditions:* (a) EDC.HCl, HOBt, Et_3N, DCM, rt, 24-36 h; (b) Lawesson's reagent, THF, reflux, 36 h; (c) 4 M HCl in 1,4-dioxane, rt, 1 h; (d) Red-Al®, (2:3v/v) THF:toluene, 0-60 °C, 24 h, then 5 M NaOH, rt, 1 h; (e) (i) Cs_2CO_3, EtOH, rt, 30 min, (ii) $PhCOCH_2Br$, DMF, rt, 4 h; (f) NH_4OAc, *m*-xylene, reflux, 1 h; (g) (i) *i*BuOCOCl, NMM, DME, -15 °C, 2 min; (ii) $NaBH_4$, H_2O, -15 °C, 5 min; (h) 3-phenylpropionic acid, DCC, DMAP, DCM, 0 °C-rt, o.n.

Synthesis

All reagents were commercially sourced and used without further purification, unless otherwise specified. All reactions were conducted under the positive pressure of a dry nitrogen atmosphere. Temperatures of 0 °C and -15 °C were obtained by cooling the reaction vessel in a bath of ice, or salt and ice, respectively. Anhydrous solvents were either freshly distilled over the appropriate drying reagent (DCM and MeOH over CaH_2, THF over CaH_2 and $LiAlH_4$ using triphenylmethane as an indicator), or purchased directly from commercial sources.

Analytical thin layer chromatography (TLC) was performed using Merck glass-backed silica (Kieselgel 60 F254 0.25 mm) plates. Compounds were visualised using short wave (254 nm) or long wave (365 nm) ultra-violet light. Retention factors (R_f) are quoted with respect to the solvent system used to develop the plate. Flash column chromatography was performed using an Isolera™ Spektra One/Four purification system and the appropriately pre-packed GraceResolv™ LOK flash cartridge containing silica gel (40 µm) (Grace Discovery Sciences, USA). Solvents are reported as volume/volume eluent mixture where applicable. Reactions were monitored by TLC and LCMS to determine consumption of starting materials.

Infrared (IR) absorption spectra were recorded on a Bruker ALPHA FT-IR or Spectrum One™ FT-IR (Perkin Elmer) spectrometer by attenuated total reflectance (ATR) using a diamond crystal. Data are reported as vibrational frequency ($\upsilon_{max,}$ cm^{-1}) and intensity (strong, medium, weak or broad) for the assigned functional group.

Nuclear magnetic resonance (NMR) spectra were recorded at 300 K unless otherwise stated, using either a Bruker 400 MHz AVANCE III HD Smart Probe, 400 MHz QNP cryoprobe or 500 MHz DCH cryoprobe spectrometer. All spectra were recorded in the deuterated solvent indicated. Data are reported as chemical shift in parts per million (δ ppm) relative to the residual protonated solvent resonance peak. The relative integral, multiplicity, coupling constants (J Hz) has been provided where possible. Assignment of ^{1}H-NMR and ^{13}C-NMR spectra was assisted by DEPT, homonuclear (COSY), and heteronuclear (edited ^{1}H-^{13}C-HSQC and ^{1}H-^{13}C HMBC) 2D-NMR experiments.

Liquid chromatography mass spectrometry (LCMS) was carried out using an AQUITY UPLC H-class system (Waters, Manchester UK). Samples were either run under acidic conditions on an Acquity UPLC HSS C-18 column, eluting with a gradient of 95-5% v/v water (+ 0.1% formic acid) in MeCN, or under basic conditions on an Acquity UPLC BEH130 C18, eluting with a gradient of 95-5% v/v water (+ 10 mM NH_4OAc) in MeCN over a period of 3.5 minutes.

High resolution mass spectrometry (HRMS) was carried out using a Micromass Quadrapole-Time-of-flight (Q-Tof) mass spectrometer, Waters Xevo G2-XS QTof mass spectrometer or a ThermoFinnigan Orbitrap Classic LCMS spectrometer attached to a Dionex Ultimate 3000 HPLC. The mass to charge ratio (m/z) of the molecular ion and difference from calculated mass (δ ppm) have been quoted.

All final compounds (compounds **2-32**) had a purity of 95% or greater by LCMS analysis.

General Procedures

General procedures for the synthesis of compounds and intermediates are provided. Procedures specify the equivalents of all reagents and the typical scale (mmol) of reactions. The quantities of limiting reagents are provided with characterisation data for individual compounds.

General Procedure A: Synthesis of *N*-Boc protected amino acids 34b-h

BOC-ON® reagent (2-(*tert*-butoxycarbonyloxyimino)-2-phenylacetonitrile, Aldrich) (2.2 mmol, 1.1 equiv.) was added to a stirred solution of the amino acid (2 mmol, 1 equiv.) and Et_3N (3 mmol, 1.5 equiv.) in 1:1 v/v water:1,4-dioxane (2.4 mL). The reaction was stirred at room temperature for 4 hours and then quenched with water (15 mL). The aqueous phase was washed with EtOAc (2 x 20 mL), and then acidified with 5% citric acid solution. The product was extracted into EtOAc (3 x 20 mL) and the combined organics were dried over Na_2SO_4. The solvent was removed under reduced pressure to yield the N^{α}-Boc protected amino acid.

General Procedure B: Synthesis of thiol compounds 37h-k

Thiourea (2 mmol, 2 equiv.) was added to a solution of the alkyl bromide (1 mmol, 1 equiv.) in absolute EtOH (16 mL) and the reaction was heated under reflux for 3-16 hours. The solution was then cooled to room temperature and a solution of 2 M NaOH (4 mmol, 4 equiv.) was added. The reaction was heated under reflux for 3-4 hours, then cooled to room temperature and neutralised with 1 M HCl. The volatiles were removed under reduced pressure and then the aqueous phase was diluted with DCM (50 mL). The phases were separated and the aqueous phase was extracted with DCM (10 mL). The combined organic fractions were washed with brine (2 x 10 mL), dried over anhydrous Na2SO4 and the solvent was removed under reduced pressure. The crude product was purified by flash chromatography, eluting with the solvent system specified.

General Procedure C: Synthesis of ester compounds 5, 35a-b, 38f-g and thioester compounds 36a, 36c, 36e-h, and 38h, j-k

A solution of DCC (0.55 mmol, 1.1 equiv.) in anhydrous DCM (1 mL) was added dropwise at 0 °C to a stirred solution of the acid (0.50 mmol, 1.0 equiv.), alcohol/thiol (0.60 mmol, 1.0-1.2 equiv.) and DMAP (0.05 mmol, 0.1 equiv.) in anhydrous DCM (3 mL). The reaction was allowed to come to come slowly to room temperature and stirred overnight. The reaction was then filtered and the DCC residue was washed with DCM. The filtrate was concentrated under reduced pressure and purified by flash chromatography eluting with the solvent system specified.

General Procedure D: Synthesis of amide compounds 38a-d and 39a

EDC.HCl (1.1 mmol, 1.1 equiv.) and HOAt (1.1 mmol, 1.1 equiv.) were added to a stirred solution of *N*-Boc-*D*-tryptophan (1 mmol, 1 equiv.) in anhydrous DCM (25 mL) at 0 °C. The reaction was stirred for 10 minutes and then a solution of the amine (1 mmol, 1 equiv.) and Et_3N (1.2 mmol, 1.2 equiv) in anhydrous DCM (5 ml) was added dropwise. The reaction was allowed to warm to room temperature and stirred overnight. When complete, the reaction was diluted with DCM (25 mL) washed with water (25 ml), brine (10 ml), dried over anhydrous Na_2SO_4 and the solvent was removed under reduced pressure. Where required, the crude product was purified by flash chromatography, eluting with the solvent system specified.

General Procedure E: Synthesis of primary amide compounds 42a, f, h

Isobutylchloroformate (2.4 mmol, 1.2 equiv.) was added dropwise to a stirred solution of the *N*-Boc-protected amino acid (2 mmol, 1 equiv.) and *N*-methylmorpholine (2.4 mmol, 1.2 equiv.) in dry DME (9.2 mL) at 0 °C. The reaction was stirred for 2 minutes then 35% aqueous solution of NH_3 (740 µL)

was added dropwise. The reaction was stirred vigorously at 0 °C for 1 hour, then allowed to warm to room temperature and stirred for 3 hours. The reaction was then quenched with water (15 mL) and extracted with EtOAc (3 x 15 mL). The combined organic fractions were washed with 1 M HCl (5 mL) and brine (5 mL), dried over anhydrous Na_2SO_4 and the solvent removed under reduced pressure to yield the desired product which did not require further purification.

General Procedure F: Synthesis of thiazole compounds 44a, f, h, and 45

The primary thioamide (0.5 mmol, 1 equiv.) and α-bromoketone (0.6 mmol, 1.2 equiv.) were combined in absolute EtOH (8 mL) and the reaction was allowed to stir at room temperature for 7-20 hours. When complete, the reaction was diluted with DCM (50 mL), washed with saturated $NaHCO_3$ (30 mL), water (20 mL), brine (20 mL) and dried over anhydrous Na_2SO_4. The solvent was removed under reduced pressure and the crude material was purified by flash chromatography, eluting with the solvent system specified.

General Procedure G: Deprotection of α-amine to yield compounds 2, 4, and 6-32

A solution of anhydrous 4M HCl in dioxane (5 equiv.) was added to the Boc-protected compounds (1 equiv.) and the reaction was stirred for 1-2 hours at room temperature. The solvent was then removed under reduced pressure. The crude product was redissolved in a minimal quantity of DCM and MeOH and redried under reduced pressure to yield compounds as their hydrochloride salts.

Compound Characterisation

Phenethyl (*tert*-butoxycarbonyl)-*D*-tryptophanate (35a)

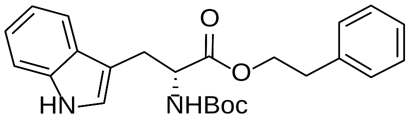

N-Boc-*D*-tryptophan (1.52 g, 4.99 mmol) was used in General Procedure C to yield **35a** as an off-white solid (1.83 g, 4.48 mmol, 90%). The crude product was purified by flash chromatography (20-80% EtOAc in Pet. ether). R_f: 0.40 (1:2 EtOAc:Pet. ether); ^{1}H-NMR (400 MHz, $CDCl_3$) δ 8.06 (s, 1H), 7.48 (d, J = 7.69 Hz, 1H), 7.34 (m, 1H), 7.28 (m, 2H), 7.23 (m, 1H), 7.19 (m, 1H), 7.16 (m, 2H), 7.19 (app. t, J = 7.4 Hz, 1H), 6.77 (s, 1H), 5.06 (d, J = 8.2 Hz, 1H), 4.63 (m, 1H), 4.25 (m, 2H), 3.28 (m, 2H), 2.84 (t, J = 7.9 Hz, 2H), 1.43-1.31 (2 x s, 9H) ppm, ^{13}C-NMR (100 MHz, $CDCl_3$) δ 172.3, 155.3, 137.7, 136.1, 129.1, 128.7, 127.8, 126.8, 122.8, 122.2, 119.7, 118.9, 111.2, 110.2, 79.9, 66.0, 54.4, 35.0, 28.5, 28.0 ppm; IR (solid) v_{max} 3346 (m, br, N-H), 2976 (w, C-H), 2929 (m, C-H), 2855 (w, C-H), 1693 (s, C=O), 1498 (s, C=C), 1456 (m, C=C), 1366 (m, C-H), 1250 (m, C-O), 1163 (s, C-O), 1059 (m, C-O), 1010 (w, C-H), 860 (w, C-H), 742 (s, indole C-H), 700 (m, phenyl C-H) cm^{-1}; LCMS (+ESI) *m/z* 407.1 $[M+Na]^+$, retention time 2.46 min, (95%); HRMS (+ESI) *m/z* (Calcd. $C_{24}H_{28}N_2O_4Na$ $[M+Na]^+$ = 431.1947), *Obs.* 431.1967 (δ ppm = 4.6).

Phenethyl *D*-tryptophanate hydrochloride (2)

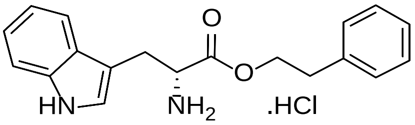

Compound **35a** (100.7 mg, 0.247 mmol) was used in General Procedure G to yield compound **2** as an off-white amorphous solid (78.4 mg 0.227 mmol, 92%). ^{1}H-NMR (400 MHz, d_6-DMSO) δ 11.10 (s, 1H), 8.61 (s, 3H), 7.40 (d, J = 7.9 Hz, 1H), 7.37 (d, J = 8.1 Hz, 1H), 7.27 (app. t, J = 7.2 Hz, 2H), 7.21 (m, 1H), 7.15 (m, 3H), 7.09 (app. t, J = 7.5 Hz, 1H), 6.99 (app. t, J = 7.4 Hz, 1H), 4.23-4.13 (m, 3H), 3.28 (dd, J = 15.1, 5.7 Hz, 1H), 3.21 (dd, J = 14.8, 6.9 Hz, 1H), 2.74 (m, 2H) ppm, ^{13}C-NMR (100 MHz, d_6-DMSO) δ 169.3, 137.5, 136.2, 128.9, 128.4, 126.9, 126.5, 124.9, 121.2, 118.6, 118.0, 111.6, 106.4, 66.1, 52.7, 33.9, 26.2 ppm;. IR (solid) v_{max} 3287 (m, br, N-H), 2919 (m, s, C-H), 2852 (m, br, NH_3Cl), 1743 (s, C=O), 1626 (m, C=C), 1575 (m, C=C), 1498 (m, C=C), 1459 (m, C=C), 1358 (m, C-H), 1234 (m, C-O), 1105 (m, C-O), 1083, 817 (w, C-H), 739 (s, indole C-H), 698 (s, phenyl C-H) cm^{1}; LCMS (+ESI) *m/z*: 309.2 [M+H]$^+$, retention time 1.65 minutes, (97%); HRMS (+ESI) *m/z* (Calcd. $C_{19}H_{20}N_2O_2$ [M+H]$^+$ = 309.1603), *Obs.* 309.1603 (δ ppm = 0.0).

N$^{\alpha}$-(*tert*-butoxycarbonyl)-1-methyl-*L*-tryptophan (34b)

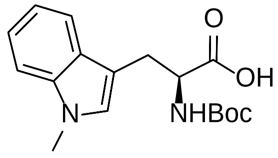

1-Methyl-*L*-tryptophan (175 mg, 0.80 mmol) was used in General Procedure A to yield compound compound **34b** as a pale-brown amorphous solid (189 mg, 0.59 mmol, 74%).

^{1}H-NMR (500 MHz, d_6-DMSO) δ 7.53 (d, J = 7.9 Hz, 1H), 7.36 (d, J = 8.1 Hz, 1H), 7.12 (ddd, J = 8.1, 6.9, 1.2 Hz, 1H), 7.09 (s, 1H), 7.01 (app. t, J = 7.4 Hz, 1H), 6.83 (d, J = 7.9 Hz, 1H), 4.11 (app.td, J = 8.3, 4.7 Hz, 1H), 3.71 (s, 3H), 3.13 (dd, J = 14.5, 4.8 Hz, 2H), 2.97 (dd, J = 14.6, 8.8 Hz, 1H), 1.33-1.19 (2s, 9H, (Boc)) ppm; ^{13}C-NMR (125 MHz, d_6-DMSO) δ 173.9, 155.3, 136.5, 127.9, 127.7, 121.0, 118.5, 118.4, 109.7, 109.5, 77.9, 54.8, 45.3, 32.3, 28.2 ppm; IR (solid) v_{max} 3400-3300 (w, br, O-H, N-H), 2977, 2925 (w, C-H), 1706 (br, s, C=O), 1502, 1475 (m, N-H, O-H), 1366 (m, CH_3), 1249 (m, C-N), 1159 (s, C-O), 1056, 1013 (m, C-N), 858 (w, C-H), 737 (s, indole-C-H) cm^{-1}; LCMS (+ESI) *m/z* 317.2 [M-H]$^-$, retention time 2.16 min, (89%); HRMS (+ESI) *m/z* (Calcd. $C_{17}H_{23}N_2O_4$ [M+H]$^+$ = 319.1652), *Obs.* 319.1648 (δ ppm = 1.3).

Phenethyl *N*$^{\alpha}$-(*tert*-butoxycarbonyl)-1-methyl-*L*-tryptophanate (35b)

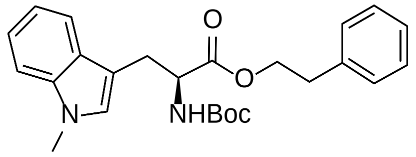

Compound **34b** (171 mg, 0.54 mmol) and phenethyl alcohol (65 µL, 0.54 mmol) were used in General Procedure C to yield compound **35b** as a brown oil (153 mg, 0.36 mmol, 67%). The crude product was purified by flash chromatography (20-100% v/v EtOAc in Pet. ether). R_f 0.59 (50% v/v EtOAc in Pet ether); ^{1}H-NMR (500 MHz, $CDCl_3$) δ 7.47 (d, J = 7.9 Hz, 1H), 7.29 (dd, J = 7.4, 7.0 Hz, 2H), 7.25 (m, 1H), 7.23 (m, 1H), 7.20 (m, 1H), 7.17 (d, J = 6.9 Hz, 2H), 7.09 (app. t, J = 7.3 Hz, 1H), 6.58 (s, 1H), 5.03 (d, J = 8.3 Hz, 1H), 4.62 (dt, J = 8.4, 5.4 Hz, 1H), 4.27 (td, J = 6.9, 1.7 Hz, 2H), 3.69 (s, 3H), 3.21 (m, 2H), 2.85 (t, J = 7.0 Hz, 2H), 1.43 (s, 9H) ppm; ^{13}C-NMR (125 MHz, $CDCl_3$) δ 172.3, 155.4, 137.9, 137.0, 129.1, 128.7, 128.4, 127.5, 126.8, 121.8, 119.2, 119.0, 109.3, 108.7, 79.9, 66.0, 54.5, 35.1, 32.8, 28.5, 28.0 ppm; IR (solid) v_{max} 3432 (w, br, N-H), 2977, 2929 (w, C-H), 1738, 1709 (s, C=O), 1615 (w, C=C), 1497, 1455 (m, C=C, C-H), 1365, 1325 (m, C-N, CH_3), 1249 (m, C-N, C-O), 1157 (s, C-O), 1058, 1011 (m, C-N), 860 (w, C-H), 737 (s, indole-C-H), 699 (s, phenyl C-H) cm^{-1}; LCMS (+ESI) *m/z* 445.3 [M+H]$^+$,

retention time 2.58 min, (94%); HRMS (+ESI) *m/z* (Calcd. $C_{15}H_{30}N_2O_4Na$ $[M+Na]^+$ = 445.2098), *Obs.* 445.2092 (δ ppm = 1.3).

Phenethyl 1-methyl-*L*-tryptophanate hydrochloride (4)

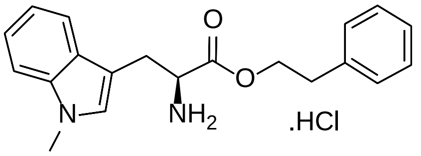

Compound **35b** (57 mg, 0.14 mmol) was used in General Procedure G to yield compound **4** as a white solid (46 mg, 0.13 mmol, 92%). ^{1}H-NMR (500 MHz, d_6-DMSO) *δ* 8.59 (s, 3H), 7.41 (dd, *J* = 8.8, 2.3 Hz, 2H), 7.27 (dd, *J* = 8.0, 6.6 Hz, 2H), 7.21 (dt, *J* = 7.4, 2.2 Hz, 1H), 7.19–7.13 (m, 3H), 7.08 (s, 1H), 7.03 (ddd, *J* = 7.9, 7.0, 0.9 Hz, 1H), 4.22 (t, *J* = 6.8 Hz, 2H), 4.14 (dd, *J* = 7.0, 5.7 Hz, 1H), 3.72 (s, 3H), 3.26 (dd, *J* = 14.8, 5.7 Hz, 1H), 3.20 (dd, *J* = 14.8, 7.0 Hz, 1H), 2.83–2.68 (m, 2H) ppm; ^{13}C-NMR (125 MHz, d_6-DMSO) *δ* 169.3, 137.6, 136.6, 129.1, 128.9, 128.4, 127.2, 126.5, 121.3, 118.8, 118.21, 109.8, 105.7, 66.1, 52.7, 33.9, 32.4, 25.9 ppm; IR (solid) v_{max} 3032 (m, br, N-H), 2818 (m, br, C-H), 2631 (w, N-H), 1741 (s, C=O), 1573 (w, C=C), 1496, 1475 (m, C=C, C-H), 1326 (m, C-N), 1253 (m, C-N, C-O), 1224 (s, C-O), 1073, 1060 (m, C-N), 832 (w, C-H), 739 (s, indole-C-H), 699 (s, phenyl C-H) cm^{-1}; LCMS (+ESI) *m/z* 324.3 $[M+H]^+$, retention time 1.99 min, (95%); HRMS (+ESI) *m/z* (Calcd. $C_{20}H_{23}N_2O_2$ $[M+H]^+$ = 323.1754), *Obs.* 323.1749 (*δ* ppm = 1.5).

Phenethyl acetyl-*L*-tryptophanate (5)

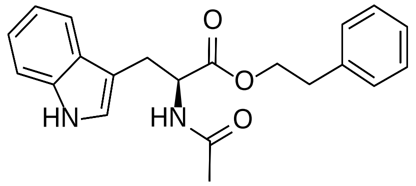

N-Acetyl-L-tryptophan **34i** (133 mg, 0.6 mmol) and 2-phenethyl alcohol (72 µL, 0.6 mmol) were used in General Procedure C to yield compound **5** as white solid (69 mg, 0.20 mmol, 33%). The crude product was purified by flash chromatography (20-100% v/v EtOAc in Pet. ether, then 10% v/v MeOH in EtOAc). R_f 0.35 (50% v/v EtOAc in Pet. ether); ^{1}H-NMR (500 MHz, $CDCl_3$) *δ* 8.09 (br, s, 1H), 7.46 (dd, *J* = 8.0, 1.0 Hz, 1H), 7.34 (dt, *J* = 8.1, 0.9 Hz, 1H), 7.32–7.27 (m, 2H), 7.23 (tt, *J =* 7.4, 1.4 Hz, 1H), 7.19 (m, 1H), 7.18–7.14 (m, 2H), 7.10 (ddd, *J* = 8.1, 7.0, 1.0 Hz, 1H), 5.95 (d, *J* = 8.0 Hz, 1H), 4.94 (dt, *J* = 8.0, 5.3 Hz, 1H), 4.28 (m, 2H), 3.26 (m, 2H), 2.87 (t, *J* = 6.9 Hz, 2H), 1.93 (s, 3H) ppm; ^{13}C-NMR (125 MHz, $CDCl_3$) *δ* 172.0, 169.8, 137.7, 136.2, 129.1, 128.7, 127.9, 126.8, 122.8, 122.3, 119.8, 118.7, 111.4, 110.2, 66.1, 53.3, 35.0, 27.7, 23.4 ppm; IR (solid) v_{max} 3364, 3329 (m, N-H), 2921, 2857 (w, C-H), 1728 (s, ester C=O), 1658 (amide, C=O), 1617 (w, C=C), 1537 (s, aromatic N-H), 1456, 1343, 1277 (m, CH_2, CH_3), 1229 (s, C-N, C-O), 1195 (s, C-N, C-O), 1095, 1011 (m, C-H), 838 (w, C-H), 733 (s, indole-C-H), 700 (s, phenyl C-H) cm^{-1}; LCMS (-ESI) *m/z* 349.2 $[M-H]^-$, retention time 2.10 min, (100%); HRMS (+ESI) *m/z* (Calcd. $C_{21}H_{23}N_2O_3$ $[M+H]^+$ = 351.1703), *Obs.* 351.1698 (*δ* ppm = 1.5).

S-Phenethyl *(R)*-2-((*tert*-butoxycarbonyl)amino)-3-(1*H*-indol-3-yl)propanethioate (36a)

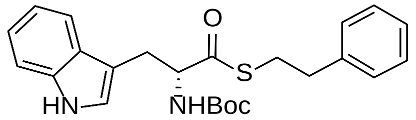

N-Boc-*D*-tryptophan (183 mg, 0.601 mmol) was used in General Procedure C to yield compound **36a** as a yellow amorphous solid (190 mg, 0.45 mmol, 75%). The crude product was purified by flash chromatography (10-50% v/v EtOAc in Pet. ether). R_f 0.44 (30% v/v EtOAc in Pet. ether); ^{1}H-NMR (400 MHz, $CDCl_3$) δ 8.04 (s, 1H), 7.58 (d, *J* = 8.0 Hz, 1H), 7.36 (d, *J* = 8.0 Hz, 1H), 7.29 (m, 2H), 7.23-7.19 (m, 2H), 7.19-7.11 (m, 3H), 6.99 (s, 1H), 5.00 (d, *J* = 8.8 Hz, 1H), 4.69 (m, 1H), 3.33 (dd, *J* = 14.8, 6.0 Hz, 1H), 3.25 (dd, *J* = 14.6, 5.4Hz, 1H), 3.09 (t, *J* = 7.6 Hz, 2H), 2.79 (t, *J* = 7.6 Hz, 2H), 1.41-1.33 (2 x s, 9H) ppm, ^{13}C-NMR (100 MHz, $CDCl_3$) δ 201.8, 155.3, 140.1, 136.1, 128.6, 128.5, 126.5, 123.0, 122.3, 119.8, 118.9, 111.1, 109.9, 80.2, 60.4, 35.5, 30.3, 28.3, 28.1 ppm; IR (solid) v_{max} 3409-3352 (m, br, N-H), 3060 (w, C-H), 3027 (w, C-H), 2933 (w, C-H), 2977 (w, C-H), 1689 (s, C=O), 1496 (s, C=C), 1456 (m, C-H), 1366 (m, C-H), 1248 (m, C-O), 1165 (s, C-O), 1093, 1011 (s, C-H), 855 (w, C-H), 742 (s, indole C-H), 698 (m, phenyl C-H) cm^{-1}; LCMS (-ESI) *m/z*: 423.2 [M-H]$^-$, retention time 2.53 minutes, (100%); HRMS (+ESI) *m/z* (Calcd $C_{24}H_{29}N_2O_3S$ [M+H]$^+$ = 425.1893), *Obs.* 425.1879 (δ ppm = 3.3).

S-Phenethyl *(R)*-2-amino-3-(1*H*-indol-3-yl)propanethioate hydrochloride (10)

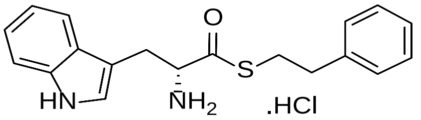

Compound **36a** (73.4 mg, 0.17 mmol) was used in General Procedure G to yield compound **10** as an off-white amorphous solid, (73.4 mg, quant.). ^{1}H-NMR (500 MHz, d_6-DMSO) δ 11.11 (s, 1H), 8.67 (s, 3H), 7.56 (d, *J* = 8.0 Hz, 1H), 7.38 (d, *J* = 8.0 Hz, 1H), 7.28 (m, 2H), 7.24 (d, *J* = 2.5 Hz, 1H), 7.21 (m, 1H), 7.14 (m, 2H), 7.08 (ddd, *J* = 8.0, 7.0, 1.0 Hz, 1H), 7.01 (ddd, *J* = 7.9, 6.9, 1.0 Hz, 1H), 4.38 (m, 1H), 3.29 (m, 2H), 3.15 (dd, *J* = 13.2, 6.9 Hz, 1H), 3.07 (dd, *J* = 13.2, 6.9 Hz, 1H), 2.70 (m, 2H) ppm, ^{13}C-NMR (125 MHz, d_6-DMSO) δ 196.6, 139.5, 136.2, 128.5, 128.4, 127.0, 126.5, 125.1, 121.2, 118.6, 118.2, 111.6, 106.2, 58.9, 34.5, 30.0, 27.5 ppm; IR (solid) v_{max} 3277 (m, br, N-H), 2988-2847 (m, br, N-H, C-H), 1688 (s, C=O) 1580 (m, C=C), 1497 (m, C=C), 1434 (m, C=C), 1352 (w, C-H), 1222 (w, C-O), 1047 (m, C-H), 826 (w, C-H), 739 (s, indole C-H), 698 (s, phenyl C-H) cm^{-1}; LCMS (+ESI) *m/z* 325.3 [M+H]$^+$, retention time 1.73, (100%); HRMS (+ESI) *m/z* (Calcd. $C_{19}H_{21}N_2OS$ [M+H]$^+$ = 325.1375), *Obs*. 325.1383), (δ ppm = 2.5).

S-Phenethyl 2-((*tert*-butoxycarbonyl)amino)-3-(1*H*-indol-3-yl)propanethioate (36c)

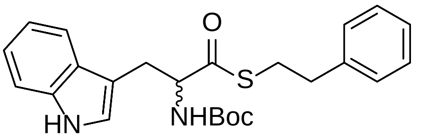

N-Boc-*D/L*-tryptophan (152.2 mg, 0.50 mmol) was used in General Procedure C to yield compound **36c** as a white solid (107 mg, 0.25 mmol, 50%). The crude product was purified by flash chromatography (0-30% v/v EtOAc in Pet. ether). R_f 0.2 (30% v/v EtOAc in Pet. ether); ^{1}H-NMR (400 MHz, $CDCl_3$) δ 8.09 (s, 1H), 7.58 (d, *J* = 7.9 Hz, 1H), 7.36 (d, *J* = 8.1 Hz, 1H), 7.29 (app.t, *J* = 7.3 Hz, 2H), 7.25–7.10 (m, 5H), 6.98 (d, *J* = 2.0 Hz, 1H), 5.01 (d, *J* = 8.7 Hz, 1H), 4.69 (dt, *J* = 8.8, 5.8 Hz, 1H), 3.32 (dd, *J* = 15.0, 6.0 Hz, 1H), 3.25 (dd, *J* = 14.7, 5.5 Hz, 1H), 3.09 (t, *J* = 7.5 Hz, 2H), 2.79 (t, *J* = 7.6 Hz, 2H), 1.42 (s, 9H) ppm; ^{13}C-NMR (100 MHz, $CDCl_3$) δ 201.8, 155.3, 140.1, 136.2, 128.8, 128.6, 127.8, 126.6, 123.1, 122.5, 119.9, 119.0, 111.3, 110.0, 80.4, 60.8, 35.7, 30.4, 28.5, 28.3 ppm; IR (solid) v_{max} 3348 (w, N-H), 2975, 2913 (w, C-H), 1687 (s, C=O), 1664 (s, C=O), 1516 (s, N-H) 1457 (w), 1366 (w), 1162 (m, C-N, C-O), 1079 (w, C-N, C-O), 971 (s, C-H), 850 (m, C-H), 740 (s, indole C-H), 694 (m, phenyl C-H)

cm^{-1}; LCMS (+ESI) *m/z* 423.3 $[M-H]^-$, retention time 2.51 minutes, (100%), HRMS (+ESI) *m/z* (Calcd. $C_{24}H_{28}N_2O_3S$ $[M+Na]^+$ = 447.1718), *Obs.* 447.1719 (δ ppm = 0.2).

S-phenethyl 2-amino-3-(1*H*-indol-3-yl)propanethioate hydrochloride (11)

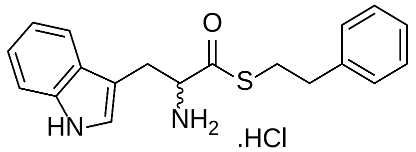

Compound **36c** (65 mg, 0.15 mmol) was used in General Procedure G to yield compound **11** as a cream coloured solid (59.4 mg, quant.). ^{1}H-NMR (500 MHz, d_6-DMSO) δ 11.10 (s, 1H), 8.67 (s, br, 3H), 7.55 (d, *J* = 7.7 Hz, 1H), 7.36 (d, *J* = 8.1 Hz, 1H), 7.27 (app.t, *J* = 7.4 Hz, 2H), 7.23 (s, 1H), 7.19 (t, *J* = 7.2 Hz, 1H), 7.13 (d, *J* = 7.5 Hz, 2H), 7.07 (app.t, *J* = 7.5 Hz, 1H), 6.99 (app.t, *J* = 7.4 Hz, 1H), 4.37 (m, 1H), 3.26 (m, 2H), 3.13 (m, 1H), 3.06 (m, 1H), 2.69 (m, 2H) ppm; ^{13}C-NMR (125 MHz, d_6-DMSO) δ 196.5, 139.5, 136.2, 128.5, 128.4, 126.9, 126.5, 125.1, 121.2, 118.6, 118.2, 111.6, 106.2, 58.9, 34.5, 30.0, 27.5 ppm; IR (solid) v_{max} 3272 (m, N-H), 3027, 2925, 2824 (m, C-H), 2594 (w, N-H), 1674 (s, C=O), 1583 (w, N-H), 1471, 1455, 1431 (m, C=C), 1349, 1338 (w, C-H, C-N), 1282, 1236, 1201 (w, C-H), 1100 (s, C-H), 1043 (s, C-N), 989, 919 (s, C-H), 737 (s, indole C-H), 697 (s, phenyl C-H), 661 (w, C-S) cm^{-1}; LCMS (+ESI) *m/z* 325.2 $[M+H]^+$, retention time 1.66 minutes, (100%); HRMS (+ESI) *m/z* (Calcd. $C_{19}H_{21}N_2OS$ $[M+H]^+$ = 325.1375), *Obs.* 325.1369 (δ ppm = 1.8).

2-((*tert*-Butoxycarbonyl)amino)-3-(5-hydroxy-1*H*-indol-3-yl)propanoic acid (34d)

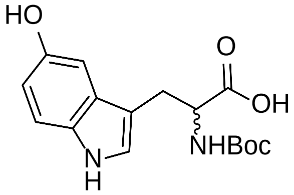

5-Hydroxy-D/L-tryptophan (440 mg, 2.00 mmol) was used in General Procedure A to yield **34d** as an off-white solid (601.1 mg, 1.88 mmol, 94%). R_f 0.2 (10% v/v MeOH in DCM); ^{1}H-NMR (500 MHz, d_6-DMSO) δ 12.49 (s, 1H), 10.50 (s, 1H), 8.60 (s, 1H), 7.12 (d, *J* = 8.7 Hz, 1H), 7.04 (d, *J* = 2.4 Hz, 1H), 6.94 (d, *J* = 8.0 Hz, 1H), 6.83 (d, *J* = 2.3 Hz, 1H), 6.58 (dd, *J* = 8.6, 2.3 Hz, 1H), 4.10 (ddd, *J* = 9.6, 7.9, 4.6 Hz, 1H), 3.02 (dd, *J* = 14.5, 4.8 Hz, 1H), 2.87 (dd, *J* = 14.5, 9.5 Hz, 1H), 1.34-1.23 (2 x s, 9H) ppm; ^{13}C-NMR (125 MHz, d_6-DMSO) δ 174.1, 155.4, 150.3, 130.6, 127.8, 124.2, 111.7, 111.2, 109.1, 102.0, 78.0, 54.3, 28.2, 27.0 ppm; IR (solid) v_{max} 3282, 3202 (m, N-H), 2972 (w, C-H), 1688 (m, C=O), 1659 (s, C=O), 1621 (m, C-O), 1521 (m, N-H), 1489 (m, CH_2), 1440, 1415 (w, CH_3), 1369 (w, C-O, C-N), 1275 (m, C-O, C-N), 1211 (m, C-O), 1157 (s, CH_2), 1103, 1068, 1022 (m, C-N, C-H), 935, 854 (w, C-H), 788 (w, indole C-H) cm^{-1}; LCMS (+ESI) *m/z* 320.3 $[M+H]^+$, retention time 1.46 minutes, (100%); HRMS (+ESI) *m/z* (Calcd. $C_{16}H_{20}N_2O_5Na$ $[M+Na]^+$ = 343.1270), *Obs.* 343.1268 (δ ppm = 0.6).

S-Phenethyl 2-((*tert*-butoxycarbonyl)amino)-3-(5-hydroxy-1*H*-indol-3-yl)propanethioate (36d)

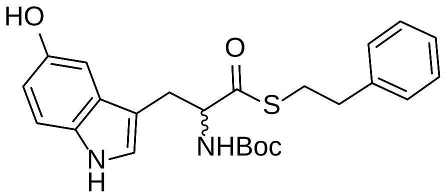

EDC.HCl (115 mg, 0.6 mmol) and HOBt (92 mg, 0.6 mmol), followed by 2-phenylethanethiol (63uL, 0.5 mmol) and a catalytic amount of DMAP were added to a stirred solution of compound **34d** (160 mg, 0.50 mmol) and Et_3N (174 uL, 1.25 mmol) in dry DCM (10 mL). The reaction was stirred at room temperature for 48 hours, then concentrated under reduced pressure and purified directly by flash chromatography (10-80% EtOAc in pet. ether) to yield compound **36d** as a brown oil (38 mg, 0.09 mmol, 17%). R_f 0.51 (10% v/v MeOH in DCM); ^{1}H-NMR (500 MHz, $CDCl_3$) δ 7.95 (s, 1H), 7.28 (m, 2H), 7.21 (app. t, *J* = 8.2 Hz, 2H), 7.15 (d, *J* = 7.6 Hz, 2H), 6.98 (d, *J* = 2.4 Hz, 1H), 6.95 (d, *J* = 2.5 Hz, 1H), 6.78 (dd, *J* = 8.7, 2.4 Hz, 1H), 5.03 (d, *J* = 8.7 Hz, 1H), 4.87 (s, 1H), 4.66 (m, 1H), 3.24 (dd, *J* = 14.9, 6.1 Hz, 1H), 3.16 (dd, *J* = 14.8, 5.6 Hz, 1H), 3.09 (tt, *J* = 7.4, 4.0 Hz, 2H), 2.78 (t, *J* = 7.7 Hz, 2H), 1.42-1.35 (2 x s, 9H) ppm; ^{13}C-NMR (125 MHz, $CDCl_3$) δ 201.9, 155.3, 149.9, 140.1, 131.5, 128.8, 128.6, 128.5, 126.6, 124.2, 112.3, 111.9, 109.4, 103.5, 80.5, 60.7, 53.9, 35.6, 30.4, 28.5 ppm; IR (solid) v_{max} 3393, 3365 (m, N-H, O-H), 2977, 2917 (w, C-H), 1686 (s, C=O), 1674 (s, C=O), 1628, 1593 (w, C=C), 1495 (s, N-H), 1473 (m, CH_2), 1369 (m, C-O), 1278, 1254 (m, C-N), 1208 (m, C-O, C-N), 1155 (s, CH_2), 1097, 1067, 1027, 1003 (m, C-N, C-H), 938, 836, 798, 739 (w, C-H), 698, 659 (w, C-S, O-H) cm^{-1}; LCMS (+ESI) *m/z* 441.3 [M+H]$^+$, retention time 2.25 minutes, (100%); HRMS (+ESI) *m/z* (Calcd. $C_{24}H_{28}N_2O_4SNa$ [M+Na]$^+$ = 463.1662), *Obs.* 463.1660 (δ ppm = 0.5).

***S*-Phenethyl 2-amino-3-(5-hydroxy-1*H*-indol-3-yl)propanethioate hydrochloride (26)**

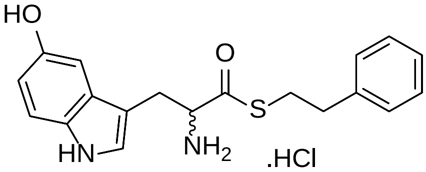

Compound **36d** (23 mg, 0.06 mmol) was used in General Procedure G to yield compound **26** as an off-white solid (27 mg, quant.). ^{1}H-NMR (400 MHz, d_6-DMSO) δ 10.78 (s, 1H), 8.69 (s, 1H), 8.61 (br, s, 3H), 7.28 (app. t, *J* = 7.7 Hz, 2H), 7.22 (d, *J* = 7.0 Hz, 1H), 7.15 (m, 4H), 6.85 (d, *J* = 2.2 Hz, 1H), 6.63 (dd, *J* = 8.6, 2.3 Hz, 1H), 4.32 (app. t, *J* = 6.6 Hz, 1H), 3.17 (m, 3H), 3.10 (m, 1H), 2.72 (m, 2H) ppm; ^{13}C-NMR (125 MHz, d_6-DMSO) δ 205.8, 150.3, 140.2, 130.7, 128.5, 128.3, 128.0, 126.2, 124.5, 111.7, 111.3, 108.6, 102.2, 61.8, 35.0, 31.0, 29.2 ppm; IR (solid) v_{max} 3300-2500 (s, br, NH_3Cl, O-H), 3234 (m, N-H), 2849 (s, C-H), 2008 (w, NH_3Cl), 1672 (s, C=O), 1627 (w, C=C), 1582 (m, N-H), 1493 (s, N-H), 1454 (s, CH_2), 1363 (m, C-O), 1207 (s, C-H, C-N), 1114 (m, CH_2), 1040 (w, C-H, C-N), 984 (s, C-H), 915, 868, 801, 751 (m, C-H), 697 (s, C-S, C-H) cm^{-1}; LCMS (+ESI) *m/z* 341.2 [M+H]$^+$, retention time 1.71 minutes, (100%); HRMS (+ESI) *m/z* (Calcd. $C_{19}H_{21}N_2O_2S$ [M+H]$^+$ = 341.1324), *Obs.* 341.1321 (δ ppm = 0.9).

2-((*tert*-Butoxycarbonyl)amino)-3-(1*H*-pyrrolo[*2,3-b*]pyridin-3-yl)propanoic acid (34e)

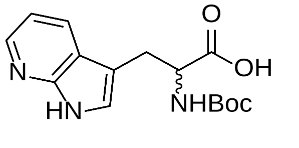

(±)-7-Azatryptophan (205.2 mg, 1 mmol) was used in General Procedure A to yield compound **34e** as a white solid (271.9 mg, 0.89 mmol, 89%). R_f 0.1 (10% v/v MeOH in EtOAc); ^{1}H-NMR (400 MHz, d_6-DMSO) δ 12.54 (s, 1H), 11.37 (s, 1H), 8.17 (dd, J = 4.7, 1.6 Hz, 1H), 7.94 (dd, J = 7.9, 1.5 Hz, 1H), 7.26 (d, J = 2.4 Hz, 1H), 7.13–6.89 (m, 2H), 4.14 (ddd, J = 9.5, 8.1, 4.8 Hz, 1H), 3.13 (dd, J = 14.5, 4.8 Hz, 1H), 2.97 (dd, J = 14.6, 9.5 Hz, 1H), 1.30 (s, 9H) ppm; ^{13}C-NMR (100 MHz, d_6-DMSO) δ 173.8, 155.4, 148.5, 142.3, 126.5, 124.2, 119.5, 114.9, 109.3, 78.0, 54.5, 28.2, 26.9 ppm; IR (solid) v_{max} 3370, 3318 (m, N-H), 2983, 2921 (w, C-H), 2368 (w, br), 1906 (w, br), 1702 (s, br, C=O), 1528 (s, N-H), 1439 (m, CH_3, CH_2), 1365 (m, CH_3), 1328 (m, C-N), 1269, 1237 (m, C-N, CH_2), 1169 (s, C-O, CH_2), 1085 (m, C-H, C-N), 1017 (C-H, C-N), 861, 804 (m, C-H), 778, 766 (s, C-H), 695 (w, C-H) cm^{-1}; LCMS (+ESI) *m/z* 306.2 [M+H]$^+$, retention time 1.38 minutes, (97%); HRMS (+ESI) *m/z* (Calcd. $C_{15}H_{19}N_3O_4$ [M+H]$^+$ = 306. 1454), *Obs.* 306. 1454 (δ ppm = 0).

S-Phenethyl 2-((*tert*-butoxycarbonyl)amino)-3-(1*H*-pyrrolo[*2,3-b*]pyridin-3-yl)propanethioate (36e)

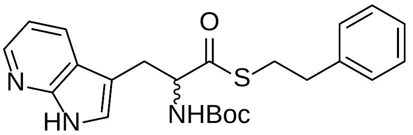

Compound **34e** (154 mg, 0.50 mmol) and 2-phenylethanethiol (81 uL, 0.60 mmol) were used in General Procedure C, using additional anhydrous DMF (100 µL) as a co-solvent, to yield compound **36e** as a white solid (54 mg, 0.13 mmol, 26%). The crude product was purified twice by flash chromatography (0-50% v/v EtOAC in Pet. ether). R_f 0.20 (50% v/v EtOAc in Pet. ether); ^{1}H-NMR (500 MHz, $CDCl_3$) δ 9.98 (s, 1H), 8.30 (dd, J = 4.8, 1.5 Hz, 1H), 7.93 (dd, J = 7.9, 1.5 Hz, 1H), 7.28 (app.t, J = 7.3 Hz, 2H), 7.21 (t, J = 7.3 Hz, 1H), 7.15 (d, J = 7.4 Hz, 2H), 7.13 (s, 1H), 7.09 (dd, J = 7.9, 4.8 Hz, 1H), 5.05 (d, J = 8.9 Hz, 1H), 4.70 (dt, J = 8.8, 5.7 Hz, 1H), 3.31 (dd, J = 15.0, 5.9 Hz, 1H), 3.23 (dd, J = 14.7, 5.6 Hz, 1H), 3.08 (t, J = 7.7 Hz, 2H), 2.78 (t, J = 7.7 Hz, 2H), 1.42-1.34 (2 x s, 9H) ppm; ^{13}C-NMR (125 MHz, $CDCl_3$) δ 201.5, 155.2, 148.3, 142.8, 139.9, 128.7, 128.6, 128.1, 126.7, 124.0, 120.6, 115.9, 108.8, 80.5, 60.6, 35.6, 30.5, 28.6, 28.5 ppm; IR (solid) v_{max} 3328 (m, N-H), 3030, 2973, 2929, 2850 (w, C-H), 1677 (s, br, C=O), 1624 (w, N-H), 1583 (w, C=C), 1528 (m, C=N), 1456 (w, C=C), 1422 (m, C=C), 1367 (m, C-H), 1327 (m, C-N, C-H), 1271 (m, C-N, C-H), 1250 (m), 1161 (m, C-O, C-H), 1074 (m, C-N) 1045 (m, C-N), 1020 (m), 774 (m, C-H), 758 (m, C-H), 692 (m, C-S), 666 (w, C-S) cm^{-1}; LCMS (+ESI) *m/z* 426.3 [M+H]$^+$, retention time 2.31 minutes, (100%); HRMS (+ESI) *m/z* (Calcd. $C_{23}H_{28}N_3O_3S$ [M+H]$^+$ = 426.1851), *Obs.* 426.1854 (δ ppm = 0.7).

S-Phenethyl 2-amino-3-(1*H*-pyrrolo[*2,3-b*]pyridin-3-yl)propanethioate hydrochloride (27)

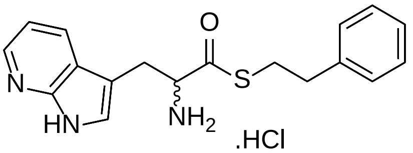

Compound **36e** (62.1 mg, 0.146 mmol) was used General Procedure G to yield compound **27**. An additional 5 equivalents of 4 M HCl in 1,4-dioxane were required and the reaction was allowed to stir for 2 hours at room temperature before the volatiles were removed under reduced pressure to obtain the tan coloured solid (62.3 mg, quant.). ^{1}H-NMR (500 MHz, d_6-DMSO) δ 12.62 (s, 2H), 8.83 (s, 3H), 8.53 (s, 1H), 8.39 (s, 1H), 7.59 (s, 1H), 7.39 (s, 1H), 7.29 (app. t, J = 7.2 Hz, 2H), 7.21 (t, J = 7.2 Hz, 1H), 7.17 (d, J = 7.4 Hz, 2H), 4.50 (s, 1H), 3.38 (m, 2H), 3.14 (m, 2H), 2.74 (m, 2H) ppm; ^{13}C-NMR (125 MHz, d_6-DMSO) δ 196.2, 141.9 (br), 139.4, 136.7 (br), 132.7 (br), 128.4 (3 x ^{13}C), 126.5, 123.2 (br), 115.2, 107.4 (br), 58.7, 34.5, 30.0,

26.7 ppm; IR (solid) v_{max} 3322, 3229 (m, N-H), 3200-2500 (s, br, NH_3Cl), 2972, 2851 (s, C-H), 2633 (m, NH_3Cl), 1683 (s, C=O), 1632 (s, NH_3Cl), 1575 (m, N-H, C=C), 1518 (s, N-H), 1437 (m, CH_2), 1335 (m, C-N), 1244 (m, CH_2), 1120, 1087, 1070 (w, C-H, C-N), 1031 (m), 985 (m, C-H), 936, 852 (w, C-H), 789 (s, C-H), 758, 698 (m, C-H), 664 (w, C-S) cm^{-1}; LCMS (+ESI) *m/z* 326.2 $[M+H]^+$, retention time 1.55 minutes, (100%); HRMS (+ESI) *m/z* (Calcd. $C_{18}H_{20}N_3OS$ $[M+H]^+$ = 326.1327), *Obs.* 326.1328 (δ ppm = 0.3).

2-((*tert*-Butoxycarbonyl)amino)-3-(pyridin-3-yl)propanoic acid (34f)

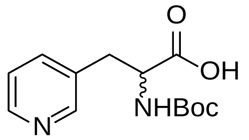

(D/L)-3-Pyridylalanine (332 mg, 2.00 mmol) was used in General Procedure A to yield compound **34f** as a white solid (321 mg, 1.21 mmol, 60%). R_f 0.10 (10% v/v MeOH in EtOAc); ^{1}H-NMR (400 MHz, d_6-DMSO) δ 12.72 (s, br, 1H), 8.44 (d, *J* = 2.2 Hz, 1H), 8.41 (dd, *J* = 4.8, 1.6 Hz, 1H), 7.66 (dt, *J* = 7.8, 2.0 Hz, 1H), 7.30 (dd, *J* = 7.8, 4.7 Hz, 1H), 7.19 (d, *J* = 8.5 Hz, 1H, NH), 4.11 (ddd, *J* = 10.6, 8.5, 4.6 Hz, 1H), 3.05 (dd, *J* = 13.9, 4.6 Hz, 1H), 2.82 (dd, *J* = 13.9, 10.6 Hz, 1H), 1.30-1.24 (2 x s, 9H) ppm; ^{13}C-NMR (100 MHz, d_6-DMSO) δ 173.3, 155.4, 150.3, 147.6, 136.6, 133.6, 123.3, 78.1, 54.6, 33.6, 28.1 ppm; IR (solid) v_{max} 3675 (w, O-H), 3307 (w, N-H), 2973, 2901 (s, br, C-H), 1699 (m, br, C=O), 1603 (w, C=C, N-H), 1520 (m, N-H), 1427 (w, C=C), 1395, 1371 (m, C-N), 1250, 1231 (m, C-O, C-H), 1165 (m, C-O, C-H), 1066, 1049 (s, br, C-H), 1021 (m, C-H), 859, 816, 785, 713, 701 (w, pyridine C-H) cm^{-1}; LCMS (+ESI) *m/z* 267.3 $[M+H]^+$, retention time 1.14 minutes, (100%); HRMS (+ESI) *m/z* (Calcd. $C_{13}H_{19}N_2O_4$ $[M+H]^+$ = 267.1345), *Obs.* 267.1350 (δ ppm = 1.9).

S-Phenethyl 2-((*tert*-butoxycarbonyl)amino)-3-(pyridin-3-yl)propanethioate (36f)

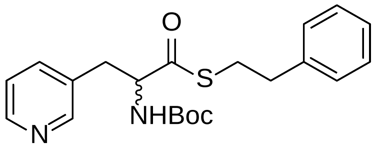

Compound **34f** (160 mg, 0.60 mmol) and 2-phenylethanethiol (96.4 mL, 0.72 mmol) were used in General Procedure C to yield compound **36f** as a white solid (160 mg, 0.41 mmol, 69%). The crude product was purified by flash chromatography (0-50% v/v EtOAc in Pet. ether). R_f 0.4 (10% v/v MeOH in DCM); ^{1}H-NMR (400 MHz, $CDCl_3$) δ 8.50 (dd, *J* = 4.8, 1.6 Hz, 1H), 8.42 (s, 1H), 7.52 (d, *J* = 7.9 Hz, 1H), 7.34–7.16 (m, 6H), 4.98 (d, *J* = 9.0 Hz, 1H), 4.65 (td, *J* = 8.2, 5.5 Hz, 1H), 3.22–3.07 (m, 3H), 3.00 (dd, *J* = 14.3, 7.5 Hz, 1H), 2.85 (t, *J* = 8.1, 2H), 1.40 (s, 9H) ppm; ^{13}C-NMR (100 MHz, $CDCl_3$) δ 200.1, 155.0, 150.3, 148.3, 139.7, 137.3, 131.9, 128.7, 128.7, 126.8, 123.7, 80.8, 60.8, 35.8, 35.7, 30.5, 28.4 ppm; IR (solid) v_{max} 3324 (m, N-H), 2979 (m, C-H), 2929 (m, C-H), 1716 (m, C=O), 1677 (s, C=O), 1575 (w, C=C), 1526 (m, N-H), 1443 (w, C=C), 1424 (w, C=C, CH_3), 1366, 1327 (m, C-N, CH_3, CH_2), 1272, 1251 (m, C-O, CH_2), 1164 (s, C-O), 1074, 1048, 1028 (m, C-H, C-N), 857 (w, C-H), 719 (m, C-H), 697 (m, C-S) cm^{-1}; LCMS (+ESI) *m/z* 388.3 $[M+H]^+$, retention time 1.98 minutes, (100%); HRMS (+ESI) *m/z* (Calcd. $C_{21}H_{26}N_2O_3S$ $[M+H]^+$ = 409.1552), *Obs.* 409.1539 (δ ppm = 3.2).

S-Phenethyl 2-amino-3-(pyridin-3-yl)propanethioate hydrochloride (28)

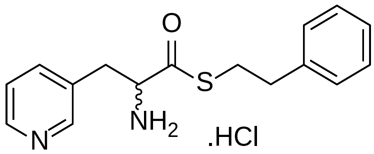

Compound **36f** (100.9 mg, 0.261 mmol) was used in General Procedure G to yield compound **28** as a yellow amorphous solid (85 mg, quant.). ^{1}H-NMR (500 MHz, d_6-DMSO) δ 8.94 (s, br, 3H), 8.82 (d, *J* = 5.3 Hz, 1H), 8.45 (d, *J* = 7.9 Hz, 1H), 7.96 (m, 1H), 7.30 (app.t, *J* = 7.5 Hz, 2H), 7.22 (m, 3H), 4.69 (t, *J* = 7.0 Hz, 1H), 3.50–3.27 (m, 2H), 3.21 (m, 2H), 2.82 (t, *J* = 7.4 Hz, 2H) ppm; ^{13}C-NMR (125 MHz, d_6-DMSO) δ 195.5, 146.3, 143.4, 141.2, 139.4, 134.2, 128.5, 128.5, 126.6, 126.5, 58.4, 34.5, 33.4, 30.1 ppm; IR (solid) v_{max} 3322 (w, N-H), 3046 (w, pyridine C-H), 3000-2500 (s, br, NH_3Cl), 2801 (s, C-H), 2635 (s, NH_3Cl), 2044 (w), 1686 (m, N-H), 1674 (s, C=O), 1596 (m, C=C), 1559 (m, C=C), 1507 (m, C=C), 1473 (m, C=C, CH_2), 1345 (w, CH_2), 1325 (w, C-H), 1155 (m, C-N), 1076 (w. C-H), 991, 942 (s, C-H), 916, 853 (m, C-H), 802 (s, C-H), 751 (m, C-H), 697 (s, C-H), 682 (s, C-S) cm^{-1}; LCMS (+ESI) *m/z* 287.2 $[M+H]^+$, retention time 1.36 minutes, (100%); HRMS (+ESI) *m/z* (Calcd. $C_{16}H_{19}N_2OS$ $[M+H]^+$ = 287.1218), *Obs.* 287.1207 (δ ppm = 3.8).

3-(2-Bromophenyl)-2-((*tert*-butoxycarbonyl)amino)propanoic acid (34g)

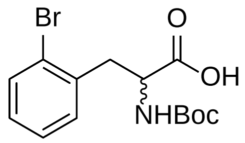

L-2-Bromophenylalanine (610.2 mg, 2.5 mmol) was used in General Procedure A to yield compound **34g** as a white solid (532.3 mg). The crude product was used without further purification. R_f 0.2 (10% v/v MeOH in DCM); ^{1}H NMR (400 MHz, d_6-DMSO) δ 12.70 (s, 1H), 7.58 (dd, *J* = 8.0, 1.2 Hz, 1H), 7.35 (dd, *J* = 7.7, 1.9 Hz, 1H), 7.30 (ddd, *J* = 7.4, 7.4, 1.3 Hz, 1H), 7.25–7.12 (m, 2H), 4.21 (ddd, *J* = 10.7, 8.7, 4.4 Hz, 1H), 3.20 (dd, *J* = 13.8, 4.4 Hz, 1H), 2.89 (dd, *J* = 13.9, 10.8 Hz, 1H), 1.29 (2 x s, 9H) ppm; ^{13}C-NMR (100 MHz, d_6-DMSO) δ 173.3, 155.4, 137.1, 132.5, 132.0, 128.7, 127.5, 124.1, 78.1, 52.9, 36.9, 28.2 ppm; IR (solid) v_{max} 3352 (m, N-H), 3000-2500 (br, w, O-H), 2953, 2925 (m, C-H), 1728 (s, C=O), 1655 (s, C=O), 1475, 1457 (m, C=C, CH_2), 1394 (s, O-H, CH_3), 1367 (s, CH_2, CH_3), 1202 (s, C-O), 1155 (s, CH_2), 1061, 1030, 1017 (s, C-H, C-N), 872, 854, 823, 787 (w, C-H), 752 (s, C-H), 661 (w, C-Br) cm^{-1}; LCMS (+ESI) *m/z* 342.0 $[M-H]^-$, retention time 2.00 minutes, (87%); HRMS (+ESI) *m/z* (Calcd. $C_{14}H_{18}NO_4Br$ $[M+Na]^+$ = 366.0317), *Obs.* 366.0301 (δ ppm = 0.3).

S-Phenethyl 3-(2-bromophenyl)-2-((*tert*-butoxycarbonyl)amino)propanethioate (36g)

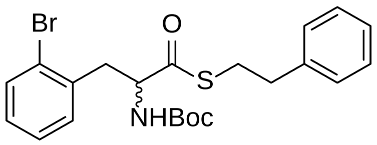

Compound **34g** (344 mg, 1.0 mmol) and 2-phenylethanethiol (161 µL, 1.2 mmol) were used in General Procedure C to yield compound **36g** as a white solid (364 mg, 0.78 mmol, 78%). The crude product was purified by flash chromatography (0-15% v/v EtOAc in Pet. ether). R_f 0.75 (50% v/v EtOAc in Pet. ether); ^{1}H-NMR (500 MHz, $CDCl_3$) δ 7.56 (d, *J* = 8.0 Hz, 1H), 7.31 (app.t, *J* = 7.5 Hz, 2H), 7.23 (m, 3H), 7.11 (t, *J* = 7.5 Hz, 1H), 5.02 (d, *J* = 9.0 Hz, 1H), 4.70 (td, *J* = 9.2, 5.0 Hz, 1H), 3.37 (dd, *J* = 14.1, 5.0 Hz, 1H), 3.14 (t, *J* = 7.3 Hz, 3H), 3.01 (dd, *J* = 14.2, 9.6 Hz, 1H), 2.87 (t, *J* = 7.2 Hz, 2H), 1.36-1.28 (2 x s, 9H) ppm; ^{13}C-NMR (125 MHz, $CDCl_3$) δ 200.5, 155.0, 140.0, 136.1, 133.1, 131.5, 128.8 (2 x ^{13}C), 128.6, 127.7, 126.67, 125.2, 80.4, 60.7, 38.7, 35.8, 30.5, 28.4, 28.1 ppm; IR (solid) v_{max} 3364 (w, N-H), 3052, 3005, 2976, 2917 (w, C-H), 1688 (s, br, C=O), 1603, 1568 (w, C=C), 1515 (s, N-H), 1444 (m, CH_2), 1391 (w, CH_3), 1366 (m, CH_3), 1316, 1293 (m, C-N, C-H), 1245 (m, br, C-O), 1163 (s, br, CH_2), 1044, 1027, 1020, 994 (m, C-H, C-N), 900, 869, 859 (w, C-H), 762 (m, C-H), 751 (s, indole C-H), 736, 723, 697 (m, C-H, C-S), 659 (w, C-Br) cm^{-1}; LCMS (+ESI) *m/z* 486.3 $[M+Na]^+$, retention time 2.65 minutes, (100%); HRMS (+ESI) *m/z* (Calcd. $C_{22}H_{26}NO_3BrSNa$ $[M+Na]^+$ = 486.0709), *Obs.* 486.0710 (δ ppm = 0.1).

S-Phenethyl 2-amino-3-(2-bromophenyl)propanethioate hydrochloride (30)

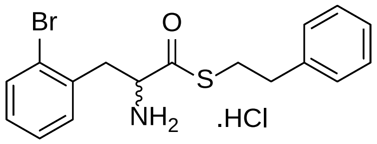

Compound **36g** (58.0 mg, 0.125 mmol) was used in General Procedure G to yield compound **30** as a white solid (50.6 mg, quant.). R_f 0.8 (10% v/v MeOH in DCM); ^{1}H-NMR (400 MHz, d_6-DMSO) δ 8.83 (s, 3H), 7.64 (d, J = 7.8 Hz, 1H), 7.42 (dd, J = 7.6, 1.5 Hz, 1H), 7.37 (t, J = 7.3 Hz, 1H), 7.26 (m, 2H), 7.22 (m, 1H), 7.18 (d, J = 7.3 Hz, 2H), 4.34 (t, J = 7.6 Hz, 1H), 3.24 (d, J = 7.6 Hz, 2H), 3.13 (m, 2H), 2.73 (m, 2H) ppm; ^{13}C-NMR (100 MHz, d_6-DMSO) δ 195.7, 139.4, 133.9, 132.8, 132.2, 129.7, 128.5, 128.4, 128.2, 126.5, 124.4, 58.1, 37.2, 34.5, 30.1 ppm; IR (solid) v_{max} 3000-2500 (m, br, N-H), 2901, 2799 (m, C-H), 2627, 2580 (m, NH_3Cl), 2025 (w, NH_3Cl), 1685 (s, C=O), 1574 (w, NH_3Cl), 1496 (m, CH_2), 1475, 1145 (m, C=C), 1374, 1350, 1268, 1223 (w, C-H), 1152, 1139 (m, CH_2), 1070 (w, C-H), 1021 (m, C-N), 987, 934 (s, C-H), 754, 707 (s, C-H), 693 (s, C-Br), 671 (w, C-S) cm^{-1}; LCMS (+ESI) *m/z* 366.0 [M+H]$^+$, retention time 1.91 minutes, (100%); HRMS (+ESI) *m/z* (Calcd. $C_{17}H_{19}NOBrS$ [M+H]$^+$ = 364.0365), *Obs.* 364.0366, (δ ppm = 0.1).

3-(5-Bromo-1*H*-indol-3-yl)-2-((*tert*-butoxycarbonyl)amino)propanoic acid (34h)

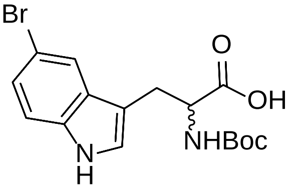

5-Bromo-D/L-tryptophan (566 mg, 2.00 mmol) was used in General Procedure A to yield compound **34h** as a white solid (550.6 mg, 1.44 mmol, 72%). R_f 0.2 (10% v/v MeOH in DCM); ^{1}H-NMR (500 MHz, d_6-DMSO) δ 12.57 (s, 1H), 11.06 (s, 1H), 7.71 (d, J = 1.9 Hz, 1H), 7.30 (d, J = 8.5 Hz, 1H), 7.21 (d, J = 2.4 Hz, 1H), 7.16 (dd, J = 8.5, 1.9 Hz, 1H), 7.01 (d, J = 8.2 Hz, 1H), 4.11 (ddd, J = 9.5, 8.1, 4.5 Hz, 1H), 3.10 (dd, J = 14.6, 4.6 Hz, 1H), 2.95 (dd, J = 14.6, 9.5 Hz, 1H), 1.31-1.21 (2 x s, 9H) ppm; ^{13}C-NMR (125 MHz, d_6-DMSO) δ 173.7, 155.3, 134.8, 129.1, 125.4, 123.3, 120.7, 113.4, 111.1, 110.3, 78.0, 54.7, 28.1, 26.7 ppm; IR (solid) v_{max} 3355 (m, N-H), 2972, 2980, (w, C-H), 2517 (w), 1710 (s, C=O), 1639 (s, C=O), 1438 (s, CH_2, CH_3), 1399 (s, O-H, CH_3), 1367 (s, C-O, CH_3), 1245 (s, C-O, CH_2), 1147 (s, C-H), 1052 (m, C-H), 948 (w), 868 (w, C-H), 797 (m, C-H), 769 (m, indole C-H), 674 (w) cm^{-1}; LCMS (+ESI) *m/z* 384.2 [M+H]$^+$, retention time 1.93 minutes, (100%); HRMS (+ESI) *m/z* (Calcd. $C_{16}H_{19}N_2O_4BrNa$ = 405.0420 [M+Na]$^+$), *Obs.* 405.0412, (δ ppm = 2.1).

S-Phenethyl 3-(5-bromo-1*H*-indol-3-yl)-2-((*tert*-butoxycarbonyl)amino)propanethioate (36h)

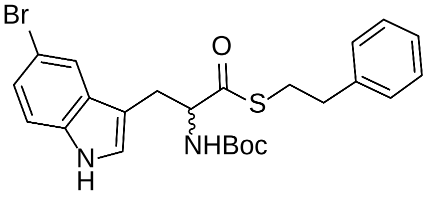

Compound **34a** (192 mg, 0.50 mmol) was used in General Procedure C to yield compound **36h** as a white amorphous solid (206 mg, 0.41 mmol, 82%). The crude product was purified by flash

chromatography (10-50% v/v EtOAc in Pet. ether). R_f 0.21 (25% v/v EtOAc in Pet. ether); ^{1}H-NMR (500 MHz, $CDCl_3$) δ 8.15 (s, 1H), 7.69 (d, *J* = 1.7 Hz, 1H), 7.30 (m, 3H), 7.23 (m, 2H), 7.17 (d, *J* = 7.3 Hz, 2H), 6.97 (d, *J* = 2.4 Hz, 1H), 5.00 (d, *J* = 9.0 Hz, 1H), 4.68 (m, 1H), 3.27 (dd, *J* = 14.9, 5.8 Hz, 1H), 3.20 (dd, *J* = 14.8, 5.4 Hz, 1H), 3.11 (app. td, *J* = 8.0, 3.6 Hz, 2H), 2.80 (app. td, *J* = 7.5, 2.8 Hz, 2H), 1.43-1.34 (2 x s, 9H) ppm; ^{13}C-NMR (125 MHz, $CDCl_3$) δ 201.3, 155.2, 140.0, 134.8, 129.6, 128.8, 128.6, 126.6, 125.3, 124.4, 121.8, 113.3, 112.8, 109.9, 80.5, 60.8, 35.6, 30.5, 28.5, 28.3 ppm; IR (solid) v_{max} 3376, 3346 (m, N-H), 3027, 2971, 2919 (w, C-H), 1696 (m, C=O), 1676 (s, C=O), 1521 (s, N-H), 1447 (w, C=C), 1351 (m, C-H, C-N), 1241 (m, C-H, C-N), 1165 (s, C-H), 1099, 1057, 1027 (w, C-N), 996 (w, C-H), 877, 853, 793 (s, C-H), 732 (s, indole C-H), 697 (s, phenyl C-H, C-Br), 661 (w, C-S) cm^{-1}; LCMS (-ESI) *m/z* 503.1 $[M-H]^-$, retention time 2.62 minutes, (100%); HRMS (+ESI): *m/z* (Calcd. $C_{24}H_{27}N_2O_3BrSNa$ $[M+Na]^+$ = 525.0818), *Obs.* 525.0804 (δ ppm = 2.6).

S-Phenethyl 2-amino-3-(5-bromo-1*H*-indol-3-yl)propanethioate hydrochloride (31)

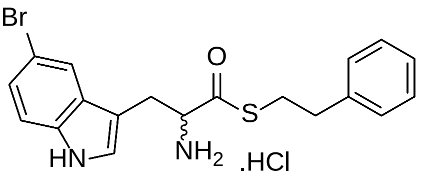

Compound **36h** (139 mg, 0.28 mmol) was used in General Procedure G to yield compound **31** as a cream coloured solid (121 mg, quant.). ^{1}H-NMR (500 MHz, d_6-DMSO) δ 11.35 (d, *J* = 2.6 Hz, 1H), 8.66 (s, 3H), 7.76 (d, *J* = 1.8 Hz, 1H), 7.34 (d, *J* = 8.6 Hz, 1H), 7.29 (d, *J* = 2.4 Hz, 1H), 7.27 (app.t, *J* = 7.4 Hz, 2H), 7.22–7.16 (m, 2H), 7.14 (d, *J* = 7.0 Hz, 2H), 4.40 (dd, *J* = 7.6, 5.7 Hz, 1H), 3.25 (m, 2H), 3.15 (m, 1H), 3.07 (m, 1H), 2.70 (m, 2H) ppm; ^{13}C-NMR (125 MHz, d_6-DMSO) δ 197.0, 140.0, 135.4, 129.4, 128.9, 128.9, 127.4, 127.0, 124.1, 121.1, 114.1, 111.9, 106.9, 59.3, 35.0, 30.4, 27.7 ppm; IR (solid) v_{max} 3275 (m, N-H), 3200-2800 (br, m, NH_3Cl), 2854 (m, C-H), 2610 (w), 1675 (s, C=O), 1587 (m), 1496, 1477, 1452, 1431 (m, CH_2), 1317, 1204 (w, C-H, CH_2), 1104 (m, C-H), 1041 (m, C-H), 995 (s), 922 (s, C-H), 886 (m, C-H), 792 (s, C-H), 745 (s, indole C-H), 696 (s, C-S, C-Br), 665 (w, C-S, C-Br) cm^{-1}; LCMS (+ESI) *m/z* 405.2 $[M+H]^+$, retention time 2.26 minutes, (100%); HRMS (+ESI): *m/z* (Calcd. $C_{19}H_{19}N_2OBrSNa$ $[M+Na]^+$ = 425.0299), *Obs.* 425.0295 (δ ppm = 0.9).

tert-Butyl *(R)-(*1-((4-hydroxyphenethyl)amino)-3-(1*H*-indol-3-yl)-1-oxopropan-2-yl)carbamate (38a)

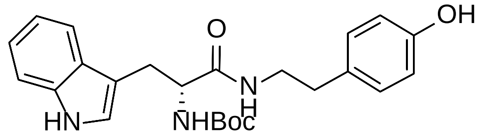

N-Boc-*D*-tryptophan (1.52 g, 4.99 mmol) was used in General Procedure D to yield **38a** as a yellow amorphous solid (1.73 g, 4.25 mmol, 85%). The crude product was purified by flash chromatography (20-80% v/v EtOAc in Pet. ether). R_f (50% v/v EtOAc in Pet. ether); ^{1}H-NMR (400 MHz, MeOD) δ 7.58 (d, *J* = 7.8 Hz, 1H), 7.33 (d, *J* = 8.1 Hz, 1H), 7.10 (ddd, *J* = 8.1, 6.9, 1.2 Hz, 1H), 7.06 (s, 1H), 7.02 (app. t, *J* = 7.5 Hz, 1H), 6.88 (d, *J* = 8.0 Hz, 2H), 6.66 (d, *J* = 8.0 Hz, 2H), 4.27 (t, *J* = 6.9 Hz, 1H), 3.34 (m, 1H), 3.19 (m, 1H), 3.15 (d, *J* = 6.4 Hz, 1H), 3.02 (dd, *J* = 14.2, 7.4 Hz, 1H), 2.46 (m, 2H), 1.37-1.21 (2 x s, 9H) ppm, ^{13}C-NMR (100 MHz, MeOD) δ 174.6, 157.5, 156.9, 138.0, 131.1, 130.7, 128.9, 124.5, 122.4, 119.8, 119.5, 116.2, 112.3, 111.0, 80.6, 57.1, 42.3, 35.4, 29.4, 28.6 ppm; IR (solid) v_{max} 3313 (m, br, N-H), 2972, 2917 (w, C-H), 1648 (m, br, C=O) 1514 (s, N-H), 1455 (m, C=C), 1366 (w, CH), 1230 (m, C-N, C-O), 1160 (s, CH_2, C-O), 1066 (w, C-H, C-N), 827 (w, C-H), 741 (s, indole C-H) cm^{-1}; LCMS (+ESI)$^-$

m/z: 424.3 $[M+H]^+$, retention time 1.96 minutes (94%); HRMS (+ESI) *m/z* (Calcd. $C_{24}H_{30}N_3O_4$ 424.2231 $[M+H]^+$), *Obs*. 424.2216 (δ ppm = 3.5).

_(R)_-2-Amino-_N_-(4-hydroxyphenethyl)-3-(1_H_-indol-3-yl)propanamide hydrochloride (7)

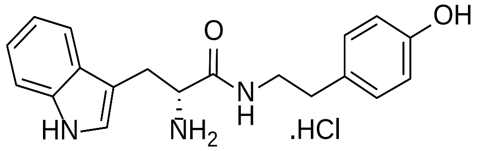

Compound **38a** (41.2 mg, 0.097 mmol) was used in General Procedure D to yield compound **7** as an off-white amorphous solid (37.9 mg, quant.). ^{1}H-NMR (400 MHz, d_6-DMSO) δ 11.05 (s, 1H), 8.60 (s, 1H), 8.20 (s, 3H), 7.65 (d, *J* = 7.8 Hz, 1H), 7.36 (d, *J* = 8.1 Hz, 1H), 7.19 (d, *J* = 2.0 Hz, 1H), 7.09 (dd, *J* = 7.6, 7.2 Hz), 7.00 (dd, *J* = 7.6, 7.2 Hz, 1H), 6.93 (d, *J* = 8.4 Hz, 2H), 6.66 (d, *J* = 8.4 Hz, 2H), 3.90 (m, 1H), 3.29 (m, 1H), 3.13 (m, 2H), 3H obscured ppm, ^{13}C-NMR (100 MHz, d_6-DMSO) δ 168.2, 155.8, 136.3, 129.5, 129.1, 127.1, 124.8, 121.1, 118.5, 118.4, 115.1, 111.5, 107.0, 52.8, 40.8, 34.0, 27.4 ppm; IR (solid) v_{max} 3500-2500 (m, br, N-H, O-H), 3231 (m, N-H), 2920 (m, C-H), 1665 (s, C=O), 1613 (m, C=C), 1594 (m, C=C), 1545 (m), 1513 (s, N-H), 1457 (s, C=C), 1341 (m, CH_2), 1225 (s, br, C-O), 1104 (m, CH_2, C-O), 1010 (m, C-H, C-N), 825 (m, C-H), 743 (s, indole C-H) cm^{-1}; LCMS (+ESI) *m/z* 324.3 $[M+H]^+$, retention time 1.35 minutes, (97%); HRMS (+ESI) *m/z* (Calcd. $C_{19}H_{22}N_3O_2$ = 324.1712 $[M+H]^+$), *Obs*. 324.1720 (δ ppm = 2.5).

***tert*-Butyl _(R)_-(3-(1_H_-indol-3-yl)-1-oxo-1-(phenethylamino)propan-2-yl)carbamate (38b)**

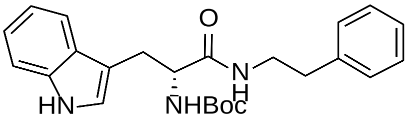

N-Boc-*D*-tryptophan (1.52 g, 4.99 mmol) was used in General Procedure D to yield compound 38b as a yellow amorphous solid (1.73 g, 4.25 mmol, 85%). The crude product was purified by flash chromatography (20-80% v/v EtOAc in Pet. ether). R_f 0.32 (50% v/v EtOAc in Pet. ether); ^{1}H-NMR (400 MHz, $CDCl_3$) δ 8.15 (s, 1H), 7.66 (d, *J* = 7.9 Hz, 1H), 7.37 (d, *J* = 8.1 Hz, 1H), 7.22 (m, 1H), 7.17 (m, 3H), 7.14 (m, 1H), 6.98 (d, *J* = 2.3 Hz, 1H), 6.88 (s, 1H), 5.64 (m, 1H, NH), 5.16 (d, *J* = 8.1 Hz, 1H, NH), 4.37 (m, 1H), 3.41 (dt, *J* = 13.3, 6.7 Hz, 1H), 3.29 (m, 2H), 3.11 (dd, *J* = 14.3, 8.2 Hz, 1H), 2.56 (dt, *J* = 13.5, 5.8 Hz, 1H), 2. 43 (dt, *J* = 14.2, 7.4 Hz, 1H), 1.41 (s, 9H) ppm; ^{13}C-NMR (100 MHz, $CDCl_3$) δ 171.6, 155.5, 138.7, 136.3, 128.7, 128.7, 127.5, 126.6, 123.2, 122.5, 119.9, 119.1, 111.4, 110.9, 80.1, 55.4, 40.6, 35.5, 28.8, 28.4 ppm; IR (solid) v_{max} 3306 (w, br, N-H), 1693 (m, br, C=O), 1655 (m, C=O), 1497 (m, N-H), 1366 (m, CH2), 1248 (m, C-N, C-H), 1162 (s, C-O, C-H), 1010 (w, C-N, C-H), 741 (s, indole C-H), 700 (s, phenyl C-H) cm-1; LCMS (-ESI) *m/z* 406.1 $[M-H]^-$, retention time 2.20 minutes, (100%); HRMS (+ESI) m/z (Calcd. $C_{24}H_{29}N_3O_3Na$ $[M+Na]^+$ = 430.2107), Obs. 430.2120, (δ ppm = 3.0).

_(R)_-2-Amino-3-(1_H_-indol-3-yl)-_N_-phenethylpropanamide hydrochloride (8)

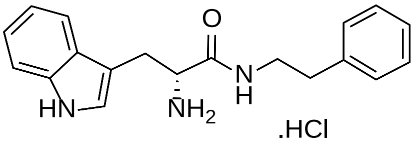

Compound **38b** (104 mg, 0.26 mmol) was used in General Procedure G to yield compound **8** as a brown amorphous solid (73.6 mg, 0.21 mmol, 84%). ^{1}H-NMR (400 MHz, d_6-DMSO) δ 11.06 (s, 1H),

8.68 (s, 1H), 8.25 (s, 3H), 7.65 (d, *J* = 7.6 Hz, 1H), 7.36 (d, *J* = 7.9 Hz, 1H), 7.26 (m, 2H), 7.19 (m, 2H), 7.14 (d, *J* = 7.3 Hz, 2H), 7.08 (app. t, *J* = 7.2 Hz, 1H), 7.00 (app.t, *J* = 7.2 Hz, 1H), 3.92 (br s, 1H), 3.35 (m, 1H), 3.16 (m, 3H), 2.60 (m, 2H) ppm; ^{13}C-NMR (100 MHz, d_6-DMSO) δ 168.3, 139.1, 136.2, 128.6, 128.3, 127.1, 126.2, 124.8, 121.1, 118.5, 118.4, 111.4, 107.0, 52.8, 40.3, 34.7, 27.3 ppm; IR (solid) ν_{max} 3500-2500 (m, br, NH_3Cl), 3214 (m, N-H), 3024, 2917, 2860 (m, C-H), 1666 (s, C=O), 1558 (m, N-H), 1495 (m, C=C, N-H), 1455 (s, CH_2), 1436 (m, C=C), 1341 (w, CH_2, C-N), 1255 (m, CH_2, C-N), 1116 (m, C-H, C-N), 1079 (m, C-H, C-N), 961 (w, C-H), 870 (m, C-H), 742 (s, indole C-H), 699 (s, phenyl C-H) cm^{-1}; LCMS (+ESI) *m/z*: 308.2 [M+H]$^+$, retention time 1.46 minutes, (100%); HRMS (+ESI) *m/z* (Calcd. $C_{19}H_{22}N_3O$ [M+H]$^+$ = 308.1763), *Obs.* 308.1757, (δ ppm = 1.9).

tert-Butyl _(R)_-(1-((2-(1_H_-indol-3-yl)ethyl)amino)-3-(1_H_-indol-3-yl)-1-oxopropan-2-yl)carbamate (38c)

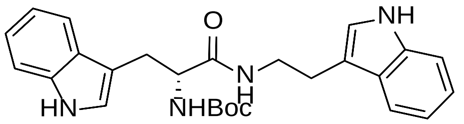

N-Boc-*D*-tryptophan (183 mg, 0.60 mmol) and tryptamine (96.5 mg, 0.602 mmol) were used in General Procedure D to yield compound **38c** as a white solid (218 mg, 0.49 mmol, 81%). The crude product was purified by flash chromatography (40-60% v/v EtOAc in Pet. ether). R_f 0.33 (2:1 EtOAc:Pet. ether); ^{1}H-NMR (400 MHz, MeOD) δ 7.58 (d, *J* = 7.9 Hz, 1H), 7.45 (d, *J* = 7.9 Hz, 1H), 7.34 (d, *J* = 8.1 Hz, 1H), 7.30 (d, *J* = 8.2 Hz, 1H), 7.11 (ddd, *J* = 7.4, 1.0 Hz, 1H), 7.07, m, 1H), 7.05 (m, 1H), 7.02 (ddd, *J* = 8.0, 7.0, 1.1 Hz, 1H), 6.97 (app, t., *J* = 7.6 Hz, 1H), 6.89 (s, 1H), 4.28 (m, 1H), 3.44 (m, 1H), 3.36 (m, 1H), 3.18 (dd, *J* = 14.4, 6.4 Hz, 1H), 3.04 (dd, *J* = 14.4, 7.2 Hz, 1H), 2.84-2.71 (m, 2H), 1.35-1.18 (2 x s, 9H) ppm; ^{13}C-NMR (100 MHz, MeOD) δ 174.6, 157.5, 138.1, 138.1, 128.9, 128.6, 124.6, 123.4, 122.4, 122.3, 119.8, 119.6, 119.5, 119.2, 113.0, 112.3, 112.2, 111.0, 80.6, 57.1, 41.3, 29.4, 28.6, 25.9 ppm; IR (solid) ν_{max} 3406, 3321 (m, N-H), 3056, 2978, 2931 (w, CH), 1698 (s, C=O), 1656 (s, C=O), 1491 (s, N-H), 1457 (s, CH_2), 1435 (m, C=C), 1391 (w, CH_3), 1366 (m, CH_3, CH_2), 1341 (m, CH_2), 1248 (m, C-H), 1162 (s, C-O, C-H), 1093, 1010 (m, C-H, C-N), 856 (w, C-H), 739 (s, indole C-H), 701 (m, indole C-H) cm^{-1}; LCMS (-ESI) *m/z* 445.3 [M-H]$^-$, retention time 2.19 minutes, (100%); HRMS (+ESI) *m/z* (Calcd. $C_{26}H_{30}N_4O_3Na$ [M+Na]$^+$ = 469.2216), *Obs.* 469.2227 (δ ppm = 2.3).

_(R)_-_N_-(2-(1_H_-Indol-3-yl)ethyl)-2-amino-3-(1_H_-indol-3-yl)propanamide hydrochloride (9)

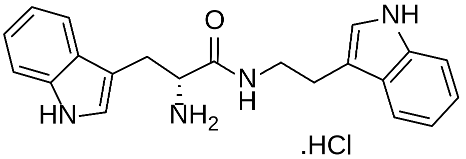

Compound **38c** (98.3 mg, 0.22 mmol) was dissolved in 1.25 M HCl in EtOH (1 ml, 1.25 mmol) and stirred for 5 hours. The volatiles were then removed under reduced pressure to yield compound **9** as an off-white powder (310 mg, quant.). ^{1}H-NMR (500 MHz, d_6-DMSO) δ 11.06 (s, 1H), 10.87 (s, 1H), 8.68 (s, 1H), 8.22 (s, 3H), 7.65 (d, *J* = 7.5 Hz, 1H), 7.51 (d, *J* = 8.0 Hz, 1H), 7.37 (d, *J* = 8.1 Hz, 1H), 7.34 (d, *J* = 8.1 Hz, 1H), 7.22 (d, *J* = 2.2 Hz, 1H), 7.11 (d, *J* = 2.2 Hz, 1H), 7.07 (m, 2H), 6.98 (m, 2H), 3.94 (m, 1H), 3.35 (m, 2H), 3.21 (dd, *J* = 14.5, 6.4 Hz, 1H), 3.13 (dd, *J* = 14.5, 7.4 Hz, 1H), 2.74 (t, *J* = 7.9 Hz, 2H) ppm; ^{13}C-NMR (125 MHz, d_6-DMSO) δ 168.2, 136.2, 127.1, 127.0, 124.8, 122.8, 121.1, 120.9, 118.5, 118.4, 118.2, 118.1, 111.4, 111.4, 107.1, 56.0, 52.9, 27.3, 24.8 ppm, 2 unresolved ^{13}C; IR (solid) ν_{max} 3400-2500 (m, br, NH_3Cl), 3397, 3250 (m, N-H), 3048, 2918, 2849 (m, C-H), 1666 (s, br, C=O), 1538 (m, N-H), 1489 (m, N-H), 1456 (m, CH_2), 1434 (m, CH_2), 1339 (w, CH_2, C-N), 1248, 1228 (w, C-H, C-N),

1096, 1078, 1009 (m, C-H, C-N), 875, 817 (w, C-H), 741 (s, indole C-H) cm^{-1}; LCMS (+ESI) *m/z* 347.2 $[M+H]^+$, retention time 1.60 minutes, (100%); HRMS (+ESI) *m/z* (Calcd. $C_{21}H_{23}N_4ONa$ $[M+H]^+$ = 347.1866), *Obs.* 347.1849 (δ ppm = 4.9).

tert-Butyl (*R*)-(3-(1*H*-indol-3-yl)-1-oxo-1-((2-(pyridin-2-yl)ethyl)amino)propan-2-yl)carbamate (38d)

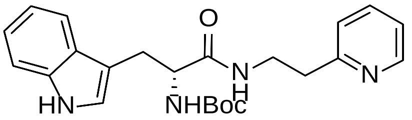

N-Boc-*D*-tryptophan (304 mg, 1.0 mmol) and 2-pyridine ethylamine (120 µL, 1.0 mmol) was used in General Procedure D to yield compound **38d** as a yellow amorphous solid (406 mg, 0.99 mmol, 99%). No purification by flash chromatography was required. ^{1}H-NMR (500 MHz, $CDCl_3$) δ 8.32 (s, 1H), 8.18 (s, 1H), 7.65 (d, *J* = 7.8 Hz, 1H), 7.50 (app.t, *J* = 7.1 Hz, 1H), 7.30 (d, *J* = 8.1 Hz, 1H), 7.17 (app.t, *J* = 7.5 Hz, 1H), 7.11 (app.t, *J* = 7.5 Hz, 1H), 7.06 (dd, *J* = 7.5, 4.9 Hz, 1H), 6.99 (d, *J* = 2.4 Hz, 1H), 6.92 (d, *J* = 6.7 Hz, 1H), 6.59 (s, 1H), 5.22 (m, 1H), 4.41 (m, 1H), 3.67–3.38 (m, 2H), 3.29 (d, *J* = 13.1 Hz, 1H), 3.13 (m, 1H), 2.76 (m, 1H), 2.66 (m, 1H), 1.41 (s, 9H) ppm; ^{13}C-NMR (125 MHz, $CDCl_3$) δ 171.5, 159.3, 155.5, 149.2, 136.6, 136.3, 127.6, 123.3, 123.1, 122.3, 121.5, 119.8, 119.1, 111.3, 111.0, 79.9, 55.4, 38.6, 36.7, 29.0, 28.5 ppm; IR (thin film) v_{max} 3297 (w, br, N-H), 2979, 2925 (w, C-H), 1701 (m, C=O), 1655 (m, C=O), 1594 (w, C=C), 1494, 1436 (m, CH_2, N-H), 1366 (m, CH_3), 1265, 1249 (m, C-N, C-O), 1164 (m, C-O), 1051 (w, C-N), 859 (w, C-H), 732 (s, indole-C-H), 701 (m, C-H) cm^{-1}; LCMS (+ESI) *m/z* 409.3 $[M+H]^+$, retention time 2.66 min, (100%); HRMS (+ESI) *m/z* (Calcd. $C_{23}H_{29}N_4O_3$ = 409.2234 $[M+H]^+$), *Obs*. 409.2228, (δ ppm = 1.5).

(*R*)-2-Amino-3-(1*H*-indol-3-yl)-*N*-(2-(pyridin-2-yl)ethyl)propanamide hydrochloride (24)

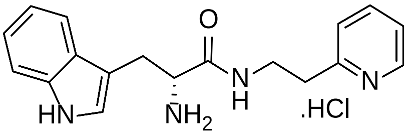

Compound **38d** (127 mg, 0.31 mmol) was used in General Procedure G to yield compound **24** as an orange amorphous solid (123 mg, quant.). ^{1}H-NMR (500 MHz, d_6-DMSO) δ 11.05 (s, 1H), 8.91 (d, *J* = 5.7 Hz, 1H), 8.73 (d, *J* = 4.9 Hz, 1H), 8.34 (s, br, 3H), 7.84 (m, 1H), 7.78 (d, *J* = 7.7 Hz, 1H), 7.62 (d, *J* = 7.9 Hz, 1H), 7.34 (d, *J* = 8.1 Hz, 1H), 7.17 (d, *J* = 2.3 Hz, 1H), 7.06 (app.t, *J* = 7.5 Hz, 1H), 6.97 (app.t, *J* = 7.5 Hz, 1H), 3.88 (m, 1H), 3.54 (m, 1H), 3.45 (m, 1H), 3.15 (m, 3H), 3.06 (m, 1H) ppm; ^{13}C-NMR (125 MHz, d_6-DMSO) δ 168.7, 154.4, 145.4, 141.5, 136.2, 127.4, 127.0, 124.9, 124.7, 121.1, 118.5, 118.4, 111.5, 107.0, 52.9, 38.1, 32.8, 27.2 ppm; IR (thin film) v_{max} 3500-2500 (m, br, NH_3Cl), 3216 (m, N-H), 3054, 2856 (m, C-H), 2627 (m, br, NH_3Cl), 1672 (s, C=O), 1618 (s, NH_3Cl), 1548 (s, N-H, NH_3Cl), 1459 (s, CH_2), 1435 (s, C=C), 1341 (m, CH_2), 1101 (m, C-H, C-N), 1008 (m, C-H, C-N), 950 (w), 870 (w), 746 (s, indole C-H), 694 (w, C-H) cm^{-1}; LCMS (+ESI) *m/z* 309.3 $[M+H]^+$, retention time 0.75 min, (100%); HRMS (+ESI) *m/z* (Calcd. $C_{18}H_{21}N_4O$ = 309.1710 $[M+H]^+$), *Obs*. 309.1701, (δ ppm = 2.9).

Benzyl (*tert*-butoxycarbonyl)-*D*-tryptophanate (38e)

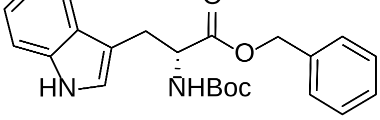

Benzyl alcohol (62 μl, 0.60 mmol), Et_3N (98 μl, 0.72 mmol) and EDC.HCl (127 mg, 0.6 mmol) were added to a stirred suspension of *N*-Boc-D-tryptophan (183 mg, 0.60 mmol) in anhydrous DCM (5 ml) and the reaction was cooled to 0 °C. A catalytic amount of DMAP was added and the solution was stirred at 0 °C for 1 hour, then allowed to warm to room temperature and stirred overnight. The reaction was then concentrated under reduced pressure and the residue was redissolved in EtOAc (25 ml), washed with water (20 ml), brine (10 ml), dried over anhydrous Na_2SO_4 and the solvent was removed under reduced pressure. The crude product was purified by flash chromatography (20-80% v/v EtOAc in Pet. ether) to yield compound **38e** as a white amorphous solid (67.6 mg, 0.17 mmol, 29%). R_f 0.47 (50% v/v EtOAc in Pet.); ^{1}H-NMR (400 MHz, $CDCl_3$) δ 8.01 (s, 1H), 7.55 (d, *J* = 8.0 Hz, 1H), 7.37 (d, *J* = 4.4 Hz, 1H), 7.33 (m, 3H), 7.23 (m, 2H), 7.18 (app.t, *J* = 7.8 Hz, 1H), 7.11 (app.t, *J* = 7.5 Hz, 1H), 6.81 (s, 1H), 5.09 (m, 2H), 4.70 (d, *J* = 6.4 Hz, 1H), 3.29 (d, *J* = 5.3 Hz, 2H), 1.42-1.31 (2 x s, 9H) ppm, ^{13}C-NMR (100 MHz, $CDCl_3$) δ 172.3, 155.4, 136.2, 135.5, 128.7, 128.6, 127.8, 127.1, 122.9, 122.3, 119.8, 119.0, 111.2, 110.2, 80.0, 67.2, 54.4, 28.5, 28.1 ppm; IR (solid) v_{max} 3412, 3350 (m, N-H), 2975, 2932 (w, C-H), 1692 (s, br, C=O), 1620 (w, C=C), 1497 (m, N-H), 1456 (m, CH_2) 1391, 1366 (m, CH3), 1352 (m, CH_2), 1159 (s, C-H, C-O), 1057 (m, C-H, C-N), 1010 (m, C-H, C-N), 858 (w, C-H), 739 (s, indole C-H), 697 (m, phenyl C-H) cm^{-1}; LCMS (-ESI) *m/z* 393.2 [M-H]$^-$, retention time 2.43 minutes, (100%); HRMS (+ESI) *m/z* calculated $C_{23}H_{26}N_2O_4Na$ [M+Na]$^+$ = 417.1790), *Obs.* 417.1797, (δ ppm = 1.7).

Benzyl *D*-tryptophanate hydrochloride (19)

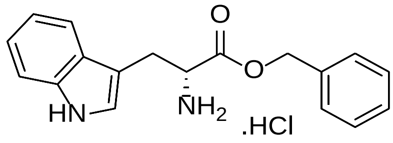

Compound **38e** (41.3mg, 0.105 mmol) was used in General Procedure G to yield compound **19** as an off-white amorphous solid (33.1 mg, 0.10 mmol, 95%). ^{1}H-NMR (400 MHz, d_6-DMSO) δ 11.10 (s, 1H), 8.64 (s, 3H), 7.52 (d, *J* = 8.0 Hz, 1H), 7.39 (d, *J* = 8.0 Hz, 1H), 7.20 (br s, 3H), 7.32 (m, 3H), 7.10 (dd, *J* = 7.6, 7.2 Hz, 1H), 7.00 (dd, *J* = 7.6, 7.2 Hz, 1H), 5.13 (d, *J* = 12.4 Hz, 1H), 5.05 (d, *J* = 12.0 Hz, 1H), 4.28 (m, 1H), 3.28 (m, 2H) ppm, ^{13}C-NMR (100 MHz, d_6-DMSO) δ 169.3, 136.2, 134.9, 128.5, 128.4, 128.2, 126.9, 125.0, 121.3, 118.7, 118.1, 111.6, 106.4, 67.0, 52.7, 26.3 ppm; IR (solid) v_{max} 3415 (w, br, N-H), 3000, 2840 (m, br, C-H), 2731, 2620, 2582 (w, NH_3Cl), 1743 (m, C=O), 1597 (w, C=C), 1498 (s, N-H), 1455 (m, CH_2), 1422 (m, C=C), 1339, 1282 (w, C-H, C-N), 1232, 1221, 1208 (s, C-O, C-N, C-H), 1145 (m), 1079 (s, C-H, C-N), 958, 908, 884, 848 (w, C-H), 733 (s, indole C-H), 698 (m, phenyl C-H) cm^{-1}; LCMS (+ESI) *m/z* 294.8 [M+H]$^+$, retention time 1.58 minutes, (100%); HRMS (+ESI) *m/z* (Calcd $C_{18}H_{19}N_2O_2$ [M+H]$^+$ = 295.1447), *Obs*. 295.1451, (δ ppm = 1.4).

Cinnamyl (*tert*-butoxycarbonyl*)*-*D*-tryptophanate (38f)

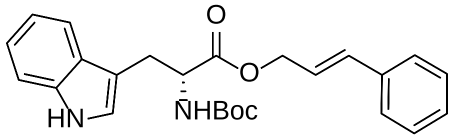

N-Boc-*D*-tryptophan (186 mg, 0.61 mmol) and cinnamyl alcohol (82.5 mg, 0.62 mmol) were used in General Procedure C to yield compound **38f** as a white solid (226 mg, 0.54 mmol, 89%). The crude product was purified by flash chromatography (20-80% v/v EtOAc in Pet. ether). R_f 0.36 (1:2 EtOAc:Pet. ether); ^{1}H-NMR (500 MHz, $CDCl_3$) δ 8.00 (s, 1H), 7.58 (d, *J* = 7.9 Hz, 1H), 7.40-7.20 (m, 5H), 7.28 (m, 1H), 7.19 (ddd, *J* = 8.2, 6.9, 1.1 Hz, 1H), 7.11 (app. t, *J* = 7.3 Hz, 1H), 7.00 (s, 1H), 6.57 (d, *J* =

15.9 Hz, 1H), 6.13 (dt, J = 15.8, 6.5 Hz, 1H), 5.11 (d, J = 8.3 Hz, 1H), 4.70 (m, 3H), 3.31 (m, 2H), 1.43-1.34 (2 x s, 9H) ppm, ^{13}C-NMR (125 MHz, $CDCl_3$) δ 172.2, 155.4, 136.2, 134.7, 128.8, 128.3, 127.9, 126.8, 123.0, 122.8, 122.3, 119.8, 119.0, 111.3, 110.4, 80.0, 66.0, 54.5, 28.5, 28.2 ppm; IR (solid) v_{max} 3396 (w, br, N-H), 3326 (m, br, N-H), 2981 (w, C-H), 2989 (m, C-H), 2851 (m, C-H), 1733 (s, C=O), 1686 (s, C=O), 1625 (m, *tran*-C=C), 1575 (m, C=C), 1510 (s, N-H), 1448 (m, C=C), 1436 (m, C=C), 1310 (w, C-H), 1216 (m, C-O), 1152 (s, C-O), 1080, 1015, 971 (m, C-H), 844 (m, C-H), 745 (s, indole C-H), 691 (m, phenyl C-H) cm^{-1}; LCMS (-ESI) *m/z* 419.1 [M-H]$^-$, retention time 2.54 minutes, (97%); HRMS (+ESI) *m/z* (Calcd. $C_{25}H_{29}N_2O_4$ [M+H]$^+$ = 421.2122), *Obs*. 421.2115, (δ ppm = 1.7).

Cinnamyl D-tryptophanate hydrochloride (20)

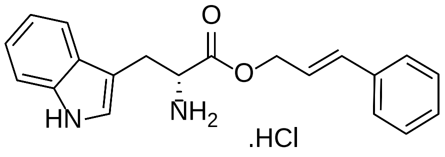

Compound **38f** (89 mg, 0.21 mmol) was used in General Procedure G to yield compound **20** as a brown solid (79 mg, quant.). ^{1}H-NMR (400 MHz, d_6-DMSO) δ 11.12 (s, 1H), 8.65 (s, 3H), 7.55 (d, J = 7.9 Hz, 1H), 7.43-7.32 (m, 4H), 7.29 (m, 2H), 7.09 (app. t, J = 7.3 Hz, 1H), 7.00 (app. t, J = 7.4 Hz, 1H), 6.61 (d, J = 16.0 Hz, 1H), 6.17 (dt, J = 16.0, 6.2 Hz, 1H), 4.72 (qd, J = 13.2, 6.2 Hz, 2H), 4.29 (m, 1H), 3.34 (m, 2H) ppm, ^{13}C-NMR (100 MHz, d_6-DMSO) δ 169.2, 156.7, 136.2, 135.8, 133.7, 128.7, 128.2, 126.5, 125.0, 122.6, 121.2, 118.6, 118.1, 111.6, 106.4, 65.9, 52.8, 33.4 ppm; IR (solid) v_{max} 3276 (m, N-H), 3000-2500 (m, br, NH_3Cl), 2927, 2852 (m, C-H), 1737 (s, C=O), 1622 (m, *trans*-C=C), 1576 (m, NH_3Cl), 1520 (m, C=C, N-H), 1494 (m, C=C, N-H), 1450 (m, CH_2), 1350 (w, CH_2), 1203 (s, C-O, C-N), 1103 (m, CH_2), 1081 (m, C-H, C-N), 962, 927 (m, C-H), 731 (s, indole C-H), 690 (m, phenyl C-H) cm^{-1}; LCMS (-ESI) *m/z* 319.1 [M-H]$^-$, retention time 1.78 minutes, (100%); HRMS (+ESI) *m/z* (Calcd. $C_{20}H_{21}N_2O_2$ [M+H]$^+$ = 321.1603), *Obs.* 321.1597, (δ ppm = 1.9).

Benzo[*b*]thiophen-5-ylmethyl (*tert*-butoxycarbonyl)-D-tryptophanate (38g)

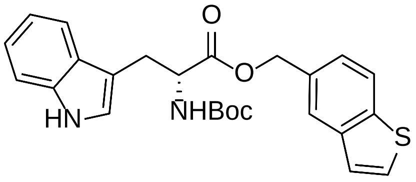

N-Boc-D-tryptophan (183 mg, 0.60 mmol) and 1-benzothiophen-5-ylmethanol (99 mg, 0.60 mmol) were used in General Procedure C to yield compound **38g** as a white amorphous solid (162 mg, 0.36 mmol, 60%). The crude product was purified by flash chromatography (10-50% v/v EtOAc in Pet. ether). R_f 0.48 (1:2 v/v EtOAc:Pet. ether); ^{1}H-NMR (400 MHz, $CDCl_3$) δ 7.87 (m, 1H), 7.82 (d, J = 8.3 Hz, 1H), 7.65 (d, J = 1.6 Hz, 1H), 7.55 (d, J = 7.9 Hz, 1H), 7.48 (d, J = 5.4 Hz, 1H), 7.31 (m, 2H), 7.19 (m, 2H), 7.09 (app.t, J = 7.5 Hz, 1H), 6.76 (d, J = 2.4 Hz,1H), 5.22 (d, J = 12.2 Hz, 1H), 5.16 (d, J = 12.2 Hz, 1H), 5.09 (d, J = 8.3 Hz, 1H), 4.70 (dt, J = 8.2, 5.5 Hz, 1H), 3.29 (d, J = 5.6 Hz, 2H), 1.60 (s, 2H), 1.42 (s, 7H) ppm, ^{13}C-NMR (100 MHz, $CDCl_3$) δ 172.3, 155.4, 139.9, 139.8, 136.2, 131.6, 127.8, 127.3, 124.9, 124.0, 123.8, 122.9, 122.7, 122.3, 119.8, 119.0, 111.2, 110.2, 80.0, 67.3, 54.4, 28.5, 28.2 ppm; IR (solid) v_{max} 3406, 3341 (m, br, N-H), 2984, 2924 (w, C-H), 1732 (s, C=O), 1684 (s, C=O), 1505 (s, N-H), 1457, 1438 (m, C=C, CH_2), 1366, 1365 (m, CH_3, C-N), 1249 (m, C-N, CH_2), 1213 (s, C-N, C-O), 1153 (s, br, C-O, C-N), 1079, 1053, 1019, 1010, (m, C-H, C-N), 949, 895, 814 (w, C-H), 742 (s, indole C-H), 711 (m, benzothiophene C-H), 664 (w, C-S) cm^{-1}; LCMS (-ESI) *m/z* 449.2 [M-H]$^-$, retention time 2.48

minutes, (95%); HRMS (+ESI) *m/z* (Calcd. $C_{25}H_{26}N_2O_4SNa$ $[M+Na]^+$ = 473.1506), *Obs*. 473.1514, (δ ppm = 1.7).

Benzo[*b*]thiophen-5-ylmethyl *D*-tryptophanate hydrochloride (21)

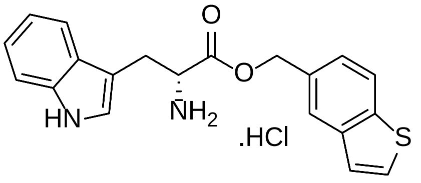

Compound **38g** (48 mg, 0.11 mmol) was used in General Procedure G to yield compounds **21** as an off-white amorphous solid (41 mg, quant.). ^{1}H-NMR (400 MHz, d_6-DMSO) δ 11.12 (s, 1H), 8.65 (s, 3H), 7.96 (d, *J* = 8.3 Hz, 1H), 7.80 (d, *J* = 5.4 Hz, 1H), 7.65 (s, 1H), 7.52 (d, *J* = 7.9 Hz, 1H), 7.42 (d, *J* = 5.4 Hz, 1H), 7.40 (d, *J* = 8.1 Hz, 1H), 7.24 (d, *J* = 2.3 Hz, 1H), 7.18 (dd, *J* = 8.3, 1.6 Hz, 1H), 7.10 (ddd, *J* = 8.1, 6.9, 1.1 Hz, 1H), 6.99 (app. t, *J* = 7.5 Hz, 1H), 5.25 (d, *J* = 12.3 Hz, 1H), 5.15 (d, *J* = 12.3 Hz, 1H), 4.29 (app. t, *J* = 6.0 Hz, 1H), 3.39-3.24 (m, 2H,) ppm, ^{13}C-NMR (100 MHz, d_6-DMSO) δ 169.4, 139.4, 139.0, 136.3, 131.0, 128.3, 126.9, 125.0, 124.5, 123.9, 123.4, 122.6, 121.2, 118.7, 118.1, 111.6, 106.5, 67.3, 52.8, 26.3 ppm; IR (solid) v_{max} 3283 (w, N-H), 3000-2800 (w, br, NH_3Cl), 2912, 2852 (m, C-H), 1743 (s, C=O), 1575 (w, NH_3Cl), 1520 (w, N-H), 1488 (m, CH_2), 1436 (w, C=C), 1357 (w, CH_2), 1306 (m, C-H), 1285 (w, C-N, C-H), 1229, 1207 (s, C-O, C-H), 1104, 1080 (m, C-H), 896, 805 (m, C-H), 741 (s, indole C-H), 693 (m, benzothiophene C-H), 665 (w, C-S) cm^{-1}; LCMS (-ESI) *m/z* 349.1 $[M-H]^-$, retention time 1.91 minutes, (100%); HRMS (+ESI) *m/z* (Calcd. $C_{20}H_{19}N_2O_2S$ $[M+H]^+$ = 351.1162), *Obs.* 351.1164, (δ ppm = 0.6).

2-(1*H*-indol-3-yl)ethane-1-thiol (37h)

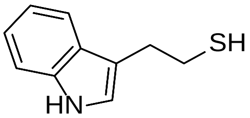

3-(2-Bromoethyl)indole (224 mg, 1 mmol) was used in General Procedure B to yield compound **37h** as a colourless oil (145 mg, 0.82 mmol, 82%). The crude product was purified by flash chromatography (0-50% v/v EtOAc in Pet. ether). R_f 0.11 (10% v/v EtOAc in Pet. ether); ^{1}H-NMR (500 MHz, $CDCl_3$) δ 7.99 (s, 1H), 7.61 (dd, *J* = 7.8, 1.0 Hz, 1H), 7.38 (ddd, *J* = 8.1, 0.9, 0.9 Hz, 1H), 7.22 (ddd, *J* = 8.2, 7.0, 1.2 Hz, 1H), 7.14 (ddd, *J* = 8.0, 7.0, 1.1 Hz, 1H), 7.07 (dd, *J* = 2.2, 1.1 Hz, 1H), 3.11 (td, *J* = 7.3, 0.8 Hz, 2H), 2.89 (m, 2H), 1.47 (t, *J* = 7.9 Hz, 1H) ppm; ^{13}C-NMR (125 MHz, $CDCl_3$) δ 136.4, 127.3, 122.3, 122.2, 119.5, 118.8, 114.3, 111.3, 30.2, 25.5 ppm; IR (solid) v_{max} 3391 (s, N-H), 3046, 2972, 2922, 2841 (w, C-H), 2570 (w, S-H), 1616, 1550 (w, N-H, C=C), 1454 (s, CH_2), 1421 (m, C=C) 1351, 1341, 1325 (m, C=C, C-N), 1243, 1220 (m, CH_2), 1090 (s, C-H), 1008 (s, C-H), 747 (s, indole C-H), 696 (m, C-S) cm^{-1}; LCMS (-ESI) *m/z* 176.1 $[M-H]^-$, retention time 2.51 minutes, (100%); HRMS (+ESI): *m/z* (Calcd. $C_{10}H_{12}NS$ $[M+H]^+$ = 178.0690), *Obs.* 178.0689 (δ ppm = 0.6).

S-(2-(1*H*-Indol-3-yl)ethyl) *(R)*-2-((*tert*-butoxycarbonyl)amino)-3-(1*H*-indol-3-yl) propanethioate (38h)

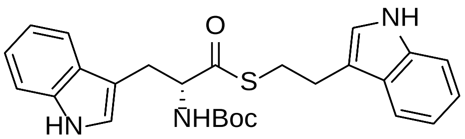

Compound **37h** (111 mg, 0.63 mmol) and boc-*D*-tryptophan (173 mg, 0.57 mmol) were used in General Procedure C to yield compound **38h** as a white amorphous solid (271 mg, 0.58 mmol, quant.). The crude product was purified by flash chromatography (10-50% v/v EtOAc in Pet. ether). R_f 0.47 (50% v/v EtOAc in Pet. ether); ^{1}H-NMR (500 MHz, $CDCl_3$) δ 7.98 (m, 2H), 7.65 (d, J = 7.9 Hz, 1H), 7.59 (d, J = 7.9 Hz, 1H), 7.37 (d, J = 7.9 Hz, 1H), 7.34 (d, J = 7.9 Hz, 1H), 7.20 (m, 2H), 7.13 (m, 2H), 6.90 (br, s, 2H), 5.03 (d, J = 8.8 Hz, 1H), 4.71 (dt, J = 8.7, 5.6 Hz, 1H), 3.33 (dd, J = 14.9, 5.8 Hz, 1H), 3.22 (m, 3H), 2.97 (t, J = 7.5 Hz, 2H), 1.42-1.33 (s, 9H) ppm; ^{13}C-NMR (125 MHz, $CDCl_3$) δ 202.0, 155.3, 136.3, 136.2, 127.8, 127.4, 123.2, 122.4, 122.2, 122.1, 119.9, 119.6, 119.0, 114.5, 111.3, 109.9, 80.3, 60.8, 29.7, 28.5, 28.3, 25.3 ppm, 2 unresolved aromatic ^{13}C; IR (solid) v_{max} 3397-3333 (m, br, N-H), 2974, 2931, 2850 (w, C-H), 1692 (s, C=O), 1680 (s, C=O), 1492 (m, N-H), 1456 (m, CH_2), 1366 (w, C=C, CH_3), 1341 (w, CH_2, C-N), 1247 (m, N-H, C-N), 1091, 1061, 1010 (C-H, C-N), 852 (w, C-H), 738 (s, indole C-H) cm^{-1}; LCMS (+ESI) *m/z* 364.4 [M(-Boc)+H]$^+$, retention time 2.40, (100%); HRMS (+ESI): *m/z* (Calcd. $C_{26}H_{29}N_3O_3SNa$ [M+Na]$^+$ = 486.1827), *Obs.* 486.1827 (δ ppm = 0.0).

S-(2-(1*H*-Indol-3-yl)ethyl) *(R)*-2-amino-3-(1*H*-indol-3-yl)propanethioate hydrochloride (17)

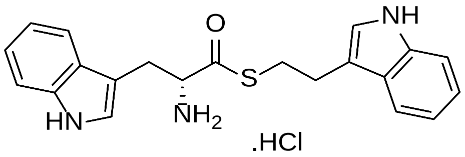

Compound **38h** (85.0 mg, 0.183 mmol) was used in General Procedure G to yield compound **17** as a pink solid (73.9 mg, 0.185 mmol, quant.). ^{1}H-NMR (500 MHz, d_6-DMSO) δ 11.10 (s, 1H), 10.90 (s, 1H), 8.65 (s, 3H), 7.55 (2d, J = 8.3, 8.3 Hz, 2H), 7.36 (d, J = 8.1 Hz, 1H), 7.33 (d, J = 8.0 Hz, 1H), 7.25 (d, J = 2.2 Hz, 1H), 7.12–7.02 (m, 3H), 6.97 (m, 2H), 5.64 (s, 1H), 4.39 (s, 1H), 3.28 (m, 2H), 3.14 (m, 2H), 2.83 (h, J = 7.8 Hz, 2H) ppm; ^{13}C-NMR (125 MHz, d_6 -DMSO) δ 196.8, 156.6, 136.2, 127.0, 126.7, 125.1, 122.9, 121.2, 121.0, 118.6, 118.4, 118.2, 112.2, 111.6, 111.5, 106.2, 59.0, 29.6, 27.5, 24.9 ppm, 1 unresolved aromatic ^{13}C; IR (solid) v_{max} 3426 (w, N-H), 3322 (w, N-H), 2926, 2852 (m, C-H), 1674 (s, C=O), 1625 (m, NH_3Cl), 1576 (m, NH_3Cl, C=C), 1459 (s, C-N, CH_2), 1422 (m, C=C), 1337 (m, C-N, CH_2), 1215 (w, C-H) 1098, 1073, 1038, 1001 (m, C-N, C-H), 920 (m, C-H), 792 (w, C-H), 741 (s, indole C-H) cm^{-1}; LCMS (+ESI) *m/z* 364.3 [M+H]$^+$, retention time 1.86 minutes, (100%); HRMS (+ESI): *m/z* (Calcd. $C_{21}H_{22}N_3OS$ [M+H]$^+$ = 364.1484), *Obs.* 364.1470 (δ ppm = 3.8).

Benzo[*d*]thiazol-2-ylmethanethiol (37i)

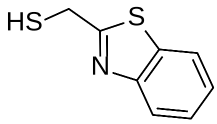

2-(Bromomethyl)-1,3-benzothioazole (228 mg, 1.00 mmol) was used in General Procedure B to yield compound **37i** as a yellow solid (110 mg, 0.61 mmol, 61%). The crude product was purified twice by flash chromatography (0-40% v/v EtOAc in Pet. ether). R_f 0.7 (10% v/v MeOH in DCM); ^{1}H-NMR (400 MHz, $CDCl_3$) δ 8.00 (d, J = 8.0 Hz, 1H), 7.86 (dd, J = 8.0, 0.7 Hz, 1H), 7.48 (ddd, J = 8.3, 7.2, 1.3 Hz, 1H), 7.39 (ddd, J = 8.3, 7.3, 1.2 Hz, 1H), 4.30 (s, 2H) ppm; ^{13}C-NMR (100 MHz, $CDCl_3$) δ 167.4, 153.0, 135.8, 126.5, 125.6, 123.2, 121.8, 41.2 ppm; IR (solid) v_{max} 3052, 2976, 2919 (w, C-H), 1939 (w, S-H), 1590 (w), 1558 (w, C=C), 1501 (s, C=N), 1455 (m, CH_2), 1432 (s), 1395 (m, C-N), 1311 (C-N), 1244, 1200 (w, CH_2), 1093, 1061, 1011 (s, C-H), 896 (m, C-H), 755 (s, C-H), 727 (s, C-H) cm^{-1}; LCMS (+ESI) *m/z* 182.1 [M+H]$^+$, retention time 2.86 minutes, (100%); HRMS (+ESI) *m/z* (Calcd. $C_8H_7NS_2$ [M+H]$^+$ = 404.1409), *Obs.* 182.0099 (δ ppm = 0.5).

S-(Benzo[*d*]thiazol-2-ylmethyl) *(R)*-2-((tert-butoxycarbonyl)amino)-3-(1*H*-indol-3-yl)propanethioate (38i)

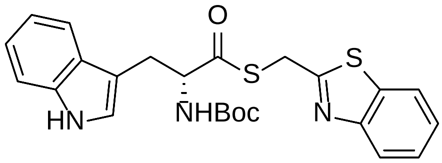

Thiol **37i** (82.7mg, 0.46 mmol) was added to a solution of *N*-Boc-*D* -tryptophan (116.5 mg, 0.38 mmol) in anhydrous DMF (3.8 mL) and the reaction mixture was cooled to 0 °C. PyBOP (297.2 mg, 0.57 mmol) was added, followed by DIPEA (0.17 mL, 0.95 mmol), and the reaction was stirred at 0 °C for 40 minutes before being allowed to warm to room temperature and stirred for a further 3 hours. The reaction was then concentrated under reduced pressure, diluted with DCM (40 mL) and washed with 10% LiCl solution (2 x 15mL), followed by brine (10 mL). The organic layer was dried over anhydrous Na_2SO_4 and the solvent was removed under reduced pressure. The crude material was purified by flash chromatography (0-50% v/v EtOAc in Pet. ether) to yield compound **38i** as an orange solid (121.1 mg, 0.26 mmol, 68%). R_f 0.7 (10% v/v MeOH in DCM); ^{1}H-NMR (500 MHz, $CDCl_3$) δ 8.05 (s, br, 1H), 7.98 (d, *J* = 8.2 Hz, 1H), 7.83 (d, *J* = 8.1 Hz, 1H), 7.55 (d, *J* = 8.0 Hz, 1H), 7.48 (ddd, *J* = 8.4, 7.2, 1.3 Hz, 1H), 7.39 (ddd, *J* = 8.3, 7.2, 1.2 Hz, 1H), 7.34 (d, *J* = 8.2 Hz, 1H), 7.18 (app.t, *J* = 7.5 Hz, 1H), 7.07 (app.t, *J* = 7.5 Hz, 1H), 6.98 (s, 1H), 5.04 (d, *J* = 8.6 Hz, 1H), 4.77 (dt, *J* = 8.6, 5.6 Hz, 1H), 4.48 (m, 2H), 3.33 (m, 2H), 1.41-1.30 (2 x s, 9H) ppm; ^{13}C-NMR (125 MHz, $CDCl_3$) δ 200.7, 167.8, 155.3, 152.7, 136.2, 135.8, 127.7, 126.4, 125.5, 123.3, 123.0, 122.5, 121.8, 120.0, 118.8, 111.3, 109.5, 80.7, 60.8, 31.2, 28.4, 27.9 ppm; IR (solid) v_{max} 3325 (w, br, N-H), 2974, 2924 (w, C-H), 2368 (w), 1690 (s, br, C=O), 1492 (m, C=N, N-H), 1457 (m, CH_2), 1435 (m, C=C), 1367 (m, CH_3, C-N), 1249 (m, C-N), 1161 (s, C-O, CH_2), 1095, 1063 (w, C-O, C-H), 850 (w, C-H), 759 (s, C-H), 742 (s, indole C-H), 729 (s, C-H) cm^{-1}; LCMS (-ESI) *m/z* 466.3 $[M-H]^-$, retention time 2.40 minutes, (100%); HRMS (+ESI) *m/z* (Calcd. $C_{24}H_{26}N_3O_3S_2$ $[M+H]^+$ = 468.1410), *Obs.* 468.1404 (δ ppm = 1.3).

S-(Benzo[*d*]thiazol-2-ylmethyl) *(R)*-2-amino-3-(1*H*-indol-3-yl)propanethioate hydrochloride (22)

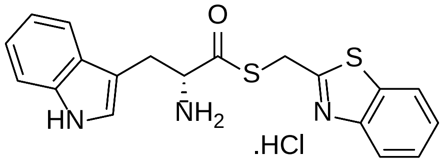

Compound **38i** (47.0 mg, 0.10 mmol) was used in General Procedure G to yield compound **22** as a white solid (48.1 mg, quant.). ^{1}H-NMR (500 MHz, d_6-DMSO) δ 11.13 (s, 1H), 8.77 (d, *J* = 5.1 Hz, 3H), 8.06 (d, *J* = 7.9 Hz, 1H), 7.95 (d, *J* = 8.0 Hz, 1H), 7.58 (d, *J* = 7.9 Hz, 1H), 7.51 (t, *J* = 7.4 Hz, 1H), 7.45 (t, *J* = 7.5 Hz, 1H), 7.36 (d, *J* = 8.1 Hz, 1H), 7.30 (d, *J* = 2.3 Hz, 1H), 7.06 (t, *J* = 7.5 Hz, 1H), 6.91 (t, *J* = 7.4 Hz, 1H), 4.65 (s, 2H), 4.57 (m, 1H), 3.34 (h, *J* = 8.4, 7.6 Hz, 2H) ppm; ^{13}C-NMR (125 MHz, d_6-DMSO) δ 195.4, 166.9, 152.3, 136.2, 135.2, 126.9, 126.4, 125.4, 125.3, 122.5, 122.2, 121.2, 118.6, 118.2, 111.5, 106.0, 66.3, 58.7, 30.6, 27.3 ppm; IR (solid) v_{max} 3223 (w, br, N-H), 2916, 2849 (m, br, C-H), 2548 (m, br, NH_3Cl), 1840 (w), 1689 (C=O), 1585 (m, C=C, C=N), 1492 (m, N-H), 1439 (m, C=C), 1237 (m, C-N), 1116 (m, C-H), 1046 (w, C-H), 986 (m), 915 (m), 870 (w), 745 (s, indole, C-H), 688 (w, C-S) cm^{-1}; LCMS (+ESI) *m/z* 368.1 $[M+H]^+$, retention time 1.75 minutes, (95%); HRMS (+ESI) *m/z* (Calcd. $C_{19}H_{18}N_3OS_2$ $[M+H]^+$ = 368.0886), *Obs.* 368.0882 (δ ppm = 1.1).

4-(Mercaptomethyl)quinolin-2(1*H*)-one (37j)

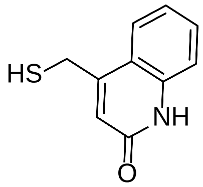

2-(Bromomethyl)quinolone-2(1*H*)-one (476.2 mg, 2 mmol) was used in General Procedure B to yield compound **37j** as a white solid (232.5 mg, 1.22 mmol, 61%). The crude product was purified by flash chromatography (0-10% v/v MeOH in DCM). R_f 0.6 (10% v/v MeOH in DCM); ^{1}H-NMR (400 MHz, d_6-DMSO) δ 11.68 (s, 1H), 7.81 (dd, J = 8.2, 1.3 Hz, 1H), 7.50 (ddd, J = 8.3, 7.1, 1.3 Hz, 1H), 7.32 (dd, J = 8.3, 1.2 Hz, 1H), 7.20 (ddd, J = 8.2, 7.1, 1.2 Hz, 1H), 6.55 (s, 1H), 3.94 (d, J = 8.0 Hz, 2H), 3.07 (t, J = 8.0 Hz, 1H) ppm; ^{13}C-NMR (100 MHz, d_6-DMSO) δ 161.6, 150.6, 139.2, 130.4, 124.8, 121.6, 120.3, 117.6, 115.7, 24.3 ppm; IR (solid) v_{max} 3103 (w, N-H), 2957 (m, br, C-H, N-H, O-H), 2848 (m, C-H), 2518 (m, S-H), 1978 (w), 1645 (s, C=O), 1611 (s, C=N), 1551 (s, N-H), 1505 (m, C=C), 1432 (s, CH_2), 1397 (s, C-N), 1385 (m, C-N), 1277, 1261, 1239 (w, C-N, C-H), 1193 (w, C-O, CH_2), 1038 (w, C-O), 988 (w, C-H), 909 (s, N-H), 871 (s, C-H), 754 (s, C-H), 670 (s, C-S) cm^{-1}; LCMS (+ESI) *m/z* 192.2 [M+H]$^+$, retention time 1.52 minutes, (92%); HRMS (+ESI) *m/z* (Calcd. $C_{10}H_9NOS$ [M+H]$^+$ = 192.0483), *Obs.* 192.0486 (δ ppm = 1.6).

S-((2-Oxo-1,2-dihydroquinolin-4-yl)methyl) *(R)*-2-((tert-butoxycarbonyl)amino)-3-(1*H*-indol-3-yl)propanethioate (38j)

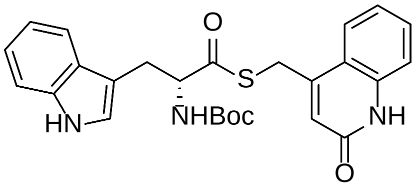

N-Boc-*D*-tryptophan (374.6 mg, 1.23 mmol) and thiol **37j** (160.4 mg, 0.839 mmol) were used in General Procedure C. The crude product was purified twice by flash chromatography (0-10% v/v MeOH in DCM). The solvent was removed and then the residue was redissolved in DCM (70 mL), washed with brine (7 mL), dried over anhydrous Na_2SO_4, and the solvent was removed under reduced pressure to yield compound SHG090 as white solid (297.8 mg, 0.623 mmol, 74%). R_f 0.6 (10% v/v MeOH in DCM); ^{1}H-NMR (400 MHz, d_6-DMSO) δ 11.75 (s, 1H), 10.85 (d, J = 2.4 Hz, 1H), 7.68 (m, 1H), 7.51 (m, 2H), 7.33 (d, J = 8.2, 1.5 Hz, 2H), 7.19 (ddd, J = 8.3, 7.1, 1.2 Hz, 1H), 7.14 (d, J = 2.3 Hz, 1H), 7.06 (app.t, J = 7.4 Hz, 1H), 6.98 (app.t, J = 7.5 Hz, 1H), 6.57 (s, 1H), 5.58 (d, J = 8.0 Hz, 1H), 4.30 (m, 2H), 4.20 (m, 1H), 3.17 (dd, J = 14.6, 4.3 Hz, 1H), 2.97 (dd, J = 14.6, 10.3 Hz, 1H), 1.28-1.23 (2 x s, 9H) ppm; ^{13}C-NMR (100 MHz, d_6-DMSO) δ 201.3, 161.4, 156.7, 155.4, 146.8, 139.1, 136.2, 130.6, 127.0, 124.5, 123.9, 121.8, 121.5, 121.1, 118.1, 118.0, 115.8, 111.5, 109.6, 78.8, 61.8, 28.9, 28.2, 27.0 ppm; IR (solid) v_{max} 3265 (w, N-H), 2928 (w, C-H), 2850 (w, C-H), 1708 (m, C=O), 1651 (s, C=O), 1634 (s, C=O), 1605 (m, C=N), 1580 (m, C=C, N-H), 1551 (m, N-H, C=C), 1480 (m, C=C), 1454 (m, CH_2), 1401 (s, C=C), 1364 (m, CH_3), 1257 (w, C-N, C-H), 1160 (s, C-O, CH_2), 1085 (w, C-O, C-H), 967 (w, CH_3), 853 (w, N-H, C-H), 748 (s, C-H), 676 (w, C-S) cm^{-1}; LCMS (+ESI) *m/z* 478.2 [M+H]$^+$, retention time 2.09 minutes, (95%); HRMS (+ESI) *m/z* (Calcd. $C_{26}H_{27}N_3O_4SNa$ [M+Na]$^+$ = 500.1614), *Obs.* 500.1614 (δ ppm = 0.1).

S-((2-Oxo-1,2-dihydroquinolin-4-yl)methyl) *(R)*-2-amino-3-(1*H*-indol-3-yl)propanethioate hydrochloride (23)

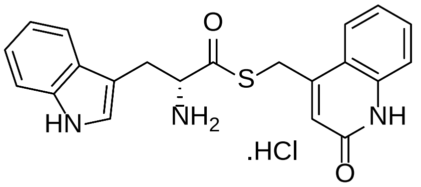

Compound **38j** (50.0 mg, 0.105 mmol) was used in General Procedure G to yield compound **23** as white solid (63 mg, quant.). R_f 0.4 (10% v/v MeOH in DCM); ^{1}H-NMR (500 MHz, d_6- DMSO) δ 11.80 (s, 1H), 11.08 (s, 1H), 8.70 (s, br, 3H), 7.61 (d, *J* = 8.0 Hz, 1H), 7.52 (app.t, *J* = 7.5 Hz, 2H), 7.35 (d, *J* = 8.1 Hz, 2H), 7.23 (d, *J* = 2.3 Hz, 1H), 7.18 (ddd, *J* = 8.2, 7.2, 1.2 Hz, 1H), 7.07 (ddd, *J* = 8.2, 7.0, 1.1 Hz, 1H), 6.95 (t, *J* = 7.2 Hz, 1H), 6.54 (s, 1H), 4.48 (m, 1H), 4.43–4.32 (m, 2H), 3.31 (m, 2H) ppm; ^{13}C-NMR (125 MHz, d_6-DMSO) δ 195.7, 161.2, 145.5, 139.0, 136.2, 130.6, 126.9, 125.2, 124.4, 122.0, 121.9, 121.2, 118.6, 118.1, 117.7, 115.8, 111.6, 106.1, 58.8, 29.4, 27.5 ppm; IR (solid) v_{max} 3300-2300 (m, br, NH_3Cl), 3256 (m, N-H), 2929, 2853 (m, br, C-H), 2611 (m, br, NH_3Cl), 1688 (m), 1646 (s, C=O), 1606 (s, C=O), 1537 (m, C=N, C=C), 1489 (m, C=C, NH_3Cl), 1432 (w, C=C, CH_2), 1366 (s, C-N), 1257 (m), 1145 (w, C-H, C-N), 1101 (w, C-H, C-H) 1011 (m, C-H), 992 (s, C-H), 918 (w, C-H), 875 (w, C-H), 752 (s, indole C-H), 733 (s, C-H), 671 (w, C-S) cm^{-1}; LCMS (+ESI) *m/z* 378.2 [M+H]$^+$, retention time 1.52 minutes, (100%); HRMS (+ESI) *m/z* (Calcd. $C_{21}H_{20}N_3O_2S$ [M+H]$^+$ = 378.1271), *Obs.* 378.1269 (δ ppm = 0.5).

(3-Phenyl-4,5-dihydroisoxazol-5-yl)methanethiol (37k)

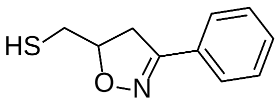

5-(Bromomethyl)-3-phenyl-4,5-dihydro-1,2-oxazole (360 mg, 1.50 mmol) was used in General Procedure B, with 5 equivalents of thiourea. The reaction was heated for 36 hours prior to the addition of 2M NaOH. The crude product was purified by flash chromatography to yield compound **37k** as a colourless oil (172.6 mg, 0.89 mmol, 60%). R_f 0.52 (1:2 v/v EtOAc:Pet. ether); ^{1}H-NMR (400 MHz, $CDCl_3$) δ 7.71–7.64 (m, 2H), 7.45–7.37 (m, 3H), 4.91 (ddt, *J* = 10.7, 7.1, 5.6 Hz, 1H), 3.46 (dd, *J* = 16.8, 10.5 Hz, 1H), 3.28 (dd, *J* = 16.7, 7.1 Hz, 1H), 2.81 (m, 2H), 1.55 (t, *J* = 8.6 Hz, 1H) ppm; ^{13}C-NMR (100 MHz, $CDCl_3$) δ 156.5, 130.3, 129.4, 128.9, 126.8, 81.6, 39.0, 28.9 ppm; IR (solid) v_{max} 3058, 2975, 2921 (w, C-H), 2569 (w, S-H), 1596, 1567 (w, C=C, C=N), 1496 (m, C-N), 1444 (m, CH_2), 1356 (m, C-H, C-O), 1243 (m, C-N), 1076 (w, C=C), 993, 916 (m, C-H), 892, 758, 693 (s, phenyl C-H), 665 (m, C-S) cm^{-1}; LCMS (+ESI) *m/z* 194.2 [M+H]$^+$, retention time 1.94 minutes, (100%); HRMS (+ESI): *m/z* (Calcd. $C_{10}H_{12}NOS$ [M+H]$^+$ = 194.0640), *Obs.* 194.0642 (δ ppm = 1.0).

S-((3-Phenyl-4,5-dihydroisoxazol-5-yl)methyl) (2*R*)-2-((tert-butoxycarbonyl)amino)-3-(1*H*-indol-3-yl)propanethioate (38k)

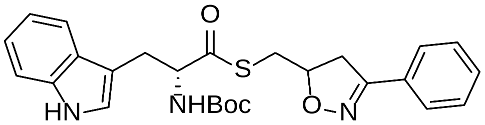

N-Boc-*D*-tryptophan (247 mg, 0.81 mmol) and compound **37k** (172.6 mg, 0.89 mmol) were used in General Procedure C to yield compound **38k** as a white amorphous solid and a 1:1 mixture of

diastereoisomers (271 mg, quant.). R_f 0.13 (25% v/v EtOAc in Pet. ether); ^{1}H-NMR (500 MHz, $CDCl_3$) δ 8.03 (2 x s, br, 1H), 7.62 (m, 2H), 7.55 (dd, J = 7.5, 5.1 Hz, 1H), 7.41 (m, 3H), 7.33 (app.t, J = 7.6 Hz, 1H), 7.19 (ddd, J = 8.2, 7.0, 1.2 Hz, 1H), 7.12 (ddt, J = 8.2, 7.1, 1.2 Hz, 1H), 7.02 (dd, J = 12.2, 2.4 Hz, 1H), 5.04 (d, J = 8.5 Hz, 1H), 4.81 (m, 1H), 4.71 (m, 1H), 3.40–3.17 (m, 4H), 3.12 (ddd, J = 14.1, 8.0, 6.3 Hz, 1H), 2.97 (ddd, J = 33.6, 16.8, 7.2 Hz, 1H), 1.42 (s, 3H), 1.38 (s, 5H) ppm; ^{13}C-NMR (125 MHz, $CDCl_3$) δ 201.6, 201.5, 156.6, 155.3, 136.3, 130.3, 129.4, 128.9, 127.7, 126.9, 123.2, 122.6, 119.9, 119.0, 118.9, 111.4, 109.7, 80.6, 79.4, 61.0, 60.9, 39.4, 39.3, 32.8, 32.7, 28.5, 28.4, 28.2, 28.1 ppm; IR (solid) ν_{max} 3334 (w, br, N-H), 2972, 2930 (w, C-H), 1685 (s, br, C=O), 1496 (m, N-H), 1457 (w, CH_2), 1446 (w, C=C), 1365 (m, CH_3), 1355 (m, C-H, C-N), 1248 (m, C-N), 1161 (m, CH_2), 1090, 1058, 1010 (w, C-N, C-H), 903, 854 (w, C-H), 759, 742 (s, indole C-H), 691 (s, phenyl C-H), 664 (w, C-S) cm^{-1}; LCMS (-ESI) m/z 478.2 [M-H]$^-$, retention time 2.39 minutes, (100%); HRMS (+ESI): m/z (Calcd. $C_{26}H_{30}N_3O_4S$ [M+H]$^+$ = 480.1952), *Obs.* 480.1960 (δ ppm = 1.6).

S-((3-Phenyl-4,5-dihydroisoxazol-5-yl)methyl) (2*R*)-2-amino-3-(1*H*-indol-3-yl)propanethioate hydrochloride (25)

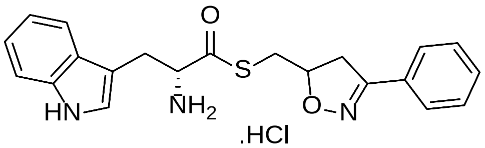

Compound **38k** (64.8 mg, 0.135 mmol) was used in General Procedure G to yield compound **25** as a pale-brown solid which was a 1:1 mixture of diastereoisomers (60.4 mg, 0.145 mmol, quant.). ^{1}H-NMR (500 MHz, d_6-DMSO) δ 11.12 (s, 1H), 8.71 (s, 3H), 7.62 (m, 2H), 7.57 (dd, J = 14.8, 7.9 Hz, 1H), 7.46 (m, 3H), 7.37 (dd, J = 7.9, 6.0 Hz, 1H), 7.28 (dd, J = 6.2, 2.0 Hz, 1H), 7.08 (app.q, J = 7.1 Hz, 1H), 7.01 (app.t, J = 7.4 Hz, 1H), 4.76 (dq, J = 12.2, 6.1 Hz, 0.5H), 4.65 (dq, J = 12.3, 6.4 Hz, 0.5H), 4.47 (s, br, 1H), 3.45 (m, 0.5H), 3.30 (m, 2.5H), 3.21 (d, J = 5.8 Hz, 1H), 3.15 (dd, J = 17.1, 6.2 Hz, 1H), 3.08 (dd, J = 17.3, 6.9 Hz, 0.5H), 3.00 (dd, J = 17.2, 6.5 Hz, 0.5H) ppm; ^{13}C-NMR (125 MHz, d_6-DMSO) δ 196.4, 196.2, 156.6, 156.6, 136.2, 130.2, 129.0, 128.8, 126.9, 126.6, 125.2, 125.2, 121.2, 118.7, 118.2, 118.1, 111.6, 106.2, 106.1, 78.6, 78.5, 58.9, 58.9, 38.8, 32.3, 32.1, 27.5, 27.4 ppm; IR (solid) ν_{max} 3276 (w, N-H), 3000-2500 (m, br, NH_3Cl), 2845 (m, C-H), 1684 (s, C=O), 1583 (w, C=C, N-H), 1496 (w, C=N), 1432 (m, CH_2), 1355 (m, C-N) 1236 (w, C-N), 1099 (m, C-H, C-N), 1075, 1041 (w, C-H, C-N), 984, 901 (m, C-H), 761 (m, C-H), 739 (s, indole C-H), 691 (s, phenyl C-H), 664 (w, C-S) cm^{-1}; LCMS (+ESI) m/z 380.3 [M+H]$^+$, retention time 1.86 minutes, (100%); HRMS (+ESI) m/z (Calcd. $C_{21}H_{21}N_3O_2SNa$ [M+Na]$^+$ = 402.1252), *Obs.* 402.1263 (δ ppm = 2.7).

***tert*-Butyl *(R)*-(1-((4-hydroxyphenethyl)amino)-3-(4-hydroxyphenyl)-1-oxopropan-2-yl)carbamate (39a)**

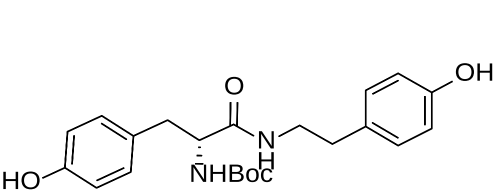

N-Boc-*D*-tyrosine (174 mg, 0.62 mmol) and tyramine (103 mg, 0.64 mmol) were used in General Procedure D to yield compound **39a**, except that DMF (2 ml) was added as a co-solvent. The crude material was purified by flash chromatography (0-5% v/v MeOH in DCM) to yield a white amorphous solid (150 mg, 0.37 mmol, 60%). R_f 0.23 (50% v/v EtOAc in Pet. ether); ^{1}H-NMR (500 MHz, $CDCl_3$) δ 6.97 (d, J = 8.5 Hz, 2H), 6.86 (d, J = 8.0 Hz, 2H), 6.71 (m, 4H), 5.12 (d, J = 7.3 Hz, 2H), 4.15 (m, 1H),

3.39 (m, 2H), 2.96 (dd, *J* = 13.7, 5.8 Hz, 2H), 2.88 (dd, *J* = 13.7, 8.1 Hz, 2H), 2.59 (m, 2H), 1.42 (s, 9H) ppm; ^{13}C-NMR (125 MHz, $CDCl_3$) *δ* 171.3, 155.0, 154.5, 130.6, 130.4, 129.9, 115.7 (2 x ^{13}C), 80.6, 56.5, 40.8, 38.2, 34.6, 28.4 ppm, 2 unresolved ^{13}C; IR (solid) v_{max} 3295 (m, br, NH, OH), 2976 (w, CH), 2925 (w, CH), 1654 (m, C=O), 1596 (m, C=C), 1514 (s, C=C), 1366 (m, CH), 1230 (s, CO), 1160 (s, CO), 826 (m, CH) cm^{-1}; LCMS (+ESI) *m/z* 401.3 $[M+H]^+$, retention time 1.74 minutes, (94%); HRMS (+ESI) *m/z* (Calcd. $C_{22}H_{29}N_2O_5$ = 401.2071 $[M+H]^+$) *Obs.* 401.2086 (*δ* = 3.7).

(R)-2-Amino-*N*-(4-hydroxyphenethyl)-3-(4-hydroxyphenyl)propanamide hydrochloride (6)

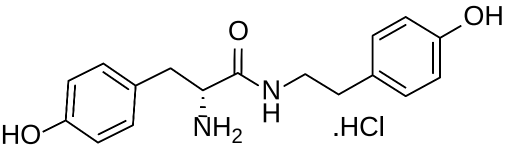

Compound **39a** (41.8 mg, 0.10 mmol) was used in General Procedure G to yield compound **6** as an off-white amorphous solid (32.7 mg, 0.097 mmol, 93%). ^{1}H-NMR (500 MHz, d_6-DMSO) *δ* 9.39 (d, *J* = 6.8 Hz, 1H), 9.24 (d, *J* = 8.0 Hz, 1H), 8.51 (dt, *J* = 19.8, 5.6 Hz, 1H), 8.18 (br s, 3H), 6.98 (d, *J* = 8.4 Hz, 2H), 6.95 (d, *J* = 8.4 Hz, 2H), 6.70 (d, *J* = 8.5 Hz, 2H), 6.68 (dd, *J* = 8.5, 1.1 Hz, 2H), 3.84 (m, 1H), 3.15-3.05 (m, 2H), 2.90 (m, 1H), 2.84 (m, 1H), 2.54 (m, 2H) ppm, ^{13}C-NMR (125 MHz, d_6-DMSO) *δ* 167.8, 156.6, 155.8, 130.5, 129.5, 129.1, 124.9, 115.3, 115.1, 53.8, 40.6, 36.2, 34.4 ppm; IR (solid) v_{max} 3300-2500 (s, br, NH_3Cl, O-H), 3020, 2930 (m, C-H), 1666 (s, C=O), 1612 (m, C=C), 1596 (m, NH_3Cl), 1566 (m, C=C), 1514 (s, N-H), 1443 (m, CH_2) 1360 (w, C-H), 1224 (s, br, C-O, C-N, CH_2), 824 (m, C-H) cm^{-1}; LCMS (+ESI) *m/z* 301.3 $[M+H]^+$, retention time 1.15 minutes, (96%); HRMS (+ESI) *m/z* (Calcd. $C_{17}H_{21}N_2O_3$ = 301.1552 $[M+H]^+$), *Obs.* 301.1558 (*δ* = 2.0).

***tert*-Butyl (*R*)-(1-amino-3-(1*H*-indol-3-yl)-1-oxopropan-2-yl)carbamate (42a)**

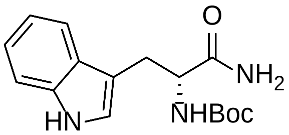

N-Boc-*D*-tryptophan (609 mg, 2 mmol) was used in General Procedure E to yield compound **42a** as a white amorphous solid (695 mg, quant.). R_f 0.33 v/v (10% MeOH in DCM); ^{1}H-NMR (500 MHz, MeOD) *δ* 7.62 (d, *J* = 8.0 Hz, 1H), 7.32 (d, *J* = 8.0 Hz, 1H), 7.08 (m, 2H), 7.01 (app. t, *J* = 7.5 Hz, 1H), 4.35 (m, 1H), 3.26 (dd, *J* = 14.5, 5.5 Hz, 1H), 3.05 (dd, *J* = 14.5, 8.0 Hz, 1H), 1.36-1.19 (2 x s, 9H) ppm, ^{13}C-NMR (125 MHz, MeOD) *δ* 177.7, 157.6, 138.1, 128.9, 124.5, 122.4, 119.8, 119.4, 112.2, 111.1, 80.6, 56.6, 29.4, 28.6 ppm; IR (solid) v_{max} 3419 (m, NH_2), 3348 (m, NH_2), 3206 (w, N-H), 2976 (w, C-H), 2917 (w, C-H), 1679 (s, C=O), 1631 (s, C=O), 1520 (s, N-H), 1458 (m, C=C, CH_2), 1366 (w, C-H, C-N), 1325 (w, C-N, CH_2), 1249 (s, C-O), 1167 (s, C-O, CH_2), 1094 (w, C-H, C-N), 1046 (w, C-H, C-N), 740 (s, indole C-H), 728 (s, C-H) cm^{-1}; LCMS (-ESI) *m/z* 302.2 $[M-H]^-$, retention time 1.72 minutes, (100%); HRMS (+ESI) *m/z* (Calcd. $C_{16}H_{22}N_3O_3$ = 304.1556 $[M+H]^+$), *Obs*. 304.1650, (*δ* ppm = 1.9).

(R)-2-Amino-3-(1*H*-indol-3-yl)propanamide hydrochloride (51a)

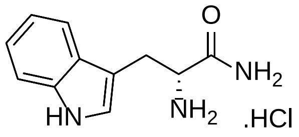

Compound **42a** (79.1 mg, 0.26 mmol) was used in General Procedure G to yield compound **51a** as a white amorphous solid, (54.6 mg, 0.23 mmol, 87%). ^{1}H-NMR (400 MHz, DMSO) δ 11.07 (s, 1H), 8.16 (s, 3H), 8.06 (s, 1H), 7.71 (d, *J* = 7.6 Hz, 1H), 7.54 (s, 1H), 7.36 (d, *J* = 8.0 Hz, 1H), 7.23 (d, *J* = 2.4 Hz, 1H), 7.08 (ddd, *J* = 8.0, 6.8, 0.8 Hz, 1H), 7.00 (ddd, *J* = 8.0, 6.8, 0.8 Hz, 1H), 3.94 (m, 1H), 3.26 (dd, *J* = 14.7, 5.9 Hz, 1H), 3.15 (dd, *J* = 14.7, 7.6 Hz, 1H) ppm; ^{13}C-NMR (100 MHz, d_6-DMSO) δ 170.3, 136.3, 127.1, 124.8, 121.1, 118.6, 118.4, 111.4, 107.1, 52.6, 27.2 ppm; IR (solid) v_{max} 3384 (m, N-H), 3248 (w, N-H), 3010 (w, N-H), 2907 (m, CH), 1691 (s, C=O), 1604 (m, C=C, N-H), 1561 (m, C=C, N-H), 1492 (s, C=C, N-H), 1460 (m, C=C), 1422 (m, C-N), 1341 (m, CH_2, C-N), 1257 (w), 1231 (w), 1136 (w), 1094 (m, C-H), 1056 (m, C-N), 1014 (m, C-N), 937 (w, C-H), 842 (m, C-H), 749 (s, indole C-H) 682 (w) cm^{-1}; LCMS (+ESI) *m/z* 204.2 [M+H]$^+$, retention time 0.46 minutes, (98%); HRMS (+ESI) *m/z* (Calcd. $C_{11}H_{14}N_3O$ = 204.1137 [M+H]$^+$), *Obs*. 204.1145, (δ ppm = 3.9).

***tert*-Butyl (*R*)-(1-amino-3-(1*H*-indol-3-yl)-1-thioxopropan-2-yl)carbamate (43a)**

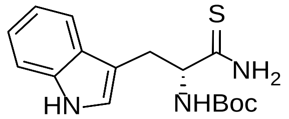

Solid $NaHCO_3$ (485 mg, 5.78 mmol) was added to a solution of compound **42a** (460 mg, 1.52 mmol) in dry DME (8 mL). P_2S_5 (676 mg, 3.04 mmol) was then added in a portion-wise manner over a period of 45 minutes to the stirred suspension. The reaction was stirred overnight at room temperature and then the solvent was removed under reduced pressure. The resulting residue was redissolved in EtOAc (30 mL), washed with H_2O (10 mL), then a saturated solution of $NaHCO_3$ (10 mL), dried over $MgSO_4$ and filtered. The solvent was removed under reduced pressure to yield compound **43a** as a pale yellow solid (470 mg, 1.47 mmol, 97%). R_f 0.23 (50% v/v EtOAc in Pet. ether); ^{1}H-NMR (500 MHz, $CDCl_3$) δ 8.14 (s, 1NH), 7.71 (dd, *J* = 8.0, 1.0 Hz, 1H), 7.35 (app. d, *J* = 8.1 Hz, 2H, (NH_2 obscured), 7.19 (ddd, *J* = 8.1, 7.0, 1.2 Hz, 1H), 7.16 (s, 1H, NH_2), 7.12 (ddd, *J* = 8.1, 7.0, 1.1 Hz, 1H), 7.07 (d, *J* = 2.4 Hz, 1H), 5.42 (s, 1H, NH), 4.72 (m, 1H), 3.36 (br, s, 1H), 3.26 (br, s, 1H), 1.40 (s, 9H) ppm; ^{13}C-NMR (125 MHz, $CDCl_3$) δ 208.9, 155.4, 136.3, 127.3, 123.5, 122.5, 120.1, 119.0, 111.4, 110.7, 80.6, 60.6, 31.9, 28.4 ppm; IR (solid) v_{max} 3420, 3382 (m, NH_2), 3194 (m, N-H), 2980, 2925 (w, C-H), 1663 (s, C=O), 1645 (s, N-H), 1525 (s, N-H), 1454 (w, C=C), 1427 (s, CH_2), 1369 (w, CH_3), 1247 (m, C-N), 1161 (s, C-O), 1089, 1011 (m, S=C), 836 (w, C-H), 736 (s, indole-C-H) cm^{-1}; LCMS (-ESI) *m/z* 318.2 [M-H]$^-$, retention time 1.95 minutes, (100%); HRMS (+ESI) *m/z* (Cald. $C_{16}H_{21}N_3O_2SNa$ = 342.1247 [M+Na]$^+$), *Obs.* 342.1244, (δ ppm = 0.9).

***tert*-Butyl *(R)*-(2-(1*H*-indol-3-yl)-1-(4-phenylthiazol-2-yl)ethyl)carbamate (44a)**

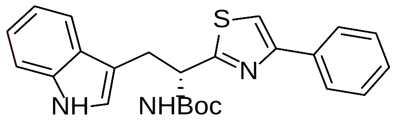

Compound **43a** (192 mg, 0.60 mmol) and 2-bromoacetophenone (143 mg, 0.72 mmol) were used in General Procedure F to yield compound **44a** as an orange solid (238 mg, 0.57 mmol, 95%). The crude product was purified by flash chromatography (20-80% v/v EtOAc in Pet. ether). R_f 0.72 (50% v/v EtOAc in Pet. ether); ^{1}H-NMR (400 MHz, $CDCl_3$) δ 8.04 (s, 1H), 7.93 (d, *J* = 7.3 Hz, 2H), 7.52 (d, *J* = 7.9 Hz, 1H), 7.45 (app.t, *J* = 7.5 Hz, 2H), 7.34 (m, 3H), 7.17 (ddd, *J* = 8.1, 7.0, 1.1 Hz, 1H), 7.07 (ddd, *J* = 7.9, 7.0, 1.0 Hz, 1H), 6.91 (s, 1H), 5.57 (s, 1H), 5.46 (s, 1H), 3.54 (m, 2H), 1.44 (s, 9H) ppm, ^{13}C-NMR (100 MHz, $CDCl_3$) δ 173.1, 155.3, 155.0, 136.1, 134.0, 128.9, 128.5, 127.9, 126.6, 123.4, 122.3, 119.8, 119.0, 112.8, 111.2, 110.7, 80.2, 53.5, 31.6, 28.5 ppm; IR (solid) v_{max} 3349 (m, br, N-H), 2977, 2921

(m, C-H), 1692 (s, C=O), 1491 (s, N-H, C=N), 1457 (m, CH_2), 1442 (w, C=C), 1366 (m, CH_3), 1247 (m, C-O), 1161 (s, CH_2), 850 (w, C-H), 1010 (m, C-H, C-N), 850 (w, C-H), 738 (s, indole C-H), 692 (m, phenyl C-H) cm^{-1}; LCMS (+ESI) *m/z* 420.3 $[M+H]^+$, retention time 2.50 minutes, (100%); HRMS (+ESI) *m/z* (Calcd. $C_{24}H_{26}N_3O_2S$ = 420.1746 $[M+H]^+$), *Obs*. 420.1748, (δ ppm = 0.5).

(R)-2-(1*H*-Indol-3-yl)-1-(4-phenylthiazol-2-yl)ethan-1-amine hydrochloride (13)

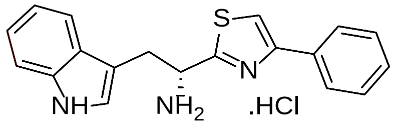

Compound **44a** (49.9 mg, 0.12 mmol) was used in General Procedure G to yield compound **13** as a brown amorphous solid (46.3 mg, quant.). ^{1}H-NMR (500 MHz, d_6-DMSO) δ 11.03 (s, 1H), 8.97 (s, 3H), 8.08 (s, 1H), 7.99 (dd, *J* = 8.3, 1.2 Hz, 1H), 7.58 (dd, *J* = 7.8, 1.0 Hz, 1H), 7.46 (dd, *J* = 8.3, 7.0 Hz, 2H), 7.36 (m, 2H), 7.16 (d, *J* = 2.6 Hz, 1H), 7.08 (ddd, *J* = 8.1, 7.0, 1.2 Hz, 1H), 6.97 (ddd, *J* = 7.9, 6.9, 1.0 Hz, 1H), 5.04 (m, 1H), 3.59 (m, 1H), 3.40 (dd, *J* = 14.2, 9.8 Hz, 1H) ppm; ^{13}C-NMR (125 MHz, d_6-DMSO) δ 165.4, 153.6, 136.2, 133.7, 128.9, 128.3, 127.0, 126.2, 124.8, 121.2, 118.6, 118.2, 115.8, 111.6, 107.4, 52.0, 29.9 ppm; IR (solid) ν_{max} 3200-2300 (m, br, NH_3Cl), 2956, 2916, 2851 (m, C-H), 1692 (w, N=C), 1594 (w, C=C, NH_3Cl), 1488 (m, N-H), 1457 (m, CH_2), 1340 (m, C-H, C-N), 1117 (s, CH_2, C-N), 870 (s, C-H), 740 (s, indole C-H), 693 (s, phenyl C-H) cm^{-1}; LCMS (+ESI) *m/z* 320.2 $[M+H]^+$, retention time 1.83 minutes, (100%); HRMS (+ESI) *m/z* Calcd. $C_{19}H_{18}N_3S$ = 320.1221 $[M+H]^+$), *Obs.* 320.1235, (δ ppm = 4.4).

tert-Butyl (1-amino-1-oxo-3-(pyridin-3-yl)propan-2-yl)carbamate (42f)

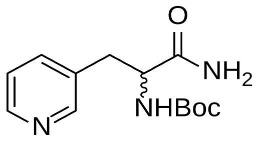

Compound **34f** (273.9 mg, 1.23 mmol) was used in General Procedure E to yield compound **42f** as a yellow solid (228.1 mg, 0.86 mmol, 83%). R_f 0.2 (10% v/v MeOH in DCM); ^{1}H-NMR (500 MHz, d_6-DMSO) δ 8.44 (d, *J* = 2.2 Hz, 1H), 8.39 (dd, *J* = 4.8, 1.6 Hz, 1H), 7.65 (d, *J* = 7.7 Hz, 1H), 7.40 (s, 1H, NH_2), 7.29 (dd, *J* = 7.8, 4.8 Hz, 1H), 7.06 (m, 1H, NH_2), 6.89 (d, *J* = 8.8 Hz, 1H, NH), 4.09 (m, 1H), 2.98 (dd, *J* = 13.8, 4.5 Hz, 1H), 2.73 (dd, *J* = 13.8, 10.3 Hz, 1H), 1.28-1 21 (2 x s, 9H) ppm; ^{13}C-NMR (125 MHz, d_6-DMSO) δ 173.2, 155.2, 150.3, 147.4, 136.6, 133.8, 123.1, 78.0, 55.2, 34.8, 28.1 ppm; IR (solid) ν_{max} 3687, 3397, 3307 (w, N-H), 2974, 2902 (s, br, C-H), 1690 (s, br, C=O), 1678 (s, br, C=O), 1525 (m, N-H), 1479, 1450 (w, C=C, C-N, CH_2), 1393, 1365 (w, CH_3, C=C), 1303 (m, C-H) 1251 (s, C=N, CH_2), 1167 (s, C-O), 1066, 1044, 1006 (s, br, C-O, C-N, C-H), 868 (w, C-H), 801, 718 (m, C-H) cm^{-1}; LCMS (+ESI) *m/z* 266.2 $[M+H]^+$, retention time 1.05 minutes, (100%); HRMS (+ESI) *m/z* (Calcd. $C_{13}H_{19}N_3O_3$ $[M+H]^+$ = 266.1505), *Obs.* 266.1539 (δ ppm = 3.0).

tert-Butyl (1-amino-3-(pyridin-3-yl)-1-thioxopropan-2-yl)carbamate (43f)

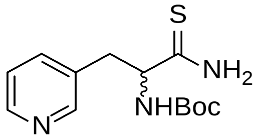

Lawesson's reagent (183.6 mg, 0.453 mmol) was added slowly a stirred solution of compound **42f** (200.7 mg, 0.757 mmol) in anhydrous THF (1.5 mL). The reaction was stirred at room temperature

for 2 hours and then the solvent was removed under reduced pressure and the residue was suspended in saturated $NaHCO_3$ (2 mL) and stirred for 30 minutes. The product was extracted into EtOAc (8 mL) and the organic fraction was washed with saturated $NaHCO_3$ (2 x 2 mL). The combined aqueous layers were further extracted with EtOAc (4 mL) and the organic layers were combined and dried over anhydrous Na_2SO_4. The solvent was removed under reduced pressure and the crude material was purified by flash chromatography (0-10% v/v MeOH in DCM) to yield compound **43f** as a white solid (84 mg, 0.30 mmol, 39%). R_f 0.2 (10% v/v MeOH in DCM); ^{1}H-NMR (400 MHz, Acetone-d_6) δ 8.94 (s, br, 1H, NH_2), 8.81 (s, br, 1H, NH_2), 8.51 (d, J = 2.3 Hz, 1H), 8.42 (dd, J = 4.7, 1.6 Hz, 1H), 7.69 (dt, J = 7.9, 2.0 Hz, 1H), 7.27 (dd, J = 7.8, 4.8 Hz, 1H), 6.18 (d, J = 8.8 Hz, 1H), 4.70 (td, J = 8.7, 5.4 Hz, 1H), 3.26 (dd, J = 13.8, 5.4 Hz, 1H), 3.01 (dd, J = 13.8, 8.8 Hz, 1H), 1.33 (s, 9H) ppm; ^{13}C-NMR (100 MHz, Acetone-d_6) δ 209.0, 155.7, 151.6, 148.8, 137.4, 134.0, 123.9, 79.5, 61.6, 39.5, 28.5 ppm; IR (solid) v_{max} 3331, 3147 (m, NH_2), 2977, 2933 (m, C-H), 1680 (s, C=O), 1646 (m, N-H), 1577 (w, C=C), 1532 (m, N-H), 1467 (m, CH_2), 1366 (m, CH_3), 1276, 1254 (m, CH_2), 1158 (s, C=S, C-O), 1047, 1016 (m, C-H, C-N), 894, 859, 841, 802, 730 (w, C-H), 711 (m, C-H) 673 (w) cm^{-1}; LCMS (+ESI) *m/z* 282.2 $[M+H]^+$, retention time 1.34 minutes, (100%); HRMS (+ESI) *m/z* (Calcd. $C_{13}H_{19}N_3O_2S$ $[M+H]^+$ = 282.1276), *Obs.* 282.1270 (δ ppm = 2.1).

***tert*-Butyl (1-(4-phenylthiazol-2-yl)-2-(pyridin-3-yl)ethyl)carbamate (44f)**

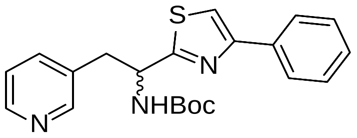

Compound **43f** (73.2 mg, 0.26 mmol) and 2-bromoacetophenone (64.7 mg, 0.33 mmol) were used in General Procedure F to yield compound **44f** as a yellow oil (47.5 mg, 0.13 mmol, 48%). The crude product was purified by flash chromatography (0-10% v/v MeOH in DCM). R_f 0.7 (10% v/v MeOH in DCM); ^{1}H-NMR (400 MHz, $CDCl_3$) δ 8.46 (d, J = 4.1 Hz, 1H), 8.41 (s, 1H), 7.88 (m, 2H), 7.50 (dt, J = 7.9, 2.0 Hz, 1H), 7.42 (m, 2H), 7.37 (s, 1H), 7.34 (tt, J = 7.5, 1.5 Hz, 1H), 7.19 (dd, J = 7.8, 4.8 Hz, 1H), 5.41 (d, J = 8.9 Hz, 1H), 5.33 (m, 1H), 3.43 (dd, J = 13.9, 6.2 Hz, 1H), 3.31 (dd, J = 14.0, 6.6 Hz, 1H), 1.42 (s, 9H) ppm; ^{13}C-NMR (100 MHz, $CDCl_3$) δ 170.2, 155.7, 155.0, 150.7, 148.2, 137.4, 134.3, 132.6, 128.9, 128.4, 126.5, 123.5, 112.9, 80.5, 53.8, 39.1, 28.4 ppm; IR (solid) v_{max} 2977, 2929 (m, C-H), 1699 (s, br, C=O), 1577 (w, C=C), 1494 (m, br, N-H), 1444, 1425 (w, C=C, CH_2, C-N, CH_3), 1366 (m, C-N, CH_3), 1273, 1249 (m, CH_2), 1161 (s, C-O), 1045, 1026 (m, C-H, C-N), 850 (w, C-H), 734, 711 (m, C-H), 601 (m, C-S), 662 (w, C-S) cm^{-1}; LCMS (+ESI) *m/z* 382.3 $[M+H]^+$, retention time 2.11 minutes, (100%); HRMS (+ESI): *m/z* (Calcd. $C_{21}H_{23}N_3O_2SNa$ $[M+H]^+$ = 404.1409), *Obs.* 404.1403 (δ ppm = 1.5).

1-(4-Phenylthiazol-2-yl)-2-(pyridin-3-yl)ethan-1-amine hydrochloride (29)

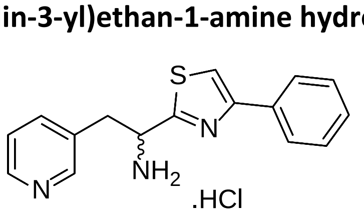

Compound **44f (**43.4 mg, 0.113 mmol) was used in General Procedure G to yield compound **29** as a yellow solid (45.3 mg, quant.). ^{1}H-NMR (500 MHz, d_6-DMSO) δ 9.15 (s, 3H), 8.93 (s, 1H), 8.80 (d, J = 5.3 Hz, 1H), 8.45 (d, J = 7.7 Hz, 1H), 8.21 (s, 1H), 7.99 (d, J = 7.2 Hz, 2H), 7.95 (app.t, J = 6.7 Hz, 1H), 7.47 (app.t, J = 7.5 Hz, 2H), 7.38 (app.t, J = 7.3 Hz, 1H), 5.31 (s, 1H), 3.64 (m, 2H) ppm; ^{13}C-NMR (125 MHz, d_6-DMSO) δ 164.4, 153.9, 145.9, 143.6, 141.3, 134.9, 133.5, 128.8, 128.4, 126.5, 126.1, 116.2,

51.5, 36.1 ppm; IR (solid) v_{max} 3397 (w, br, N-H), 3050 (w, br, C-H), 3000-2500 (s, br, NH_3Cl), 2801 (s, br, C-H), 2594 (s, NH_3Cl), 2038 (w, NH_3Cl), 1636, 1603 (m, NH_3Cl, C=N), 1560, 1520, 1489 (m, C=C, NH_3Cl), 1444 (m, C=N), 1274 (w, CH_2), 1111 (m, CH_2,C-H), 1034 (m, C-H), 946, 852 (w, C-H), 797 (m, C-H), 753 (s, C-H), 679 (s, C-S) cm^{-1}; LCMS (+ESI) *m/z* 282.2 $[M+H]^+$, retention time 1.22 minutes, (100%); HRMS (+ESI) *m/z* (Calcd. $C_{16}H_{16}N_3S$ $[M+H]^+$ = 282.1065), *Obs.* 282.1064 (δ ppm = 0.4).

tert-Butyl (1-amino-3-(5-bromo-1*H*-indol-3-yl)-1-oxopropan-2-yl)carbamate (42h)

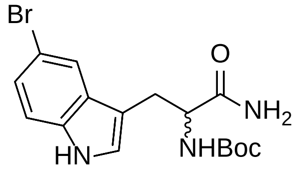

Compound **34h** (562 mg, 1.47 mmol) was used in General Procedure E to yield compound **42h** as a white solid (584 mg, 87% product by LCMS). The crude material was used in subsequent synthetic steps without further purification. R_f 0.2 (10% v/v MeOH in DCM); ^{1}H-NMR (500 MHz, d_6-DMSO) δ 11.01 (s, 1H), 7.82 (d, *J* = 2.0 Hz, 1H), 7.43 (s, 1H), 7.29 (d, *J* = 8.5 Hz, 1H), 7.19 (d, *J* = 2.4 Hz, 1H), 7.15 (dd, *J* = 8.5, 1.9 Hz, 1H), 7.01 (s, 1H), 6.68 (d, *J* = 8.5 Hz, 1H), 4.10 (td, *J* = 9.0, 4.4 Hz, 1H), 3.03 (dd, *J* = 14.5, 4.3 Hz, 1H), 2.85 (dd, *J* = 14.5, 9.6 Hz, 1H), 1.29-1.12 (2 x s, 9H) ppm; ^{13}C-NMR (125 MHz, d_6-DMSO) δ 173.8, 155.1, 134.7, 129.3, 125.5, 123.2, 121.0, 113.2, 111.1, 110.4, 77.9, 55.1, 28.1, 27.7 ppm; IR (solid) v_{max} 3441 (m, N-H), 3328 (m, br, NH_2), 3150 (w, NH_2), 2975, 2801 (m, C-H), 1701 (s, C=O), 1668 (s, C=O), 1489 (s, N-H, CH_2), 1455, 1417 (m, C=C, C-N), 1368 (m, CH_3) 1273, 1246 (m, C-H, C-N), 1166 (s, C-O, CH_2), 1065 (s, C-N, C-H), 1016 (m, C-N, C-H), 885, 798 (m, indole C-H), 675 (w, C-Br) cm^{-1}; LCMS (+ESI) *m/z* 384.2 $[M+H]^+$, retention time 1.92 minutes, (87%); HRMS (+ESI) *m/z* (Calcd. $C_{16}H_{20}N_3O_3BrNa$ $[M+Na]^+$ = 404.0586), *Obs.* 404.0582 (δ ppm = 1.0).

tert-Butyl (1-amino-3-(5-bromo-1*H*-indol-3-yl)-1-thioxopropan-2-yl)carbamate (43h)

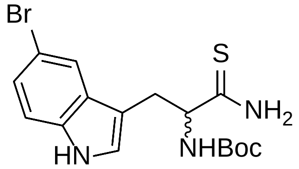

Lawesson's reagent (210.8 mg, 0.165 mmol) was added slowly a stirred solution of compound **42h** (332.2 mg, 0.869 mmol) in anhydrous THF (1.8 mL). The reaction was stirred at room temperature for 2 hours and then the solvent was removed under reduced pressure and the residue was suspended in saturated $NaHCO_3$ (2.5 mL) and stirred for 30 minutes. The product was extracted into EtOAc (10 mL) and the organic fraction was washed with saturated $NaHCO_3$ (2 x 2.5 mL). The combined aqueous fractions were further extracted with EtOAc (5 mL) and the organic layers were combined and dried over anhydrous Na_2SO_4. The solvent was removed under reduced pressure and the crude material was purified by flash chromatography (0-5% v/v MeOH in DCM) to yield compound **43h** as a white solid (215 mg, 0.54 mmol, 62%). R_f 0.5 (10% v/v MeOH in DCM); ^{1}H-NMR (400 MHz, Acetone-d_6) δ 10.72 (s, 1H, NH), 9.32 (s, 1H, NH_2), 9.16 (s, 1H, NH_2), 8.32 (d, *J* = 1.9 Hz, 1H), 7.80 (d, *J* = 8.6 Hz, 1H), 7.74 (d, *J* = 2.3 Hz, 1H), 7.65 (dd, *J* = 8.6, 1.9 Hz, 1H), 6.52 (d, *J* = 8.1 Hz, 1H, NH), 5.18 (td, *J* = 8.2, 5.6 Hz, 1H), 3.81 (dd, *J* = 14.4, 5.6 Hz, 1H), 3.64 (dd, *J* = 14.3, 8.0 Hz, 1H), 1.79 (s, 9H) ppm; ^{13}C-NMR (100 MHz, Acetone-d_6) δ 209.8, 155.7, 136.2, 130.6, 126.2, 124.7, 122.1, 114.0, 112.6, 111.4, 79.4, 61.8, 32.4, 28.5 ppm; IR (solid) v_{max} 3365 (m, NH_2), 3295 (m, N-H), 3158 (m, NH_2) 2984, 2933 (w, C-H), 1681 (s, C=O), 1659 (s, NH_2), 1495 (s, CH_2), 1422 (s, C-N), 1364 (m, CH_3), 1330 (m, CH_3), 1243 (w, C-H, C-N), 1162 (m, br, CH_2, C=S), 1065 (s, C-N, C-H), 1012 (w), 923 (w, CH_3), 880, 860, 783 (w, C-H), 741 (m, C-H), 679 (w, C-Br) cm^{-1}; LCMS (+ESI) *m/z*

398.1 [M-H]$^-$, retention time 2.12 minutes, (100%), HRMS (+ESI) *m/z* (Calcd. $C_{16}H_{20}N_2O_3BrSNa$ [M+Na]$^+$ = 420.0352), *Obs.* 420.0351 (δ ppm = 0.2).

tert-Butyl (2-(5-bromo-1*H*-indol-3-yl)-1-(4-phenylthiazol-2-yl)ethyl)carbamate (44h)

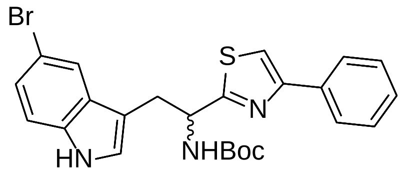

Compound **43h** (279 mg, 0.70 mmol) and 2-bromoacetophenone (175 mg, 0.88 mmol) were used in General Procedure F to yield compound **44h** as a yellow solid (273 mg, 0.55 mmol, 78%). The crude product was purified by flash chromatography (0-10% v/v MeOH in DCM). R_f 0.7 (10% v/v MeOH in DCM); ^{1}H-NMR (400 MHz, $CDCl_3$) δ 8.12 (s, 1H), 7.92 (d, *J* = 7.2 Hz, 2H), 7.56 (d, *J* = 1.7 Hz, 1H), 7.44 (app.t, *J* = 7.5 Hz, 2H), 7.36 (m, 1H), 7.33 (s, br, 1H), 7.23 (dd, *J* = 8.6, 1.8 Hz, 1H), 7.18 (d, *J* = 8.6 Hz, 1H), 6.92 (d, *J* = 2.4 Hz, 1H), 5.39 (s, 2H, CH, NH), 3.47 (m, 2H), 1.45 (s, 9H) ppm; ^{13}C-NMR (100 MHz, $CDCl_3$) δ 172.2, 155.6, 155.2, 134.7, 134.4, 129.8, 128.9, 128.3, 126.6, 125.1, 124.6, 121.7, 113.1, 112.8, 112.6, 110.6, 80.3, 53.6, 31.5, 28.5 ppm; IR (solid) v_{max} 3417 (w, N-H), 3344 (w, br, N-H), 2978, 2929 (w, C-H), 1978 (w), 1695 (s, C=O), 1488 (s, C=N, N-H), 1459 (m, CH_2), 1365 (m, CH_3), 1250 (m, C-N), 1159 (s, C-O, CH_2), 1101, 1060, 1044 (m, C-N, C-H), 976, 881, 791, 778 (m, C-H), 743 (s, indole C-H), 690 (m, C-Br), 674 (w, C-S) cm^{-1} LCMS (+ESI) *m/z* 500.2 [M+H]$^+$, retention time 2.59 minutes, (100%); HRMS (+ESI) *m/z* (Calcd. $C_{24}H_{24}N_3O_2SBr$ [M+H]$^+$ = 498.0851), *Obs.* 498.0859 (δ ppm = 1.6).

2-(5-Bromo-1*H*-indol-3-yl)-1-(4-phenylthiazol-2-yl)ethan-1-amine hydrochloride (32)

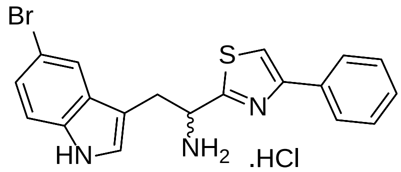

Compound **44h** (60.5 mg, 0.121 mmol) was used in General Procedure G to yield compound **32** as an orange solid (58.4 mg, quant.). ^{1}H-NMR (500 MHz, d_6-DMSO) δ 11.26 (d, *J* = 2.5 Hz, 1H), 8.89 (d, *J* = 5.3 Hz, 3H), 8.09 (s, 1H), 7.99 (dd, *J* = 8.3, 1.5 Hz, 2H), 7.75 (d, *J* = 1.8 Hz, 1H), 7.46 (app.t, *J* = 7.7 Hz, 2H), 7.37 (t, *J* = 7.4 Hz, 1H), 7.32 (d, *J* = 8.5 Hz, 1H), 7.22 (d, *J* = 2.4 Hz, 1H), 7.17 (dd, *J* = 8.6, 1.9 Hz, 1H), 5.03 (m, 1H), 3.52 (dd, *J* = 13.7, 5.0 Hz, 1H), 3.39 (dd, *J* = 14.2, 9.5 Hz, 1H) ppm; ^{13}C-NMR (125 MHz, d_6-DMSO) δ 165.3, 153.7, 134.8, 133.7, 128.8, 128.8, 128.3, 126.6, 126.2, 123.7, 120.6, 115.9, 113.5, 111.4, 107.3, 51.9, 29.8 ppm; IR (solid) v_{max} 3295 (m, N-H), 3110-2567 (br, m, NH_3Cl), 2817 (m C-H), 2610 (w, NH_3Cl), 1590 (m, N-H), 1567 (w, C=N, N-H), 1480 (m, C=N, N-H) 1459 (s, CH_2), 1360 (m, CH_2), 1318 (w, C-N), 1235 (w, C-N), 1185 (w), 1101, 1049 (s, C-H, C-N), 877 (s, C-H), 802 (s, C-H), 735 (s, indole C-H) 691 (s, C-Br), 665 (w, C-S) cm^{-1}; LCMS (+ESI) *m/z* 383.1 [M+H]$^+$, retention time 2.02 minutes, (100%); HRMS (+ESI) *m/z* (Calcd. $C_{19}H_{17}N_3BrS$ [M+H]$^+$ = 398.0327), *Obs.* 398.0320 (δ ppm = 1.8).

1-Diazo-3-(1*H*-indol-3-yl)propan-2-one (40)

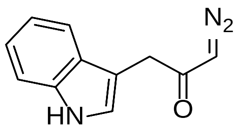

$SOCl_2$ (218 µL, 3.00 mmol) and 3 drops of dry DMF were added to a stirred suspension of indole-3-acetic acid (350 mg, 2.0 mmol) in dry THF (40 mL) at 0 °C. The reaction was allowed to stir at 0 °C for 5 hours and then additional $SOCl_2$ (20 µL, 0.28 mmol) and dry DMF (2 drops) was added. The reaction was allowed to stir for a further 20 minutes at room temperature and then the volatiles were removed under reduced pressure. The residue was diluted with dry THF (10 mL) and added immediately to a stirred solution of TMS-diazomethane (3.5 mL, 7 mmol) in dry MeCN (10 mL) at 0 °C, under a positive pressure of N_2. The reaction was stirred at 0 °C for 3.5 hours, then the solvent was removed under reduced pressure. The crude product was immediately purified by flash chromatography (10-50% EtOAc in hexane). Product containing fractions were collected and concentrated under reduced pressure. The resulting residue was redissolved in EtOAc (20 mL) and washed with a saturated solution of $NaHCO_3$ (4 x 2 mL). The solvent was then removed under reduced pressure to yield compound **40** as an orange oil (246.1 mg, 1.24 mmol, 62%). R_f 0.38 (50% v/v EtOAc in Pet. ether); ^{1}H-NMR (500 MHz, $CDCl_3$) δ 8.19 (s, 1H), 7.58 (dd, J = 7.9, 1.0 Hz, 1H), 7.39 (d, J = 8.2 Hz, 1H), 7.24 (ddd, J = 8.1, 7.0, 1.2 Hz, 1H), 7.16 (ddd, J = 8.0, 7.0, 1.0 Hz, 1H), 7.13 (d, J = 2.4 Hz, 1H), 3.78 (s, 2H) ppm; ^{13}C-NMR (125 MHz, $CDCl_3$) δ 194.2, 136.4, 127.3, 123.5, 122.7, 120.2, 118.9, 111.5, 109.3, 38.2 ppm; IR (solid) ν_{max} 3392, 3302 (w, br, N-H), 3095, 2918 (w, C-H), 2100 (s, N=N), 1713 (w, N=N), 1611 (s, br, C=O), 1456 (m, N-H, CH_2), 1421 (w, C=C), 1338 (s, br, N=N), 1228 (w, C-N, CH_2), 1123 (m, CH_2), 1092 (m, C-H), 1009 (w, C-H), 932 (w, C-H), 739 (s, indole C-H) cm^{-1}; LCMS (-ESI) *m/z* 198.1 $[M-H]^-$, retention time 1.67 minutes (100%); HRMS (-ESI) *m/z* (Calcd. $C_{11}H_8N_3O$ $[M-H]^-$ = 198.0673), *Obs.* 198.0669 (δ ppm = 2.2).

1-Bromo-3-(1*H*-indol-3-yl)propan-2-one (41)

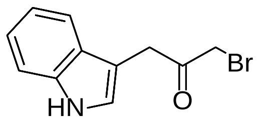

Diazoketone **40** (186 mg, 0.93 mmol) was dissolved in AcOH and the solution was cooled to 0 °C. Aqueous HBr (250 µL, 48% v/v) was added dropwise to the stirred solution and the reaction was stirred at 0 °C for 40 minutes. The reaction was periodically removed from the ice bath to ensure that the reaction remained homogenous. The reaction was then diluted with water (10 mL) and quenched with a saturated solution of $NaHCO_3$ (10 mL). The aqueous phase was extracted with DCM (3 x 30 mL), and the combined organic fractions were washed with saturated $NaHCO_3$ (5 mL), H_2O (5 mL) and brine (5 mL), then dried over anhydrous Na_2SO_4. The solvent was removed under reduced pressure to yield the crude product **41** as an orange-brown oil (205 mg). R_f 0.50 (1:2 EtOAc:Pet. ether); ^{1}H-NMR (400 MHz, $CDCl_3$) δ 8.21 (s, 1H), 7.58 (dd, J = 8.0, 1.1 Hz, 1H), 7.41 (d, J = 7.9 Hz, 1H), 7.25 (app.td, J = 8.0, 1.1 1H), 7.19 (m, 3H), 4.10 (d, J = 0.8 Hz, 2H), 3.96 (s, 2H) ppm; ^{13}C-NMR (100 MHz, $CDCl_3$) δ 200.2, 136.3, 127.1, 123.7, 122.7, 120.3, 118.7, 111.5, 107.7, 37.1, 33.6 ppm; IR (solid) ν_{max} 3224 (m, br, N-H), 3047, 2917, 2849 (w, C-H), 1704 (m, C=O), 1614 (m, N-H), 1579 (m, C=C), 1510, 1492 (w, C=C), 1456 (m, N-H, CH_2), 1423 (m, C=C, CH_2), 1336 (m, C-N, CH_2), 1204 (m, C-N, CH_2), 1011 (w, C-H), 854 (w, C-H), 744 (s, indole C-H) cm^{-1}; LCMS (+ESI) *m/z* 254.1 $[M+H]^+$, retention time 2.34, (81%).

***tert*-Butyl *(R)*-(1-(4-((1*H*-indol-3-yl)methyl)thiazol-2-yl)-2-(1*H*-indol-3-yl)ethyl)carbamate (45)**

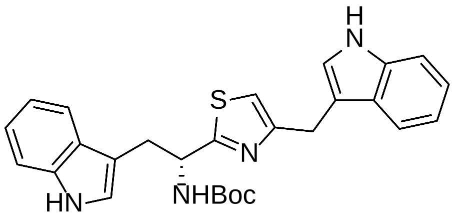

Compound **42a** (255 mg, 0.8 mmol) and crude 1-bromo-3-(1*H*-indol-3-yl)propan-2-one **41** (194.5 mg, ~ 0.77 mmol) were used in General Procedure F to yield compound **45** as yellow solid (87.1 mg, 0.18 mmol, 23% over 2 reaction steps from diazoketone **40**). The crude product was purified twice by flash chromatography (15-80% v/v EtOAc in Pet. ether, then 0-30% v/v EtOAc in DCM). R_f 0.33 (50% v/v EtOAc in Pet. ether); ^{1}H-NMR (500 MHz, $CDCl_3$) δ 8.18 (br, s, 1H), 7.91 (br, s, 1H), 7.54 (d, J = 7.9 Hz, 1H), 7.41 (d, J = 8.0 Hz, 1H), 7.38 (dd, J = 8.1, 1.0 Hz, 1H), 7.28 (d, J = 8.1 Hz, 1H), 7.21 (ddd, J = 8.2, 7.0, 1.2 Hz, 1H), 7.15 (ddd, J = 8.1, 6.9, 1.1 Hz, 1H), 7.10 (ddd, J = 8.0, 7.0, 1.0 Hz, 1H), 7.01 (ddd, J = 8.0, 7.0, 1.0 Hz, 1H), 6.97 (m, 1H), 6.69 (br, s, 1H), 6.56 (br, s, 1H), 5.37 (br, s, 2H), 4.26 (s, 2H), 3.41 (m, 2H), 1.43 (s, 9H) ppm; ^{13}C-NMR (125 MHz, $CDCl_3$) δ 172.4, 156.3, 155.2, 136.4, 135.9, 127.8, 127.3, 123.5, 122.8, 122.0, 121.9, 119.5, 119.3, 119.3, 118.7, 113.8, 113.4, 111.2, 111.0, 110.1, 80.0, 53.4, 31.5, 28.4, 27.8 ppm; IR (solid) v_{max} 3403, 3329 (w, N-H), 2976, 2916 (w, C-H), 1692 (s, br, C=N), 1619 (C=C), 1492 (s, N-H), 1455 (m, C=C, CH_2), 1365, 1337 (m, C-N, C-H), 1247, 1228 (m, C-N), 1160 (s, CH_2), 1094, 1047, 1009 (w, C-H, C-N), 907 (w, C-H), 736 (s, indole C-H) cm^{-1}; LCMS (+ESI) m/z 473.3 $[M+H]^+$, retention time 2.35, (98%); HRMS (+ESI): m/z (Calcd. $C_{27}H_{26}N_4O_2S$ $[M+H]^+$ = 473.2006), *Obs.* 473.2012 (δ ppm = 1.3).

(R)-1-(4-((1*H*-Indol-3-yl)methyl)thiazol-2-yl)-2-(1*H*-indol-3-yl)ethan-1-amine hydrochloride (18)

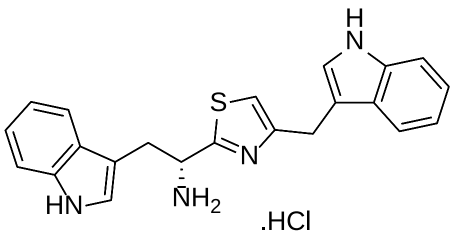

Compound **45** (63.8 mg, 0.14 mmol) was used in General Procedure G, except that additional 4 M HCl in 1,4-dixane (50 µL) was added after the reaction had stirred for 1 hour at room temperature. The reaction was allowed to stir for a further 5 minutes at room temperature and then the volatiles were removed under reduced pressure to yield compound **18** as a deep red coloured solid (56.6 mg, quant.). ^{1}H-NMR (500 MHz, d_6-DMSO) δ 11.00 (S, 1H), 10.93 (s, 1H), 8.86 (br, s, 3H), 7.46 (d, J = 7.9 Hz, 1H), 7.43 (d, J = 7.9 Hz, 1H), 7.33 (app.t, J = 8.0 Hz, 2H), 7.15–7.09 (m, 2H), 7.07–7.00 (m, 3H), 6.97–6.89 (m, 2H), 4.88 (m, 1H), 4.14 (s, 2H), 3.48 (td, J = 14.8, 14.3, 4.9 Hz, 1H), 3.31 (dd, J = 14.1, 9.6 Hz, 1H) ppm; ^{13}C-NMR (125 MHz, d_6-DMSO) δ 164.8, 155.9, 136.3, 136.1, 126.9, 126.9, 124.6, 123.6, 121.1, 121.0, 118.6, 118.5, 118.3, 118.1, 116.2, 111.5, 111.4, 107.5, 52.0, 30.2, 27.1 ppm; IR (thin film) v_{max} 3396, 3253 (m, br, N-H), 2919 (m, br, C-H), 1617, 1595 (m, C=C, C=N), 1517 (m, N-H), 1457 (s, CH_2), 1427 (m, C=C), 1339 (m, CH_2, C-N), 1226 (m), 1097, 1010 (m, C-H, C-N), 973, 933, 877, 825 (w, C-H), 741 (s, indole-C-H), 662 (w, C-S) cm^{-1}; LCMS (+ESI) m/z 373.3 $[M+H]^+$, retention time 1.86 minutes, (95%); HRMS (+ESI): m/z (Calcd. $C_{22}H_{20}N_4S$ $[M+H]^+$ = 395.1306), *Obs.* 395.1298 (δ ppm = 2.0).

tert-Butyl (*R*)-(3-(1*H*-indol-3-yl)-1-(phenethylamino)-1-thioxopropan-2-yl)carbamate (46)

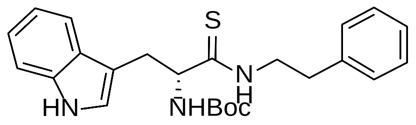

Lawesson's reagent (98 mg, 0.24 mmol) was added to a stirred solution of compound **8** (107 mg, 0.26 mmol) in anhydrous THF (5 ml) and the reaction was heated under reflux for 36 hours. The reaction was then cooled to room temperature and concentrated under reduced pressure. The crude material was purified by flash chromatography (20-80% v/v EtOAc in Pet. ether) to obtain compound **46** as an off-white amorphous solid (71.3 mg, 0.17 mmol, 64%). R_f 0.53 (50% v/v EtOAc in Pet. ether); ^{1}H-NMR (500 MHz, $CDCl_3$) δ 8.05 (s, 1H), 7.71 (d, J = 8.0 Hz, 1H), 7.38 (dt, J = 8.2, 1.0 Hz, 1H), 7.24 (ddd, J = 8.2, 7.0, 1.2 Hz, 1H), 7.20-7.08 (m, 4H), 7.03 (d, J = 2.4 Hz, 1H), 6.82 (br s, 2H), 5.57 (s, 1H), 4.57 (td, J = 8.1, 5.3 Hz, 1H), 3.74 (m, 1H), 3.57 (br s, 1H), 3.40 (dd, J = 14.5, 5.2 Hz, 1H), 3.22 (m, 1H), 2.57 (br s, 1H), 2.35 (br s, 1H), 1.42 (s, 9H) ppm, ^{13}C-NMR (125 MHz, $CDCl_3$) δ 203.1, 155.2, 137.9, 136.3, 128.9, 128.6, 127.4, 126.8, 123.2, 122.6, 120.1, 119.2, 111.4, 111.1, 80.3, 62.1, 46.6, 33.6, 32.3, 28.5 ppm; IR (solid) ν_{max} 3274 (m, br, N-H), 2977 (m, C-H), 2931 (m, C-H), 1689 (s, C=O), 1495 (s, C=C), 1455 (s, C=C), 1366 (m, C-H), 1247 (m, C-O), 1159 (s, C=S), 1064 (m, C-O, C-N), 1010 (m, C-H), 854 (w, C-H), 739 (s, indole C-H), 699 (s, phenyl C-H) cm^{-1}; LCMS (-ESI) *m/z* 422.2 [M-H]$^-$, retention time 2.46 minutes, (100%); HRMS (+ESI) *m/z* (Calcd. $C_{24}H_{30}N_3O_2S$ = 424.2053 [M+H]$^+$), *Obs.* 424.2044, (δ ppm = 2.1).

(R)-2-Amino-3-(1*H*-indol-3-yl)-*N*-phenethylpropanethioamide hydrochloride (12)

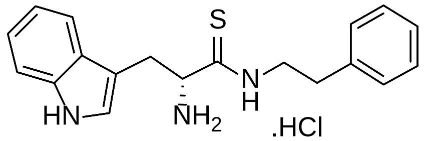

Compound **46** (42.5 mg, 0.10 mmol) was used in General Procedure G to yield compound **12** as a yellow solid (44.7 mg, 0.12 mmol, quant.). ^{1}H-NMR (400 MHz, d_6-DMSO) δ 11.04 (s, 1H), 10.71 (s, 1H), 8.40 (s, 3H), 7.66 (d, J = 7.9 Hz, 1H), 7.37 (d, J = 8.0 Hz, 1H), 7.26 (m, 2H), 7.19 (m, 2H), 7.15-7.05 (m, 2H), 7.01 (app. t, J = 7.4 Hz, 1H), 4.27 (s, 1H), 3.73 (m, 1H), 3.52 (m, 1H), 3.22 (d, J = 7.2 Hz, 2H), 2.62 (t, J = 7.6 Hz, 2H) ppm; ^{13}C-NMR (100 MHz, d_6-DMSO) δ 197.8, 138.6, 136.1, 128.5, 128.4, 127.1, 126.3, 124.7, 121.1, 118.6, 118.4, 111.4, 109.5, 107.1, 58.2, 46.5, 32.5, 30.2 ppm; IR (solid) ν_{max} 3400-2300 (m, br, NH_3Cl), 3317, 3187 (w, N-H), 2918 (m, C-H), 1544 (m, N-H), 1455 (s, CH_2), 1421 (m), 1339 (m, C-H, C-N), 1234 (w, C-N, C-H), 1153 (w, C-N, C-H), 1066 (m, br, C=S), 1009 (m, C-H), 909, 824 (w, C-H), 741 (s, indole C-H), 698 (s, phenyl C-H) cm^{-1}; LCMS (+ESI) *m/z* 324.3 [M+H]$^+$, retention time 1.67 minutes, (100%); HRMS (+ESI) *m/z* (Calcd. $C_{19}H_{22}N_3S$ [M+H]$^+$ = 324.1534), *Obs.* 324.1533, (δ ppm = 0.3).

tert-Butyl *(R)*-(1-(1*H*-indol-3-yl)-3-(phenethylamino)propan-2-yl)carbamate (47)

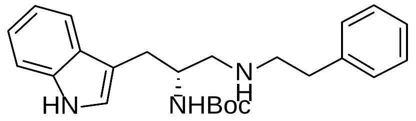

Red-Al® (Sodium bis(2-methoxyethoxy)aluminum hydride solution) (730 µl, 2.55 mmol) was added dropwise to a solution of amide 8 (204 mg, 0.50 mmol) in anhydrous THF (250 µl) and anhydrous toluene (340 µl) at 0 °C. The temperature was maintained below 20 °C throughout the addition. The reaction was then heated to 40 °C and stirred for 20 hours, before additional Red-Al (2 equiv.) was

added and the reaction was heated for a further 4 hours at 60 °C. The reaction was then cooled to 5 °C and quenched with 5M NaOH (1.1 ml, 5.50 mmol), maintaining the temperature below 25 °C. The reaction was stirred for 1 hour at room temperature, then diluted with toluene (10 ml). The phases were separated and the organic fraction was washed with 5 M NaOH (2 × 10 ml) and then concentrated under reduced pressure. The crude product was purified by flash chromatography (0-10% v/v MeOH in DCM) to yield compound **47** as a white amorphous solid (67.8 mg, 0.17 mmol, 34%). R_f 0.07 (10% v/v MeOH in DCM); ^{1}H-NMR (400 MHz, $CDCl_3$) *δ* 8.13 (s, 1H), 7.60 (d, *J* = 7.9 Hz, 1H), 7.35 (d, *J* = 8.1 Hz, 1H), 7.30 – 7.22 (m, 2H), 7.17 (m, 4H), 7.09 (app. t, *J* = 7.5 Hz, 1H), 6.93 (d, *J* = 2.3 Hz, 1H), 4.86 (s, 1H), 4.03 (m, 1H), 3.07-2.57 (m, 8H), 1.40 (s, 9H) ppm; ^{13}C-NMR (100 MHz, $CDCl_3$) *δ* 156.2, 139.5, 136.3, 128.9, 128.9, 128.7, 127.9, 126.4, 122.8, 122.2, 119.7, 119.1, 111.8, 111.2, 79.6, 52.2, 50.8, 50.4, 35.8, 28.5 (2 x ^{13}C) ppm; IR (solid) v_{max} 3381, 3314 (w, N-H), 2908 (w, C-H), 1670 (s, C=O), 1513 (s, N-H), 1477 (w, C=C, CH2), 1364 (m, C-N, C-O), 1242 (m, C-N, C-O), 1164 (s, CH2), 1049 (m, C-H), 737 (s, indole C-H), 699 (s, phenyl C-H) cm-1; LCMS (+ESI) m/z: 394.3 [M+H]+, retention time 1.92 minutes, (100%); HRMS (+ESI) m/z (Calcd. $C_{24}H_{32}N_3O_2$ $[M+H]^+$ = 394.2489), *Obs.* 394.2495, (*δ* ppm = 1.5).

(*R*)-3-(1*H*-Indol-3-yl)-*N*1-phenethylpropane-1,2-diamine hydrochloride (16)

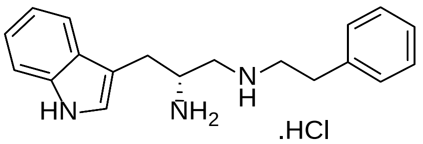

Compound **47** (40.5 mg, 0.10 mmol) was used in General Procedures G to yield compound **16** as an off-white amorphous solid, (44.7 mg, quant.). ^{1}H-NMR (400 MHz, d_6-DMSO) *δ* 11.11 (s, 1H), 9.80 (s, 1H), 9.44 (s, 1H), 8.67 (s, 3H), 7.69 (d, *J* = 7.6 Hz, 1H), 7.38 (d, *J* = 8.0 Hz, 1H), 7.35 (d, *J* = 2.3 Hz, 1H), 7.31 (m, 2H), 7.25 (d, *J* = 7.3 Hz, 1H), 7.20 (m, 2H), 7.11 (ddd, *J* = 7.9, 7.3, 0.8 Hz, 1H), 7.03 (app. t, *J* = 7.1 Hz, 1H), 3.85 (s, 1H), 3.35 – 3.13 (m, 4H), 3.05 (dd, *J* = 14.6, 8.5 Hz, 2H), 2.94 (t, *J* = 7.7 Hz, 2H) ppm, ^{13}C-NMR (100 MHz, d_6-DMSO) *δ* 136.9, 136.4, 128.6 (2 x ^{13}C), 126.9, 126.8, 124.8, 121.3, 118.6, 118.4, 111.6, 107.4, 48.6, 48.4, 48.2, 31.4, 26.9 ppm; IR (solid) v_{max} 3440, 3417 (m, N-H), 3000-2500 (m, br, NH_3Cl), 2942, 2765 (m, C-H), 1606 (w, C=C) 1496 (m, N-H), 1456 (m, CH_2), 1419, 1342 (w, C-N, C-H), 1091 (m, C-N, C-H), 1040 (w, C-H, C-N), 739 (s, indole C-H), 701 (s, phenyl C-H) cm^{-1}; LCMS (+ESI) m/z 294.3 $[M+H]^+$, retention time 1.37 minutes (100%); HRMS (+ESI) *m/z* (Calcd. $C_{19}H_{24}N_3$ $[M+H]^+$ = 294.1970), *Obs.* 294.1980, (*δ* ppm = 3.4).

***tert*-Butyl *(R)*-(2-(1*H*-indol-3-yl)-1-(5-phenyl-1*H*-imidazol-2-yl)ethyl)carbamate (48)**

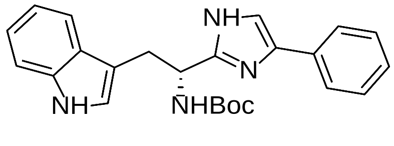

Cs_2CO_3 (163 mg, 0.5 mmol) was added to a solution of *N*-Boc-D-tryptophan (304 mg, 1.0 mmol) in absolute EtOH (2 mL) and the reaction was stirred at room temperature for 30 minutes before being concentrated under reduced pressure. The residue was redissolved in anhydrous DMF (3.6 mL) and 2-bromoacetophenone (199 mg, 1.0 mmol) was added. The reaction was stirred at room temperature for 4 hours and then the solvent was removed under reduced pressure. The residue was suspended with EtOAc (20 mL), filtered and washed with EtOAc (20 mL). The combined filtrates were concentrated under reduced pressure and the resulting yellow oil was redissolved in anhydrous *m*-xylene (12 mL). NH_4OAc (1.5 g, 1.95 mmol) was added and the reaction was heated

under reflux for 1 hour, removing excess water with a Dean-Stark trap. When complete, the reaction was cooled to room temperature, diluted with EtOAc (40 mL) and washed with water (2 x 7 mL), $NaHCO_3$ (5 mL) and brine (5 mL), then dried over Na_2SO_4 and the solvent was removed under reduced pressure. The crude product was purified by flash chromatography (0-5% v/v MeOH in DCM) to yield compound **48** as a white solid (324 mg, 0.805 mmol, 81%). R_f 0.21 (5% v/v MeOH in DCM); ^{1}H-NMR (500 MHz, $CDCl_3$) δ 8.13 (s, 1H, NH), 7.59 (br, m, 2H), 7.53 (br, d, J = 7.4 Hz, 1H), 7.35 (app. t, J = 7.7 Hz, 2H), 7.31 (d, J = 8.1 Hz, 1H), 7.24 (ddd, J =7.5, 1.8, 1.8 Hz, 1H), 7.14 (ddd, J = 8.1, 7.0, 1.1 Hz, 1H), 7.03 (ddd, J = 8.0, 7.0, 1.0 Hz, 1H), 7.00 (br, s, 1H), 6.90 (br, s, 1H), 5.80 (br, s, 1H), 5.06 (m, 1H), 3.52 (s, 1H), 3.46 (dd, J = 14.3, 7.4 Hz, 1H), 1.40 (s, 9H ppm; ^{13}C-NMR (125 MHz, $CDCl_3$) δ 156.5, 149.3, 136.2, 131.9, 128.9, 128.7, 127.5, 127.2, 125.7, 124.9, 123.3, 122.2, 119.8, 118.9, 111.3, 80.3, 50.1, 30.0, 28.5 ppm, one unresolved aromatic ^{13}C; IR (solid) ν_{max} 3397 (m, N-H), 3236, 3222 (w, N-H), 2972, 2921 (w, C-H), 1667 (s, C=O), 1608 (w, C=C), 1520 (s, N-H), 1456 (m, C-N, CH_2), 1365, 1325 (m, C-N, CH_3), 1249 (m, C-N, C-O), 1160 (s, C-O), 1016 (m, C-N), 852 (w, C-H), 759, 746 (s, indole C-H), 699 (s, phenyl C-H) cm^{-1}; LCMS (+ESI) m/z 403.3 $[M+H]^+$, retention time 1.78 min, (100%); HRMS (+ESI) m/z (Calcd. $C_{24}H_{27}N_2O_4$ $[M+H]^+$ = 403.2129), *Obs.* 403.2124 (δ ppm = 1.2).

(R)-2-(1*H*-Indol-3-yl)-1-(4-phenyl-1*H*-imidazol-2-yl)ethan-1-amine hydrochloride (14)

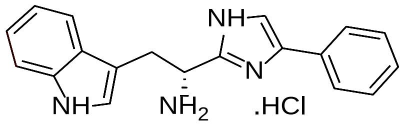

Compound **48** (61.2 mg, 0.15 mmol) was used in General Procedure G to yield compound **14** as a brown amorphous solid (54.8 mg, quant.). ^{1}H-NMR (500 MHz, d_6-DMSO) δ 11.02 (s, 1H), 9.27 (s, 3H), 8.03 (d, J = 4.8 Hz, 1H), 7.83 (d, J = 7.5 Hz, 2H), 7.59 (d, J = 7.8 Hz, 1H), 7.49 (app.t, J = 7.6 Hz, 2H), 7.40 (t, J = 7.1 Hz, 1H), 7.32 (d, J = 8.0 Hz, 1H), 7.17 (s, 1H), 7.06 (app.t, J = 7.5 Hz, 1H), 6.96 (app.t, J = 7.4 1H), 4.96 (s, 1H), 3.77 (m, 1H), 3.64 (m, 1H) ppm, ^{13}C-NMR (125 MHz, d_6-DMSO) δ 143.2, 136.2, 134.0, 129.2, 129.0, 126.8, 125.2, 124.6, 121.4, 118.7, 118.0, 115.7, 111.6, 106.5, 47.4, 28.0 ppm, one unresolved ^{13}C; IR (solid) ν_{max} 3000-2500 (m, br, NH_3Cl), 2902 (m, C-H), 2683 (m, NH_3Cl), 1635 (m, C=C, C=N), 1602 (w, C=C, C=N), 1487 (m, N-H), 1458 (m, CH_2), 1379 (m, C-H), 1076 (m, C-H, C-N), 742 (s, indole C-H), 688 (s, phenyl C-H) cm^{-1}; LCMS (-ESI) m/z 301.2 $[M-H]^-$, retention time 1.56 minutes, (98%); HRMS (+ESI) m/z (Calcd. $C_{19}H_{19}N_4$ = 303.1610 $[M+H]^+$) *Obs.* 303.1611, (δ ppm = 0.3).

tert-Butyl (*R*)-(1-hydroxy-3-(1*H*-indol-3-yl)propan-2-yl)carbamate (49)

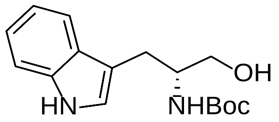

Boc-*D*-tryptophan (1.22 g, 4.00 mmol) was dissolved in dry DME (4 mL) and cooled to -15 ^{o}C. *N*-methyl morpholine (444 µL, 4 mmol) and isobutyl chloroformate (520 µL, 4 mmol) were added and the reaction was stirred at -15 ^{o}C for 1-5 minutes. The resulting white precipitate was collected by vacuum filtration and washed with DME (4 mL). The filtrate was cooled to -15 ^{o}C and then a solution of $NaBH_4$ (196 mg, 6 mmol) in H_2O (2 mL) was added immediately as a single portion. The reaction was stirred for 5 minutes and then the white precipitate was collected under vacuum. The residue was washed with ice cold H_2O (25 mL) and hexane (50 mL) and dried in vacuo to yield compound **49** as a white powder (950 mg, 3.27 mmol, 82%). R_f 0.42 (10% v/v MeOH in DCM); ^{1}H-NMR (500 MHz, Acetone-d_6) δ 9.99 (s, 1H), 7.69 (d, J = 7.9 Hz, 1H), 7.36 (d, J = 8.1 Hz, 1H), 7.17 (s, 1H), 7.08 (app. td, J = 8.1, 6.9, 1.2 Hz, 1H), 7.01 (app. td, J = 8.0, 6.9, 1.1 Hz, 1H), 5.69 (d, J = 7.6 Hz, 1H), 3.89 (m, 1H),

3.83 (m, 1H), 3.62–3.50 (m, 2H), 3.03 (dd, *J* = 14.4, 7.2 Hz, 1H), 2.95 (dd, *J* = 14.4, 6.5 Hz, 1H), 1.38 (s, 9H) ppm; ^{13}C-NMR (125 MHz, Acetone-d_6) δ 156.5, 137.6, 128.9, 124.0, 122.0, 119.6, 119.4, 112.8, 112.0, 64.1, 54.2, 28.6, 27.7 ppm; IR (solid) ν_{max} 3419, 3401, 3355 (m, O-H, N-H), 2980, 2929, 2865 (w, C-H), 1683 (s, C=O), 1525 (s, N-H), 1457 (m, CH_2), 1365 (m, C-N, CH_3), 1246 (m, C-N, C-O), 1167 (s, C-O), 1089, 1046, 999 (m, C-N, C-H), 741 (s, indole-C-H) cm^{-1}; LCMS (-ESI) *m/z* 289.2 [M-H]$^-$, retention time 1.90 min, (100%); HRMS (+ESI) *m/z* (Cald. $C_{16}H_{22}N_2O_3Na$ = 313.1523 [M+Na]$^+$), *Obs.* 313.1515, (δ ppm = 2.3).

(R)-2-Amino-3-(1*H*-indol-3-yl)propan-1-ol hydrochloride (52)

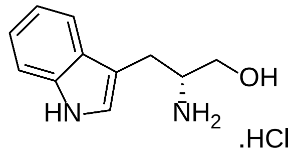

Compound **49** (66.0 mg, 0.23 mmol) was used in General Procedure G to yield compound **52** as a brown amorphous solid (62.5 mg, quant.). ^{1}H-NMR (400 MHz, d_6-DMSO) δ 11.02 (s, 1H), 8.08 (s, 3H), 7.61 (d, *J* = 6.8 Hz, 1H), 7.35 (d, *J* = 6.4 Hz, 1H), 7.24 (s, 1H), 7.08 (app. s, 1H), 7.00 (app. s, 1H), 3.55 (m, 1H), 3.46 (m, 1H), 3.30 (m, 1H), 3.05-2.92 (m, 2H) ppm; ^{13}C-NMR (100 MHz, d_6-DMSO) δ 136.2, 127.0, 124.1, 121.0, 118.4, 118.2, 111.4, 108.5, 60.1, 53.1, 24.7 ppm; IR (solid) ν_{max} 3400-2300 (s, br, NH_3Cl, O-H), 3226 (m, N-H), 2917 (m, C-H), 1597 (m, NH_3Cl), 1489 (m, N-H), 1457 (s, CH_2), 1340 (m, C-H, O-H), 1233 (w, C-H), 1046 (m, br, C-O), 743 (s, indole C-H) cm^{-1}; LCMS (+ESI) *m/z* 192.2 [M+H]$^+$, retention time 0.68 minutes, (100%); HRMS (+ESI) *m/z* (Calcd. $C_{11}H_{15}N_2O$ = 191.1184 [M+H]$^+$), *Obs*. 191.1193, (δ ppm = 4.7).

(R)-2-((*tert*-Butoxycarbonyl)amino)-3-(1*H*-indol-3-yl)propyl 3-phenylpropanoate (50)

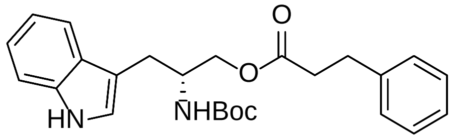

Compound **49** (174 mg, 0.60 mmol) and 3-phenylpropionic acid (90 mg, 0.60 mmol) were used in General Procedure C to yield compound **50** as a white amorphous solid (128 mg, 0.42 mmol, 71%). The crude material was purified by flash chromatography (50-100% v/v EtOAc in Pet. ether, then 0-5% v/v MeOH in EtOAc). R_f 0.71 (50% v/v EtOAc in Pet. ether); ^{1}H-NMR (500 MHz, $CDCl_3$) δ 8.04 (s, 1H), 7.62 (d, *J* = 7.5 Hz, 1H), 7.35 (dt, *J* = 8.1, 0.9 Hz, 1H), 7.31 (dd, *J* = 8.3 Hz, 2H), 7.23 (d, *J* = 7.4 Hz, 2H), 7.21-7.19 (m, 2H), 7.12 (ddd, *J* = 7.5, 0.9, 0.9 Hz, 1H), 6.93 (s, 1H), 4.63 (d, *J* = 8.7 Hz, 1H), 4.19 (s, br, 1H), 4.06 (m, 2H), 2.97 (app.t, *J* = 7.7 Hz, 3H), 2.88 (dd, *J* = 14.4, 7.7 Hz, 1H), 2.68 (t, *J* = 7.7 Hz, 2H), 1.44 (s, 9H) ppm, ^{13}C-NMR (125 MHz, $CDCl_3$) δ 172.9, 155.5, 140.5, 136.3, 128.7, 128.4, 127.8, 126.5, 122.8, 122.3, 119.8, 119.0, 111.4, 111.3, 79.6, 65.4, 49.9, 35.9, 31.0, 28.5, 27.4 ppm; IR (solid) ν_{max} 3346 (s, N-H), 2976, 2928, 2853 (w, C-H), 1715 (m, C=O), 1693 (s, C=O), 1623 (w, C=C), 1538 (m, N-H), 1457 (m, CH_2), 1440, 1367, 1340 (m, C-N, CH_3), 1267 (s, C-O), 1246 (m, C-N, C-H), 1159 (s, CH_2, C-O), 1069 (s, C-O), 1035 (m, C-H, C-N), 983, 822 (w, C-H), 741 (s, indole C-H), 697, 666 (m, C-H) cm^{-1}; LCMS (-ESI) *m/z* 421.3 [M-H]$^-$, retention time 2.57 minutes, (100%); HRMS (+ESI) *m/z* (Calcd. $C_{25}H_{31}N_2O_4$ [M+H]$^+$ = 423.2284), *Obs*. 423.2294 (δ ppm = 2.4).

(R)-2-Amino-3-(1*H*-indol-3-yl)propyl 3-phenylpropanoate hydrochloride (15)

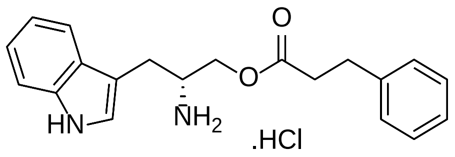

Compound **50** (53.0 mg, 0.123 mmol) was used in General Procedure G to yield compound **15** as a brown residue (52.3 mg, quant.). ^{1}H-NMR (500 MHz, d_6-DMSO) δ 11.04 (s, 1H), 8.34 (s, br, 3H), 7.59 (d, J = 7.7 Hz, 1H), 7.37 (d, J = 8.0 Hz, 1H), 7.27 (app.t, J = 7.4 Hz, 2H), 7.22 (m, 3H), 7.18 (t, J = 7.2 Hz, 1H), 7.09 (app.t, J = 7.5 Hz, 1H), 7.01 (app.t, J = 7.4 Hz, 1H), 4.14 (dd, J = 11.8, 3.5 Hz, 1H), 4.05 (dd, J = 11.9, 6.3 Hz, 1H), 3.57 (m, 1H), 3.12 (m, 1H), 2.98 (dd, J = 14.4, 9.0 Hz, 1H), 2.87 (t, J = 7.6 Hz, 2H), 2.69 (t, J = 7.7 Hz, 2H) ppm; ^{13}C-NMR (125 MHz, d_6-DMSO) δ 172.4, 140.9, 136.7, 128.8, 128.7, 127.4, 126.6, 124.9, 124.7, 121.7, 119.1, 118.6, 112.1, 108.2, 63.2, 50.5, 35.27, 30.4, 25.5 ppm; IR (solid) v_{max} 2400-2800 (br, NH_3Cl), 3326 (m, br, N-H), 3031, 2926, 2750 (m, C-H), 1715 (m, C=O), 1621, 1603 (m, N-H), 1577 (m, C=C), 1496 (m, N-H), 1456 (m, CH_2), 1240 (m, C-N, C-H), 1158 (m, C-O, C-H), 1047, 1010 (m, C-H), 891, 815 (w, C-H), 742 (s, indole C-H), 689 (m, phenyl C-H) cm^{-1}; LCMS (+ESI) *m/z* 323.3 [M+H]$^{+}$, retention time 1.71 min, (100%); HRMS (+ESI): *m/z* (Calcd. $C_{20}H_{23}N_2O_2$ [M+H]$^{+}$ = 323.1760), *Obs.* 323.1763 (δ ppm = 0.9);

***(R)*-2-Amino-3-(1*H*-indol-3-yl)propanenitrile hydrochloride**

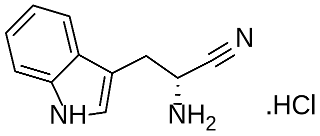

tert-Butyl *(R)*-(1-cyano-2-(1H-indol-3-yl)ethyl)carbamate (49 mg, 0.17 mmol) was used in General Procedure G to yield the title compound as a pale-brown solid (40 mg, quant.). ^{1}H-NMR (500 MHz, DMSO-d_6) δ 11.18 (s, 1H), 9.35 (s, 3H), 7.67 (d, J = 7.8 Hz, 1H), 7.39 (d, J = 8.2 Hz, 1H), 7.38 (d, J = 2.5 Hz, 1H), 7.12 (ddd, J = 8.1, 6.9, 1.2 Hz, 1H), 7.03 (ddd, J = 8.0, 6.9, 1.0 Hz, 1H), 4.74 (dd, J = 10.0, 5.2 Hz, 1H), 3.44 (m, 1H), 3.33 (m, 1H) ppm; ^{13}C-NMR (125 MHz, d_6-DMSO) δ 136.2, 126.8, 125.0, 121.4, 118.8, 118.3, 117.1, 111.7, 106.1, 41.8, 26.7 ppm; IR (solid) v_{max} 3313 (w, N-H, NH_3Cl), 3047 (w, C-H), 2922 (w, C-H), 2468 (w, CN), 2001, 1984 (m, CN), 1687 (s, N-H) 1458 (w, CH_2), 1304 (m, CH_2, C-H), 1258 (m, C-N), 1142 (m, CH_2), 1053 (m, C-H), 747 (s, indole C-H) cm^{-1}; LCMS (-ESI) *m/z* 184.2 [M-H]$^{-}$, retention time 1.12 minutes, (93%); HRMS (+ESI) *m/z* (Calcd. $C_{11}H_{12}N_3$ = 186.1026 [M+H]$^{+}$), *Obs.* 186.1025, (δ ppm = 0.4).

Molecular Biology, Biophysical and Biochemical Methods

Expression and purification of *Mtb* CYP121

The *CYP121* (*Rv2276*) gene was expressed as the His_6-tagged protein construct CYP121 using a *pHAT2/CYP121* expression clone in *E. coli* C41(DE3) cells according to the previously described conditions.[1,3] Expression was typically performed on a 10 x 1 L scale. Protein concentration and purity was determined by SDS-PAGE and amino acid analysis (Protein & Nucleic Acid Chemistry Facility [PNAC], Department of Biochemistry, University of Cambridge).

Expression and purification of Mtb CYP125, CYP126, CYP142, CYP143 and CYP144-TRV

CYP125[3] and *CYP126*[4,5] genes were expressed as the His_6-tagged protein constructs in *E. coli* C41(DE3) cells and purified according to the literature. Plasmid vectors encoding CYP125 (pET15b/*Rv3545c*) and CYP126 (pET15b/*Rv0778*) proteins were provided by Dr. Kirsty McLean

(Manchester Institute of Biotechnology, University of Manchester). His_6-tagged CYP142,[6] CYP143 and CYP144-TRV[7] proteins were prepared according to the literature and provided by Dr. Kirsty McLean (Manchester Institute of Biotechnology, University of Manchester).

Thermal Shift Assay

Samples (100 µL) comprising of CYP121 (5 µM), 2.5 x SYPRO Orange and either fragments (5 mM) or d_6-DMSO (5% v/v) were prepared in 100 mM potassium phosphate buffer (pH 6.8), in 96-well plates. Plates were centrifuged at 2000 rpm for 2 minutes prior to heating and visually inspected for precipitate. The steady-state fluorescence emission intensity (λ_{ex} 483 nm, λ_{em} 568 nm) from each sample was recorded using a BioRad iQ5 PCR System (Bio-Rad Laboratories Ltd., Hertfordshire, UK) as the temperature was increased linearly from 20–80 °C at a rate of 1 °C/min. The melting curve for CYP121 in each well was plotted (fluorescence intensity vs. temperature) and the melting point/denaturing temperature (T_m) (point of sigmoidal inflection) was identified from the maxima each curves first derivative. The difference in the melting point of CYP121 in the presence of fragments was calculated relative to the two DMSO control wells internal to each plate (T_m = 49.0 °C). Data were processed using Microsoft Excel (Microsoft Office, 2013).

Ligand Screening by UV-visible spectroscopy

UV-vis screening was performed using a CARY400 UV-vis spectrophotometer (Varian, CA, USA). CYP121 was prepared in 50 mM Tris-HCl buffer (pH 7.2), containing 1 mM EDTA. CYP125, CYP142, CYP143 and CYP144 were prepared in 50 mM Tris-HCl buffer (pH 7.5), containing 100 mM KCl. CYP126 was prepared in 100 mM potassium phosphate buffer (pH 7.5), containing 100 mM KCl. CYP124 was prepared in 100 mM potassium phosphate buffer (pH 7.0), containing 100 mM KCl. Ligands (2 µL) were prepared as d_6-DMSO stock solutions (10-200 mM) and added solutions of protein (5 µM, 198 µL), or to buffer alone (198 µL), to give a final d_6-DMSO concentration of 1% v/v. Spectra were recorded between 800-250 nm at 25 °C in a reduced volume (200 µL) quartz cuvette, with a 1 cm pathlength. Any spectral interference from the inherent absorbance of the ligands/solvent was removed by subtracting spectra collected for samples of the same ligands/solvent in buffer alone. The difference in Soret wavelength maximum ($\Delta\lambda_{max}$) of the respective P450s Soret band (λ_{max}) in the presence of ligands compared to that obtained for a 1% v/v d_6-DMSO control was used to identify type I or type II heme-binding interactions. Changes in the Soret λ_{max} less than ± 1 nm were considered within experimental error.

Competition assays UV-vis assays were performed between a known type II CYP121 ligand (clotrimazole, 50 µM) and ligands (eg **6** or tyrosine methylester hydrochloride) that did not directly perturb the Soret λ_{max} to detect binding interactions occurring distal to the heme cofactor. Novel ligands (1 µL) were added to solutions of CYP121 (5 µM, 198 µL) and clotrimazole (1 µL) to achieve a final d_6-DMSO concentration of 1% v/v. The change in the Soret λ_{max} ($\Delta\lambda_{max}$, nm) in the presence of ligands compared to that observed for CYP121 and clotimazole alone (λ_{max} = 424.5 nm) was used to assess competitive binding interactions. Buffer control spectra were subtracted from protein spectra to account for any inherent absorbance of added ligands/solvent and all solutions were inspected and found to be free of precipitate.

All spectra were generated using Origin software (OriginLab, Northampton, MA) and processed using Microsoft Excel (Microsoft Office, 2013).

Optical Titrations

Optical titrations to determine K_D values were carried out a Varian Cary 400 UV-vis spectrophotometer (Varian, CA, USA) according to a previously described procedure.[8] Assays were

performed in reduced volume (200 µL) quartz cuvettes with a path length of 1 cm (Starna, Essex, UK). Ligands were prepared as d_6-DMSO stock solutions (2.5 mM-500 mM) and proteins (5 µM) were prepared in the corresponding buffer described in the ligand screening by UV-visible spectroscopy protocol above. Aliquots (0.2 µL) of ligand stock solutions were added directly to cuvettes containing either protein solutions, or buffer alone. The final d_6-DMSO concentration did not exceed 1% v/v of the assay solution. Spectra were recorded between 800-250 nm at 25 ^{o}C after the addition of each aliquot of ligand. Buffer control spectra were subtracted from protein spectra to account for any inherent absorbance of added ligands/solvent and all solutions were inspected and found to be free of precipitate. Difference spectra were generated by subtracting the initial ligand-free protein spectrum from each successive titration spectrum. The maximum change in absorbance for each difference spectrum was then plotted against ligand concentration and fitted using a one-site binding model hyperbolic/Michaelis-Menten equation (Eqn. 1). All spectral analysis and curve fitting was performed using Origin software (OriginLab, Northampton, MA). Data were processed using Microsoft Excel (Microsoft Office, 2013).

Equation 1. $A_{obs} = (A_{max} \times L)/(K_D + L)$

In Equation 1, A_{obs} is the observed change in absorbance, A_{max} is the maximum absorbance change at saturation, L is the concentration of ligand and K_D is the dissociation constant of the enzyme-ligand complex.

Electron Paramagnetic Resonance Spectroscopy

EPR spectra for CYP121 were recorded on a Bruker ER-300D series electromagnet and microwave source interfaced with a Bruker EMX control unit and fitted with an ESR-9 liquid helium flow cryostat (Oxford Instruments) and a dual mode microwave cavity from Bruker (ER-4116DM). Spectra were recorded at 10 K with a microwave power of 2.08 milliwatts and a modulation amplitude of 10 gauss. Samples were prepared with CYP121 (100 µM), and ligands (2 mM) or DMSO (4% v/v), in 100 mM HEPES (pH 7.6) buffer, containing 100 mM NaCl and 4% glycerol. Samples were incubated for 30 minutes prior to freezing. Spectra were recorded over a wide scan (500-4500 G) and narrow scan (2000-4000 G) under the identical experimental conditions. Data analysis was performed using Origin software (OriginLab, Northampton, MA) and Microsoft Excel (Microsoft Office, 2013).

Molecular Modelling and Docking

Ligands were prepared for docking using the LigPrep, v3.2 and Epik v3.0 functions of Schrödinger suite software (Schrödinger LLC, NY).[9,10] Duplicate energy minimized (OPLS 2005) protein structures were prepared from the X-ray crystal structure of CYP121 in complex with cyclo-*L*-Tyr-*L*-Trp (cYW) (PDB 4IQ9) using the internal Protein Preparation Wizard in Maestro v10.0. Ionization states were generated to be compatible with metal-binding interactions and the heme-iron was manually adjusted to the ferric (+3) oxidation state. All water molecules were removed from one structure, while the axial heme water ligand was retained in the second protein structure. Docking grids were prepared using the structure of cYW to center the site. Ligands were allowed to dock into both grids under a range of scenarios; either employing no constraints, enforcing hydrogen bonding interactions with the axial heme water ligand or metal coordination to the ferric iron, and hydrogen bonding interactions with residue arginine 386. Docking was performed using GLIDE v6.5 (Schrödinger, LLC, NY, 2014-4) and docking figures were prepared using the PyMOL Molecular Graphics System, Version 1.3, 2010, Schrödinger, LLC.

References

[1] M. E. Kavanagh, A. G. Coyne, K. J. McLean, G. G. James, C. W. Levy, L. B. Marino, L. Pedro S. de Carvalho, D. S. H. Chan, S. A. Hudson, S. Surade, et al., *J. Med. Chem.* **2016**, *59*, 3272–3302.

[2] H. E. Seward, A. Roujeinikova, K. J. McLean, A. W. Munro, D. Leys, *J. Biol. Chem.* **2006**, *281*, 39437–39443.

[3] K. J. McLean, P. Lafite, C. Levy, M. R. Cheesman, N. Mast, I. A. Pikuleva, D. Leys, A. W. Munro, *J. Biol. Chem.* **2009**, *284*, 35524–35533.

[4] S. A. Hudson, K. J. McLean, S. Surade, Y. Q. Yang, D. Leys, A. Ciulli, A. W. Munro, C. Abell, *Angew. Chemie - Int. Ed.* **2012**, *51*, 9311–9316.

[5] S. A. Hudson, E. H. Mashalidis, A. Bender, K. J. McLean, A. W. Munro, C. Abell, *ChemBioChem* **2014**, *15*, 549–555.

[6] M. D. Driscoll, K. J. McLean, C. Levy, N. Mast, I. A. Pikuleva, P. Lafite, S. E. J. Rigby, D. Leys, A. W. Munro, *J. Biol. Chem.* **2010**, *285*, 38270–38282.

[7] M. D. Driscoll, K. J. McLean, M. R. Cheesman, T. A. Jowitt, M. Howard, P. Carroll, T. Parish, A. W. Munro, *Biochim. Biophys. Acta - Proteins Proteomics* **2011**, *1814*, 76–87.

[8] K. J. McLean, M. R. Cheesman, S. L. Rivers, A. Richmond, D. Leys, S. K. Chapman, G. A. Reid, N. C. Price, S. M. Kelly, J. Clarkson, et al., *J. Inorg. Biochem.* **2002**, *91*, 527–541.

[9] LigPrep, version 3.2, Schrödinger, LLC, New York, NY, **2014**.

[10] Epik, version 3.0, Schrödinger, LLC, New York, NY, **2014**

[10] **n.d.**
